# The evolutionary history of plant T2/S-type ribonucleases

**DOI:** 10.7717/peerj.3790

**Published:** 2017-09-11

**Authors:** Karolis Ramanauskas, Boris Igić

**Affiliations:** Department of Biological Sciences, University of Illinois at Chicago, Chicago, IL, United States of America

**Keywords:** Self-incompatibility, S-RNase, Self-incompatibility RNase, Homology, Gametophytic self-incompatibility, GSI, Gene family, Evolution, T2-RNase, Plants

## Abstract

A growing number of T2/S-RNases are being discovered in plant genomes. Members of this protein family have a variety of known functions, but the vast majority are still uncharacterized. We present data and analyses of phylogenetic relationships among T2/S-RNases, and pay special attention to the group that contains the female component of the most widespread system of self-incompatibility in flowering plants. The returned emphasis on the initially identified component of this mechanism yields important conjectures about its evolutionary context. First, we find that the clade involved in self-rejection (class III) is found exclusively in core eudicots, while the remaining clades contain members from other vascular plants. Second, certain features, such as intron patterns, isoelectric point, and conserved amino acid regions, help differentiate S-RNases, which are necessary for expression of self-incompatibility, from other T2/S-RNase family members. Third, we devise and present a set of approaches to clarify new S-RNase candidates from existing genome assemblies. We use genomic features to identify putative functional and relictual S-loci in genomes of plants with unknown mechanisms of self-incompatibility. The widespread occurrence of possible relicts suggests that the loss of functional self-incompatibility may leave traces long after the fact, and that this manner of molecular fossil-like data could be an important source of information about the history and distribution of both RNase-based and other mechanisms of self-incompatibility. Finally, we release a public resource intended to aid the search for S-locus RNases, and help provide increasingly detailed information about their taxonomic distribution.

## Introduction

Approximately one half of all flowering plant species strictly enforce outcrossing. A relatively small fraction do so through dioecy, but many more express physiological mechanisms that preferentially cause recognition and rejection of an individual’s own pollen (De Nettancourt, 1977). A great variety of such mechanisms fall under a single umbrella term—self-incompatibility (SI) systems. Despite their documented presence across angiosperms, it remains unclear how, if at all, the many interacting components of such systems are related. Dozens of independently evolved molecular mechanisms appear to cause SI, but only a few are genetically characterized or studied in great detail (Matton et al., 1994; Hiscock, Kües & Dickinson, 1996; Richman & Kohn, 1996; Franklin-Tong & Franklin, 2003; Takayama & Isogai, 2005). The best understood systems from Brassicaceae and Papaveraceae rely on unrelated genetic components, and are widely considered to be independently evolved (Nasrallah et al., 1985; Foote et al., 1994).

The genetic basis of SI in several families within the ‘core eudicots’ (The Angiosperm Phylogeny Group, 2016), which include the highly divergent Asterid and Rosid lineages, is strikingly similar. Anderson et al. (1986) discovered that in *Nicotiana alata*, the female-part recognition determinant of SI is a T2/S-type ribonuclease (S-RNase). Later, S-RNases were found to play the same role in other species of Solanaceae, and a number of species in Plantaginaceae, Rubiaceae, as well as the distantly related Rosaceae (Sassa et al., 1996; Xue et al., 1996; Nowak et al., 2011). The shared use of S-RNases has in each case hinted that the genes underlying RNase-based SI may be molecular homologs (orthologs), remarkably conserved remnants of a trait that arose in a common ancestor over 100 million years ago, whose descendants include nearly three-quarters of plant species (Xue et al., 1996; Igić & Kohn, 2001; Steinbachs & Holsinger, 2002; Nowak et al., 2011). These genes generally display a number of shared features, including expression patterns, common intron-exon site patterns, similar isoelectric points, locus structures, experience diversifying selection, and exhibit close phylogenetic relationships. The T2/S-RNase gene family is diverse and poorly functionally characterized, but eudicots appear to contain three distinct ‘classes’ of such genes, with S-RNases found exclusively in one of them, class III (Igić & Kohn, 2001). Therefore, our prior belief is strongly affected by the gene trees of T2/S-RNase family members, which form the basic core of arguments positing homology of this form of SI.

The view that RNase-based SI evolved only once was reinforced when it emerged that the male-part determinants expressed in these species are members of the same gene family, F-box motif-containing genes (SFBs, SLFs, or simplified to ‘F-boxes’; Ushijima et al., 2003; Zhou et al., 2003; Sijacic et al., 2004). Although the exact sequence of molecular interactions that lead to SI response is not completely known, painstaking studies uncovered a comically complex cascade of reactions in several unrelated species (reviewed in Liu et al., 2014 and Williams et al., 2015). Briefly, the system generally causes SI using a non-self-recognition mechanism, and it is found to operate in most RNase-based SI species examined to date, including Solanaceae, Plantaginaceae, and the Rosaceae subtribe Malinae. The term “non-self-recognition” refers to the fact that within-haplotype interactions fail to elicit a response, as outlined below in a summary of the proposed mechanism. A single S-RNase along with multiple tightly S-linked F-boxes, often spanning 10 + Mbp in a region of suppressed recombination (Lai et al., 2002; Entani et al., 2003; Williams et al., 2014; Kubo et al., 2015), comprise the self-incompatibility haplotype or “S-locus”. It has long been recognized that the male and female parts of the response must be linked, in order for the system to retain its function in face of recombination. This genetic characteristic, combined with strong negative frequency-dependent selection, ought to preserve the S-locus structure over extraordinary time scales (Ioerger, Clark & Kao, 1990). To illustrate the basic function of the system, consider a single flowering individual, with an operational system. Its diploid pistil (female) tissues expresses two S-RNase alleles in the maternal genotype. The S-RNases are proposed to freely enter any growing pollen tube (male gametophyte; Luu et al., 2000), where they generally exert a cytotoxic effect, potentially killing all pollen tubes. But once there, S-RNases encounter S-linked F-boxes, expressed by the haploid pollen (male) gametophyte. An individual can produce pollen expressing one of two alleles, each with a distinct yet overlapping set of S-linked F-boxes (Kubo et al., 2010), linked in respective allelic haplotypes (along with one S-RNase, already expressed as a part of the maternal pistil genotype). Each tandem-replicated S-linked F-box gene can inhibit a subset of S-RNases, and collaboratively they recognize and defuse all S-RNases *except* their own allelic cognate, the one tightly linked in their own haplotype (Kubo et al., 2010). Consequently, own pollen is rapidly destroyed, because the active S-RNase cleaves crucial stores of pollen tube rRNA (McClure et al., 1989). Pollen grains of other individuals in a population likely contain different S-haloptypes, and thus distinct sets of S-linked F-boxes, some of which are able to neutralize both S-RNase alleles; in our example, individual’s genotype. Generally, F-box proteins are a component of the Skp1-Cullin-F-box-type ubiquitin ligases, and copies linked to an S-RNase are thought to specifically target other S-RNases for degradation by the 26S proteasome (Qiao et al., 2004). Each haplotype of S-linked F-Boxes has the capacity to detoxify all S-RNase alleles, except the cognate, closely linked on the haplotype. This manner of non-self recognition thus ordinarily allows pollen tube growth and seed formation with pollen from unrelated individuals.

A stark exception is found in the genus *Prunus*, which is deeply nested within Rosaceae. Members of this genus express similar components that interact in a manner distinct from the mechanism sketched above. At least superficially, both in gene content and organization, *Prunus* S-haplotypes are similar to the ones found in other species with RNase-based SI, but they instead result in a pattern of interactions consistent with self-recognition. Pollen of *Prunus* species may have the capacity to neutralize all S-RNase alleles, including the one associated with a pollen grain’s own haplotype (Entani et al., 2003; Ushijima et al., 2003; Yamane et al., 2003a). Self-fertilization is seemingly prevented because S-haplotypes contain an additional inhibitor F-box gene, thought to bind self-S-RNases and prevent them from being neutralized (Yamane et al., 2003b; Ushijima et al., 2004; Tao et al., 2007). More subtle differences may include the organization of the S-locus, intron structure of the S-RNase gene, and site-specific selection pressures (Kubo et al., 2010; Kubo et al., 2015; Hauck et al., 2006; Ma & Oliveira, 2000; Vieira et al., 2007; Sutherland, Tobutt & Robbins, 2008). A number of details remain murky, as these models are highly preliminary and, for example, a general inhibitor necessary for coherence of the proposed self-recognition *Prunus* model remains unidentified. Nevertheless, these differences appear fairly profound, because we lack a sound theory to explain how minor background mutations could switch between a mechanism with non-self-recognition that inhibits S-RNase cytotoxicity to one in which self-recognition elicits S-RNase cytotoxicity, for each of several dozen segregating alleles (Matsumoto & Tao, 2016).

As a result, there is considerable disagreement in the literature over the correct interpretation and weight of evidence supporting two opposing accounts. It is possible that all RNase-based SI systems are ancestrally shared, yet show a great capacity for divergent changes in a variety of important phenomena. The contrasting and increasingly common view calls into question this account of S-RNase gene orthology and, therefore, the homologous basis of RNase-based SI. Instead, it posits the possibility of a truly exceptional functional, mechanistic, and structural convergence. Convergent recruitment of gene family members in similar adaptations is known from a growing number and variety of systems (Christin, Weinreich & Besnard, 2010).

Ideally, the evaluation of hypotheses concerning homology would involve accurately tracing the evolutionary histories of all known RNase-based SI mechanisms. But SI is a highly complex trait, whose function emerges from the interaction of multiple genetic components, many of which are unknown, and the system is very old. Faced with these obstacles, contemporary studies rely on the inferred phylogenetic and functional relationships of only some of the known genetic components, with limited sequence data, from a handful of species where they are sequenced or fully genetically characterized, and with models of evolution whose power of inference is severely limited.

Studies of the phylogenetic relationships among RNase-based SI systems, in particular, necessarily depend on gene trees of the female-expressed S-RNases, and those of other T2/S-type RNases, not involved in the SI response (Richman, Broothaerts & Kohn, 1997; Steinbachs & Holsinger, 2002). Inferences from their male-expressed counterpart S-linked F-boxes—which show patterns associated with gene conversion and concerted evolution—are exceedingly complicated, because we have little or no grasp of what constitutes an appropriate model of evolution for this locus (Innan, 2009). Evidence seems to indicate that putative S-linked F-boxes show little of the conserved trans-specific S-haplotype pattern shared with their cognate S-RNases (Kubo et al., 2015). Establishing a reasonable marker for expected divergences within particular homologous mechanisms is difficult, and no solace is to be found in the comparably simple system of Brassicaceae, where the S-locus seems capable of vast genomic rearrangements and duplications (Chantha et al., 2013). In due time, a trove of exceptions and variations may prove instructive for a variety of studies, but profound insight is currently limited to those processes that leave behind a reliable phylogenetic history. Since our last analyses (Igić & Kohn, 2001), nearly complete genomes for many species have vastly increased the number of available RNase sequences and uncovered male-part genes, but understanding of the evolutionary processes that affect this important protein family, as well as its origin of novel functions has not increased proportionally.

Here, we re-examine the strength of evidence supporting or detracting from the hypothesis that S-RNase-based SI evolved once in the core eudicots. We narrowly aim to estimate the relationships among T2/S-RNases, with an emphasis on the placement of S-RNases and, using many lines of evidence, provide a framework for classification of T2/S-RNase family of genes in plants. We assemble a large database of T2/S-type RNases and reconstruct their evolutionary history. With analyses of molecular sequences, structural features, locations, as well as the distribution of these genes across extant species, we more broadly attempt to provide the most complete picture, to date, of the relationships among T2/S-RNase members in plants, in an attempt to enable insights into the evolution of RNase-based SI. We also implement a public web service that allows other researchers to easily determine the phylogenetic placement of their own T2/S-type RNase sequences and generate functional hypotheses.

## Methods

### T2/S-RNase sequences and alignment

We obtained known T2/S-RNase amino acid sequences from the protein database of GenBank release 202 (see Section S1 for query strings). Groups of sequences that shared 90% or higher sequence identity were identified using UCLUST clustering algorithm in USEARCH version 7.0.1090, and only the longest sequence from each group was retained (Edgar, 2010). The resulting set was then used to query core nucleotide (NT) and expressed sequence tags (EST) databases with the tblastn algorithm in BLAST version 2.2.29, with default settings and an expected value cut-off of 1 × 10^−10^ (Altschul et al., 1990). The limited taxonomic search included only data from the Viridiplantae (green algae and land plants), and was restricted to sequences between 300 to 10,000 bp (see Section S2 for exact query). The BLAST results from EST and NT databases contained 3,679 and 2,380 unique accessions, respectively, from 411 species.

With the exception of *Petunia* × *hybrida* and *Solanum lycopersicum*, sequences from domesticated and hybrid species where excluded (*n* = 26, see Section S3 for a list of excluded species). The number of allelic S-RNase sequences was deliberately reduced for efficiency, and only the longest high-quality representative sequences were kept for all available genera. Coding regions from nuclear DNA and mRNA sequences from NT and EST databases were aligned using MAFFT version 7.158b (Katoh & Standley, 2013). A maximum likelihood guide tree was constructed using RAxML version 8.0.26 (Stamatakis, 2014). With the exclusion of known S-alleles, groups of monophyletic congeneric sequences that shared at least 98% sequence identity were identified using UCLUST. Sequences within each group were aligned using MAFFT version 7.158b and visually inspected in Geneious version 7 (created by Biomatters, available from http://www.geneious.com). We removed any present polyadenylation tails or ambiguous characters at either end of each sequence. Overlapping groups (unigenes) were collapsed to a majority rule consensus sequence with ambiguities introduced as necessary. The resulting set of sequences was reviewed, and only the sequences longer than 350 bp that contained at least three out of five conserved sequence motifs found in this gene family were kept. Catalytically active histidine residue (CAS II) is known to be essential to ribonucleic activity of T2-type RNases (Jost et al., 1991; Taylor et al., 1993; Irie, 1999). We identified and kept the proteins that were apparently missing this residue, as well as proteins established as catalytically inactive in functional studies (MacIntosh et al., 2010; Ohkama-Ohtsu et al., 2004; Wei et al. , 2006; Gausing, 2000; Van Damme et al., 2000; Kim et al., 2004).

In the guide tree constructed during the review process, sequences in our dataset formed two distinct clades. One of these clades consisted of T2/S-RNases, the other was composed of Thioredoxin-domain-2-containing disulphide isomerases. These sequences, excluded from analyses, were likely detected by BLAST search because they contain a sequence motif similar to T2/S-RNase conserved region 3. The processed dataset sourced from GenBank searches contained 618 sequences (see Table S1 for the list of GenBank accessions).

### Retrieval and processing of sequences from genomes

We also used the above-generated dataset to query 146 available sequenced plant genomes (as of September 2014), using the blastn algorithm (see Table S2 for the list of genomes used). The gene structures of the BLAST hit results including the adjoining upstream and downstream 3 kb segments were annotated with Exonerate 2.2.0 (Slater & Birney, 2005) using the translated GenBank dataset as templates. Annotated sequences containing premature stop codons or ambiguous amino acid residues were removed. To construct the final T2/S-RNase sequence dataset for alignment, we extracted the coding regions obtained from the genomic sequences (see Table S1 for a list of genomic sequences), concatenated them, and then pooled them with the GenBank dataset. Groups of sequences that shared 95% sequence identity (or higher) were identified using UCLUST, and then further subdivided by genus. The longest sequence from each such group was retained.

### Final sequence dataset and alignment

After the inclusion of sequences obtained from the genomes and subsequent processing, the final set consisted of 715 sequences annotated as T2/S-RNases, with detectable T2/S-RNase features, and/or grouping with T2/S-RNases in preliminary analyses. The bulk of these (711) represented land plants (embryophytes), the rest came from distantly related chlorophyte algae to be used as potential outgroups (one from *Chlamydomonas reinhardtii* and *Bryopsis maxima*, and two from *Volvox carteri*). No sequences from more closely related streptophyte algae were available. These sequences were translation-aligned using MAFFT version 7.164b, the alignment was reviewed, adjusted manually and mapped back to nucleotide sequences.

### Phylogenetic analyses

Separate gene trees were inferred using nucleotide and amino acid substitution models using MrBayes version 3.2.2 (Ronquist et al., 2012). Both analyses consisted of four independent runs with one cold and seven heated chains. We implemented general time-reversible models with Gamma-distributed among-site rate variation, and four Gamma rate categories. The runs were allowed to complete 780 and 200 million generations for nucleotide and amino acid models, respectively. The trees and parameters were sampled every 1,000 generations for both runs. The temperature parameter was periodically adjusted throughout the runs to ensure that the acceptance rates of attempted swaps between the cold and the heated chains fell within the target window of 20%–60%. The proposal probabilities for different moves were tuned so the acceptance rates fell within the target window of 20%–70%. Parameter convergence was assessed using R package RWTY (Warren, Geneva & Lanfear, 2017). For both analyses, all parameters have converged within the first fifty million generations. The tree topologies took much longer to converge, as judged with treespace plots. Based on this information, the burn-in was set to 580 million generations for nucleotide trees and 150 million generations for amino acid trees. Both posterior tree sets were used to generate maximum credibility trees as well as consensus trees with minimum clade frequency threshold of 0.75 using the program SumTrees version 3.3.1 (Sukumaran & Holder, 2010). The posterior sets from both analyses were resampled (every four million generations for nucleotide trees and every one million generations for amino acid trees) to obtain a total of 100 trees which were used as starting trees for maximum likelihood inference with RAxML version 8.1.17 (Stamatakis, 2014). Two sets of analyses were performed by fitting a general time-reversible models of nucleotide and amino acid substitution with the CAT model of rate heterogeneity. Support values for the highest scoring RAxML trees were calculated from the respective MrBayes posterior set of trees using SumTrees.

### Analysis of intron positions

In order to investigate T2/S-RNase intron/exon structure, genomic sequences with gene structure annotations were first translation-aligned using MAFFT version 7.164b in Geneious and the alignment was manually adjusted. Introns were treated as homologous across sequences if their starting positions overlapped within a seven nucleotide window in the alignment. Next, introns were classified by their phase (position within a codon). Phase zero introns occur before the first base, phase one and two introns interrupt a codon triplet after the first or second base, respectively. Aside from plants, intron positions were also identified in the T2-type RNase loci from algae (*Volvox carteri*, *Bryopsis maxima*), animals (*Amphimedon queenslandica*, *Strongylocentrotus purpuratus*, *Hydra vulgaris*, *Homo sapiens*), and (fungi *Saccharomyces cerevisiae*, *Aspergillus oryzae*).

### Isoelectric point (pI) value calculations

Isoelectric point (pI) is the pH at which a molecule, on average, carries no net electric charge. The pI value for each sequence included in the phylogenetic analyses was calculated using methods described in Bjellqvist et al., (1993) and Bjellqvist et al., (1994) implemented in the ProteinAnalysis tool in Biopython 1.64 (Cock et al., 2009). Signal peptide sequences were not included in pI calculations.

### Identification of putative SFB genes located near T2/S-RNases

F-box motif-containing genes empirically linked with SI function are co-located with S-RNases and are approximately 1 kb long (Lai et al., 2002; Ushijima et al., 2003; Sijacic et al., 2004; Entani et al., 2003). Most lack introns, although a single intron has been reported in untranslated upstream region of *Prunus avium* SFBs (Yamane et al., 2003a; Vaughan et al., 2006). Open reading frames (ORFs) between 900 bp and 1.8 kb, each containing this motif, were identified within the upstream/downstream 2 Mb regions flanking genomic RNase loci. Each ORF was used to query the GenBank NT database with an expected cutoff value of 1 × 10^−20^. Resulting hits containing the terms “f-box” or “fbox” in their descriptions were treated as potential SFB genes and were combined with known S-locus associated F-box sequences from Solanaceae, Plantaginaceae, Rubiaceae, and Rosaceae. These sequences were translation-aligned using MAFFT version 7.309 and a maximum likelihood tree was constructed using RAxML version 8.2.9, with a general time-reversible model of nucleotide substitution and CAT model of rate heterogeneity. Sequences belonging to a clade that included the known S-locus F-box sequences were extracted and realigned using MAFFT. A gene tree was constructed with RAxML using general time-reversible model of nucleotide substitution and CAT model of rate heterogeneity. Rapid bootstrap analysis was conducted using ‘-f a’ option. Two thousand bootstrap replicates were obtained.

### Online service for the phylogenetic placement of T2/S-RNases

Alignment and phylogenetic reconstruction with highly divergent sequences, like these diverse members of the T2/S-RNases, is a tedious process. In order to facilitate phylogenetic placement and classification of new T2/S-type RNase sequences, we provide an online service available at http://t2.karol.is. The service takes one or more nucleotide or amino acid sequences and adds them to the sequence alignment used in this study using MAFFT option ‘-add’. This new alignment is then fed into RAxML, which adds user provided sequences to the maximum likelihood tree obtained in this study using RAxML evolutionary placement algorithm, option ‘-f v’. The results, provided for download to the user, include the alignment and the tree files, as well as calculated isoelectric point values (for amino acid sequences), and classification of user sequences as putative members of class I, II, or III.

## Results

### Phylogenetic relationships among T2/S-RNases

**Figure 1:**
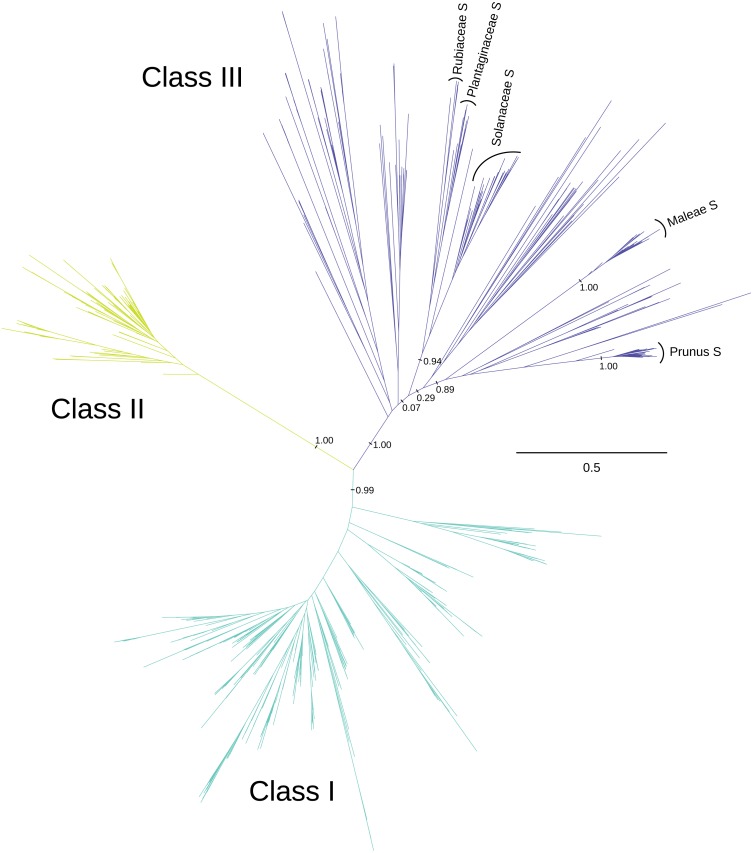
Phylogenetic relationships between three classes of T2/S-type RNases in land plants. The unrooted maximum-likelihood phylogram is shown, along with posterior support for some key recovered branches. Tip labels are omitted for clarity, and can be found in Fig. S1. S-RNase sequences from the four families with RNase-based SI are indicated, including two genera from the Rosaceae (*Prunus* and *Malus*). The scale shown illustrates the vast divergences, in units of nucleotide substitutions per site. Many pairwise distances within each class exceed one substitution per site, and many distances between classes exceed two substitutions per site. Note the low distances within class II T2/S-RNases, compared with class I, and class III.

We recover three distinct clades of T2/S-RNases (Fig. 1 and Fig. S1), which mirror the previously described three ‘classes’ (e.g., Igić & Kohn, 2001; Steinbachs & Holsinger, 2002). These clades are present in the consensus and maximum credibility trees derived from MrBayes posterior tree sets, as well as the best-scoring RAxML maximum likelihood trees, inferred using both nucleotide and amino acid alignments and substitution models. (Only the best-scoring maximum likelihood tree obtained using nucleotide alignment and substitution model is used in the figures. Trees obtained using other reconstruction methods are available as supplementary material.) The posterior support values for classes I, II, & III were 1.00, 1.00, and 0.99, respectively, in the analyses of nucleotide alignment and model of sequence evolution, and 0.96, 1.00, and 0.78 when amino acid alignment and substitution model was used. The three classes are defined somewhat arbitrarily, but are remarkably well-supported by other lines of evidence examined, and we enumerate these in turn, when they relate to our principal results. No angiosperm genome we examined contains fewer than four members of this superfamily.

The dataset contained 349 sequences inferred to belong within class I. The members of this class of sequences were represented in all major land plant lineages, including the so-called ‘early-diverging’ groups—marchantiophytes, bryophytes, lycophytes, and ferns. Several well-characterized T2/S-RNases belong to class I, such as *Arabidopsis thaliana* RNS1 and RNS3 (Bariola et al., 1994; Bariola, MacIntosh & Green, 1999; Hillwig et al., 2008; Hillwig et al., 2011; LeBrasseur et al., 2002; Nishimura et al., 2014), *Nicotiana glutinosa* RNase NW and RNase NT (Kariu et al., 1998; Hino, Kawano & Kimura, 2002; Kawano et al., 2006; Kurata et al., 2002), and *Solanum lycopersicum* RNase LE and RNase LX (Groß, Wasternack & Köck, 2004; Jost et al., 1991; Köck et al., 2004; Köck, Stenzel & Zimmer, 2006; Lers et al., 1998; Lers et al., 2006; Löffler et al., 1992; Nürnberger et al., 1990; Tanaka et al., 2000). Many genes included in this group are secreted active RNases, expressed during senescence and phosphate starvation (see Table S3 for a list of studies of T2/S-RNase expression patterns and functions).

Class II RNases are generally found as single-copy genes within the seed plants, with the exception of recent polyploids and few apparent instances of segregating paralogous copies. The dataset contained 125 sequences placed in this class, including the genes coding for *Arabidopsis thaliana* RNS2 (Taylor et al., 1993; Bariola, MacIntosh & Green, 1999; Hillwig et al., 2011), *Nicotiana glutinosa* NGR2 (Kurata et al., 2002), and *Solanum lycopersicum* RNase LER (Köthke & Köck, 2011). These genes appear often constitutively expressed, and their expression levels are not necessarily increased by wounding. We did not find any class II genes in the genomes of *Selaginella moellendorffii*, a lycophyte, or *Physcomitrella patens*, a moss (bryophyte).

On the other hand, the genome of *Marchantia polymorpha*, a liverwort, surprisingly *does* contain a single class II sequence. This is unexpected because liverworts are more distantly related to flowering plants, than are either lycophytes or mosses. The discordance could be due to a variety of errors (e.g., flawed genome assemblies), independent losses in lycophytes and mosses, or unusual evolutionary processes (e.g., horizontal transfer). The possibilities could be disentangled with a broader phylogenetic coverage, but no fully sequenced fern genome has been published to date, and all T2/S-RNase sequences from ferns deposited in GenBank cluster with our class I. Finally, we found no class II sequences in the draft genomes of *Salvinia cucullata* and *Azolla filiculoides* (F-W Li, pers. comm., 2017).

Class III RNases are comprised by S-RNases and an astonishing diversity of non-S-RNase sequences. The occurrence of this group is restricted to core eudicots (The Angiosperm Phylogeny Group, 2016), although they are not present in all families whose representatives have been sequenced to date. Most notably, class III members appear absent from the well-characterized genomes in *Arabidopsis* and *Brassica*, although a distant relative, *Carica papaya* (Caricaceae, Brassicales), does contain a putative class III gene. Class III sequences were similarly absent from the published genome assembly of *Lactuca sativa* (Asteraceae).

Our dataset contained 237 class III sequences. More than a half of these (122) were specifically included because of their reportedly known S-RNase identity and function. Without exception, all functional S-RNases belong to class III, but they form an apparently polyphyletic group, although this assignment is complicated (see ‘Discussion’; Igić & Kohn, 2001). Roles of the non-S-RNase genes in this class are poorly understood, if at all. Based solely on the high diversity of primary sequence features, and expression patterns, they appear potentially highly functionally disparate.

Estimates of ancestor-descendant relationships among the three classes are challenging without additional information, because of the high sequence divergence and uncertainty over the prior expectation for rooting lineage(s). In the analyses using nucleotide alignments and models of sequence evolution, plausible outgroup sequences from algae disrupt the monophyly of land plant RNases, very possibly as an artifact of deep divergence (over 450 My), at the limits of inference for a relatively short gene (ca. 600 bp). Two sequences from *Volvox carteri*, and one from *Chlamydomonas reinhardtii* form a clade that does not include the sequence from *Bryopsis maxima*. Rooting the trees using the *Volvox*/*Chlamydomonas* clade instead results in monophyly of classes II and III, while rooting the tree using the *Bryopsis* sequence results in monophyly of classes I and III. Representative genes from algae are monophyletic in the trees produced using amino acid model of sequence evolution (AA trees). Placing the root between the algal clade and land plant RNases, class II sequences are sister to classes I and III. None of the three possible arrangements among the classes receives significant support. The most apparent incongruence found in all RNase trees—independent of reconstruction method used—is the placement of class I lycophyte, fern, and gymnosperm sequences within the angiosperm RNase clades (Fig. S1d). However, the posterior support values for this arrangement vary wildly (0.01 and 0.84 for best-scoring maximum likelihood trees inferred using amino acid and nucleotide data, respectively). Additionally, in analyses with nucleotide data, gymnosperm class II genes are placed within monocot sequences with posterior support of 0.69 (Fig. S1a).

We identified a set of 91 sequences either corresponding to proteins established to be catalytically inactive in functional studies (MacIntosh et al., 2010; Ohkama-Ohtsu et al., 2004; Wei et al., 2006; Gausing, 2000; Van Damme et al., 2000; Kim et al., 2004), or lacking a conserved histidine residue that is essential for ribonuclease activity (Jost et al., 1991; Taylor et al., 1993; Irie, 1999).

**Figure 2:**
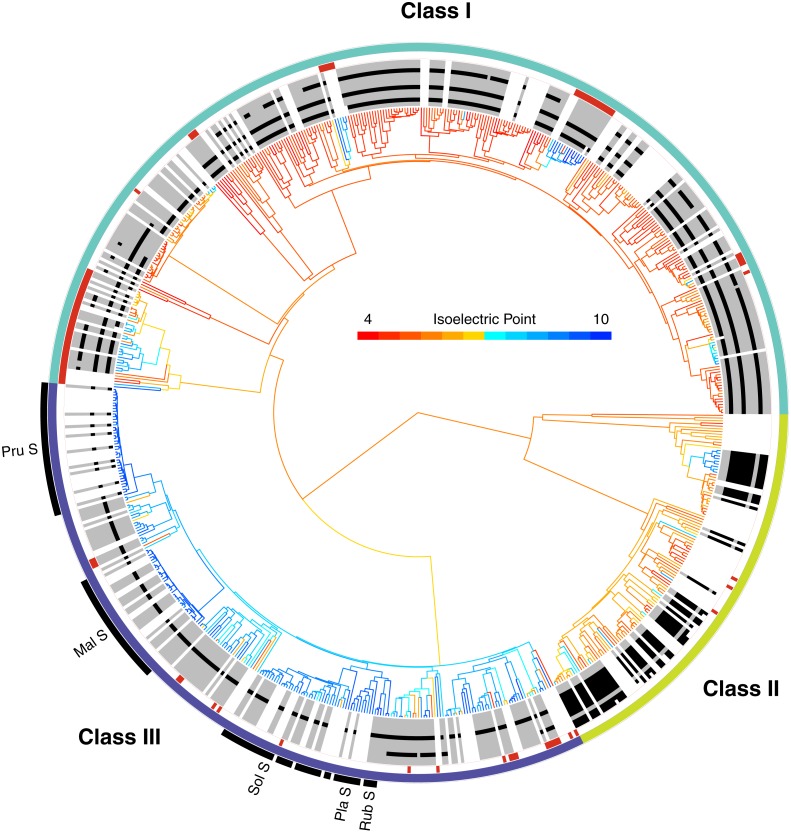
The phylogenetic distribution of intron patterns, isoelectric point (pI) values, and RNase function of the T2/S-RNase family in land plants. Class designations are labeled (I, II, & III) along with key groups known to function as S-RNases (Pru, *Prunus*; Mal, *Malus*; Sol, Solanaceae; Pla, Plantaginaceae; Rub, Rubiaceae). Red notches show sequences known to lack RNase function (or inferred to lack the function based on absence of a histidine essential for that function). The greyscale ring illustrates the pattern of intron presence (black) or absence (grey) at eleven positionally homologous introns found in T2/S-RNases in land plants, clarified in Fig. 3. White areas indicate that the sequence was too short to infer presence–absence, or entirely unavailable (cDNA sequence). The tree branches are colored by the predicted pI value (scale shown) of the amino acid sequence, which was reconstructed for internal branches.

The placement of these sequences is shown in Fig. 2. They are distributed across the tree, clustering in several small clades, often associated with changes in pI value (see below). In our dataset, class I contains 67 such inferred non-functional sequences, scattered across nine clades, class II contains three sequences in three clades, and class III contains 21 sequences in 12 clades (Fig. 2). The pattern is consistent with independent losses of ribonuclease function, perhaps following gene duplications or losses of SI.

### Intron positions

**Figure 3:**
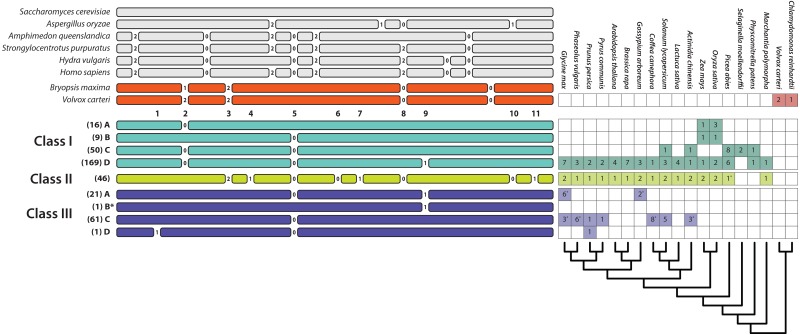
Relative positions of introns in the T2/S-type RNases of the three classes of land plants, algae, and several fungal and animal species and their numbers in some of the sequenced genomes (a star next to a number indicates that the sequence was incomplete or the genome was in the early stages of assembly and the actual paralog count may be lower). Numbers listed below algal gene structures represent 11 intron positions present in land plants. Numbers between exons represent intron phases. Numbers in parentheses represent the number of sequences with the specific intron patten in our dataset. Pattern III-B^*^ contains a single intron at position 9. This pattern has no EST support and was predicted based on genomic sequences from *Fragaria nubicola* and *Fragaria vesca*.

Apart from several scattered and easily identifiable recent gains and losses, the patterns of intron presence and absence are well-conserved and remarkably concordant with our T2/S-RNase gene trees (Fig. 2). We identified 11 observed intron positions across all land plants (embryophytes) examined. The position, phase (reading frame), and numerical abundance of introns are summarized in Fig. 3. Introns at a given position, which we supposed to share ancestry based on extremely similar position in sequence alignment (within seven nucleotides in final alignment), did not exhibit any phase variation across land plants. Such conservation of exon-intron boundary positions within codon triplets reinforces our classification of intron occurrences. We also noted the phylogenetic distribution and number of genes matching each intron pattern in species with complete genomes (Fig. 3).

Intron patterns are largely concordant with the phylogenetic classes. Class I genes display four intron presence–absence patterns. Most sequences contain three introns at positions 2, 5, and 9 (pattern I-D). Absence of intron at position 9 defines the second most frequent pattern, I-C. Two additional single intron patterns, I-A and I-B, containing introns at positions 2 and 5, respectively, occur only in Poaceae (grass family). Class II sequences are remarkable, in that all 46 sequences examined to date contain eight introns, and exhibit no apparent variation in intron pattern. Most class III sequences contain a single intron at position 5 (pattern III-C). Almost all known S-RNases exhibit this intron pattern although *Prunus* S-RNases contain an additional intron at position 1 (pattern III-D). Pattern III-A (intron positions 5 and 9) is found in the sequences of several distantly related Rosid species (*Ricinus communis*, *Carica papaya*, *Cajanus cajan*, *Glycine max*, *Theobroma cacao*, *Gossypium arboreum*, *Gossypium raimondii*). Although the dataset contained 21 sequences with this intron pattern, most of these were paralogs. Eleven copies were found in the genome of *Theobroma cacao*, and other species contained between one and three copies. Pattern III-B^*^ contains a single intron at position 9. This pattern has no EST support and was predicted based on genomic sequences from *Fragaria nubicola* and *Fragaria vesca*.

Intron positions and their phases are highly conserved. Position 5 is found in all three plant T2/S-RNase classes, as expected (Igić & Kohn, 2001). Position 9 introns are shared by some class I and III members. All other intron positions are class-specific. Position 2 occurs only in class I sequences and seven intron positions (3, 4, 6, 7, 8, 10, and 11) are unique to class II. Overall, merely eight distinct intron position patterns exist, seven of which are class-specific. One apparent exception—likely due to convergence—is the shared pattern of a single intron at position 5 in nine grass sequences belonging to class I and many sequences, especially S-RNases, in class III (patterns I–B and III–C).

None of the intron patterns found in land plants appear located in identical positions as those in algal, fungal, or animal genes. Sequences from the *Volvox carteri* and *Bryopsis maxima* contain four introns, three of which may be in the same ancestrally shared positions as the ones found in land plant sequences. Although their phases differ, and exact location appears to be slightly shifted, each species contains an intron possibly ancestrally shared at position 2 in plant T2/S-RNases. Introns in the second reading phase (+2)—a potential homologue to one found at position 3 in land plants—is also present, although our sequence alignment is ambiguous in this region. Another intron appears to be homologous to position 8. It is in the same phase (+0 phase; not interrupting the reading frame) as the ones found in land plant T2/S-RNases.

A limited sample of animal sequences examined contain six to eight introns, two of which are potentially in identical sites to those we find in the land plants. One of these, near position 5, as in plants, is in phase +0. The other, near position 8, is in phase +2, while plant introns at this position are in phase +0. Two out of four introns found in fungal sequences may be homologs of the ones found in plant sequences. An intron from *Aspergillus oryzae* is near position 8 and is in the same phase (+0) as land plant introns. The other intron from this species, near position 10, is in phase +1, compared to phase +0 of plants. It is unclear how quickly intron sliding and phase evolve, generally, and we have little statistical evidence to establish clear links between fungal, animal, and plant RNases in our dataset.

### Isoelectric point (pI) values

**Figure 4:**
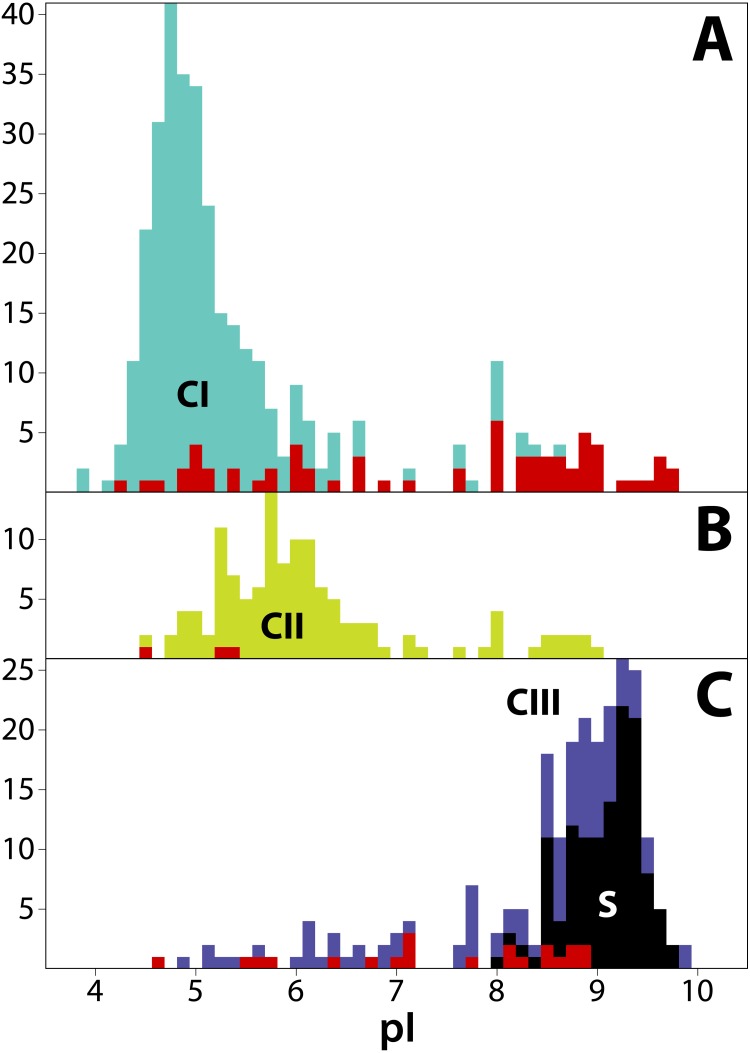
Frequency distribution of isoelectric points (pI) for 711 T2/S-RNases in land plants, separated by phylogenetic group (‘class’). Classes are consistently colored as before. (A) Class I (green, *n* = 349). (B) Class II (yellow, *n* = 125). (C) Class III (purple, *n* = 237). Red bars indicate sequences known to lack RNase function (or inferred to lack the function based on absence of a histidine essential for that function). Black bars represent known S-RNase sequences. Note that functional S-RNases rarely display pI < 8.0, which is otherwise common in T2/S-RNases.

We estimated isoelectric point (pI) values for T2/S-RNases as vague heuristic indicators of possible subcellular localization and function, with a possibly informative pattern of values across the gene family tree (Drawid & Gerstein, 2000; Kirkwood et al., 2015). The predicted pI values in our dataset range from 3.91 to 9.91. Although the predicted pI value ranges of all three RNase classes largely overlap, the values show distinct class-specific trends (Fig. 4). Class I peptides generally have acidic pI values, with a median of 5.04 (min = 3.91; max = 9.75; *n* = 349). Class II peptides have similarly acidic pI values, with a median of 5.90 (min = 4.52; max = 9.03; *n* = 125). By way of contrast, class III peptides are significantly more basic. The non-S-RNases (or unknown function peptides) have a median pI of 8.56 (min = 4.61; max = 9.91; *n* = 118), while S-RNases have a median pI of 9.18 (min = 8.10; max = 9.73; *n* = 119).

In order to examine the distribution of pI values across T2/S-RNases, we mapped the predicted sequence pI values on the corresponding gene tree (Fig. 2). Class I contains nine independent pI shifts from acidic to basic values, seven of these were associated with the loss of the active histidine residue. Class II contains eleven independent pI shifts from acidic to basic values, none of these were associated with the loss of the active histidine residue. Class III contains 22 independent pI shifts from basic to acidic values, six of these were associated with the loss of the active histidine residue. The apparent conservation and concordance of pI is fairly remarkable, given its lack of clear relationship with protein function (Drawid & Gerstein, 2000; Brett, Donowitz & Rao, 2006).

### F-box domain-containing genes near T2/S-RNase loci

We searched the available genome assemblies for the newly identified class III T2/S-RNase family members, without a known function, in an attempt to find whether they are co-located with F-box motif-containing genes (within 2 Mb). We reasoned that such associations may have comprised—or still comprise—a functional S-locus. But the resulting picture is complex. Many class I and class II RNases also contain F-box motif-containing genes within 2 Mb, which is perhaps unsurprising given their abundance in plant genomes (Wang et al., 2004). Nevertheless, the structure of putative S-loci ought to resemble the canonical pattern: a class III T2/S-RNase, accompanied by a more than a few F-box-containing genes.

**Figure 5:**
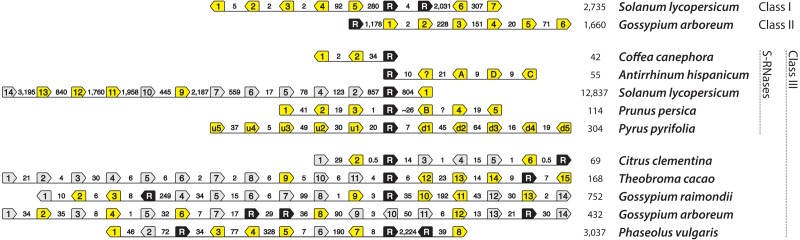
T2/S-type RNases and nearby F-box-containing genes (shortened to ‘F-boxes’). Pentagonal boxes illustrate genes, with points indicating transcriptional direction. Black polygons, labeled with “R” represent RNase gene locations. Grey and yellow polygons represent F-box locations. Grey background indicates that in frame stop codon is present in the F-box sequence. Numbers between the genes indicate distances between them (in kb). The final column of numbers indicates the total length of the illustrated available genomic segment. The first two rows show haplotypes that contain non-S-RNases from classes I and II, in tomato and cotton, respectively. Ten bottom haplotypes each contain RNases belonging to class III. Five of these are known self-incompatibility haplotypes: *Coffea canephora* CA4b, *Antirrhinum hispanicum* S2, *Solanum lycopersicum* S20 (relic), *Prunus persica* S2, *Pyrus pyrifolia* S2. Five additional haplotypes contain class III RNases, and resemble known S-haplotypes. All of the ones shown here, display two copies of RNases at the putative S-haplotype. However, F-boxes in these haplotypes cluster monophyletically with known S-locus F-boxes. It is not known how accurately the haplotypes are assembled, given the likely difficulties for automated assembly represented by S-loci. Therefore, haplotypes from these species, and especially their self-incompatible relatives, are excellent candidates for further study.

We identified genomic regions that contain a class III RNase and multiple F-box loci in five eurosid species, not including previously characterized S-loci (Fig. 5 and Table S2). *Citrus clementina* (Rutaceae) genome contains a class III RNase and six F-box loci on scaffold 5. This genomic region, however, was largely unresolved (46% composed of ambiguous characters), which may have prevented the discovery of more F-box loci. The genomes of *Theobroma cacao*, *Gossypium raimondii*, and *Gossypium arboreum* (Malvaceae) contain a class III RNase and 15, 14, and 14 F-box loci on scaffold 10r, chromosome 11, and chromosome 10, respectively. *Phaseolus vulgaris* (Fabaceae) contains a class III RNase and eight F-box loci on chromosome 4, as in *C. clementina*, however, 45% of the genomic region investigated was unresolved. Two or more F-box loci in each of these haplotypes contain in-frame stop codons, which is also the case in the non-functional haplotype of domesticated tomato, *Solanum lycopersicum* (Table S2). Unlike canonical S-haplotypes, however, all of these contain more than one class III RNase; *Citrus clementina*, *Theobroma cacao*, and *Gossypium raimondii* contain two, while *Gossypium arboreum* and *Phaseolus vulgaris* contain three.

We also examined evidence for an alternative cause of co-location of RNases and F-boxes, an unspecified functional constraint that causes an association of RNases and F-boxes. Interestingly, as is the case in *Arabidopsis thaliana* (Wang et al., 2004), genomic regions flanking class I and II T2/S-RNases from several species also contain multiple F-box loci (bottom two haplotypes in Fig. 5). Genomic segments (2 Mb) flanking the well-characterized *Solanum lycopersicum* RNases LX and LE (class I T2/S-RNases), which occur in tandem on chromosome 5, contain at least seven F-box loci. Similarly, *Gossypium arboreum* class II locus on chromosome 4 has at least six F-box loci. Specifically, all F-box sequences flanking class III RNases in Fig. 5 cluster within F-box groups that contain known S-locus F-box genes (Fig. S3), while the F-box genes associated with *Solanum lycopersicum* class I and *Gossypium arboreum* class II RNases cluster outside this clade.

## Discussion

Our analyses predict that the RNase-based self-incompatibility system is increasingly unlikely to be found outside of eudicots, while its undiscovered presence in other families within the core eudicots (The Angiosperm Phylogeny Group, 2016) is nearly certain. To this end, we demonstrate that it is possible to find putative S-locus relicts, even in crude genome assemblies of non-model systems. Second, we examine the evidence in support of common ancestry and divergence of S-RNases, the female component of self-incompatibility. An alternative view posits wholesale convergence, but we generally find it tends to be based on a strict interpretation of analyses stemming from flawed conceptions of homology and statistical phylogenetic models. It underestimates the possible mechanistic divergence at such a vast scale of elapsed time. More broadly, our current understanding suffers from the exclusive focus on a handful of distantly related model systems. Some of these may be highly derived—modified with respect to the system found in the most recent common ancestor—and idiosyncratic. We reason that our analyses place the majority of the weight of evidence on common ancestry, but we argue that renewed aim at discovery of the molecular basis of SI in distantly related families may prove necessary to settle lingering doubts.

### T2/S-RNases in land plants

T2/S-RNases comprise a ubiquitous family of endoribonucleases, often found in low copy number in the genomes across all domains of life, with the sole exception of Archaea (Irie, 1999; MacIntosh, 2011). Their wide distribution alone suggests conservation of important function(s). A common and possibly ancestral role appears to be ribosomal RNA decay and recycling (Hillwig et al., 2009; Ambrosio et al., 2014). In a variety of species, T2/S-RNases are induced under oxidative stress and function in tRNA cleavage, which appears to be a conserved response in eukaryotes (Thompson et al., 2008; MacIntosh et al., 2001; Ambrosio et al., 2014). In plants, their main function appears to be phosphate harvesting from degraded RNA (MacIntosh, 2011). Indeed, phosphorus is a limiting nutrient for plants, so intracellularly abundant rRNA and senescing tissues comprise important recyclable resources (Bariola et al., 1994).

We find that seed plant genomes feature an expanded repertoire of T2/S-RNases, compared with other organisms, so that each diploid genome contains four or more members of this gene family. The causes of this expansion are difficult to infer, but there is an intriguing possibility that it accompanied the invasion of land and the subsequent development of vasculature and increase in stature, especially as plants moved away from steady sources of dissolved available inorganic phosphates. It is inviting to further speculate that unicellular plants are unlikely to have many members of the T2/S-RNases. Multicellular plants, especially those with a variety of metamers and displaying diverse organ identity, may rely on separate subfunctionalized paralogs for efficient phosphate recycling and recruitment. They would also be far more prone to diversification into neofunctionalized paralogs with unrelated roles, such as sexual self-recognition and defense. The exact distribution and patterns of diversification of T2/S-RNases in land plants remain unclear, but increasing genome sequencing coverage across plants is likely to help focus the study on the processes than may have been responsible for shaping the many roles taken on by this gene family.

#### Three diverse ‘Classes’ of T2/S-RNases

Three distinct phylogenetic groups of T2/S-RNases are well-supported in land plants (Fig. 1 and Fig. S1; Igić & Kohn, 2001). The inference of relationships within and between classes is backed by the congruence between gene genealogies and species phylogeny, an apparently non-random association with their intron distributions, isoelectric point (pI) values, and also some functional data (Table S3). Class I and II genes have a wide taxonomic distribution, spanning land plants, while class III genes are only found in core eudicots.

The majority of diploid genomes examined contain at least two members of class I (Fig. 3), which may indicate that it is comprised of two commonly combined cryptic classes (we retain the naming convention simply to avoid further confusion). Most class I members have three introns, and this appears to be their ancestral state, although the intron at position 9 (Fig. 3), seems to have been repeatedly lost, or it may ‘flicker’ due to an unaccounted-for process, such as recombination. Class I genes perform a variety of functions, including response to phosphate starvation, senescence, as well as defense, and they are both secreted and expressed in a number of organelles (reviewed in MacIntosh, 2011). Some are, for example, found in digestive fluids of carnivorous plants (Okabe et al., 2005; Nishimura et al., 2013; Nishimura et al., 2014). Another hint about their diverse roles is exemplified by the confidently placed 37-sequence clade of genes from the grass family (Poaceae), whose members lack one or both conserved histidine residues essential for endoribonuclease activity (MacIntosh et al., 2010). In addition, thirty other class I sequences in our dataset lack these conserved histidine residues, notably a 14-sequence clade comprised of genes from plants in the Caryophyllales, although virtually nothing is known about their expression or function. In each cases, the loss of histidines essential in catalytic RNase function is associated with a shift to basic isoelectric point values, and may therefore signal a functional shift (instead of pseudogenization).

Class II members are nearly as widespread as class I, with a notable discontinuity. While they were found in each seed plant genome we examined, they appear absent from mosses and hornworts, but present in a distantly related *Marchantia*. Genes within class II T2/S-RNases stand out in low divergences, as well as their highly conserved eight-intron structure. We find a single copy in most genome assemblies, with the exception of recently duplicated genomes, which contain two copies. Three class II paralogs do not have the canonically conserved histidine residue, required for RNA catalysis. However, a possible function of only one such gene, *Calystegia sepium* CalsepRRP, has been investigated, and it appears to function in protein storage (Van Damme et al., 2000). Their function is broadly characterized in a number of plants, where they generally function in ribosomal RNA recycling throughout the life of a cell, and are constitutively expressed, although some show increased expression during senescence (Taylor et al., 1993; Hillwig et al., 2011; Kurata et al., 2002; Köthke & Köck, 2011; Liang et al., 2002). A class II member from *Arabidopsis thaliana* (RNS2) is required for ribosomal RNA decay in this species. This role, mirrored by RNASET2 in humans and zebrafish, as well as Rny1 in yeast, makes it appear as a possible ancestral functional homolog across eukaryotic T2/S-RNases. Moreover, *Arabidopsis thaliana* RNS2 knock-outs cause possibly lethal (environment-dependent) phenotypes, which are not rescued by the presence of four class I genes (Hillwig et al., 2011). Neither of these two observations are, however, sufficient to demonstrate ancestry of class II T2/S-RNases RNases in plants. It is broadly understood that functional roles can change rapidly, especially in the presence of paralogs, so that data from one species (for example, *Arabidopsis thaliana*) has limited implications for the remaining ca. 400,000 species which diverged around 500 million years ago. A great deal of evidence hinges on class II genes being absent from mosses and hornworts, even after more and better genomes are assembled. If they are not found, class II genes may not be ancestral or essential (independent of the environmental context).

Class III genes, which include the S-RNases, are restricted to the core eudicots. With few exceptions, they are highly divergent and show intron presence/absence patterns similar to class I members. It appears certain that many class III members have a range of functions unrelated to self-incompatibility. For instance, *Petunia* ×* hybrida Phy3* and *Phy4* are expressed exclusively in flowers, and their products are thought to have an antimicrobial role in nectar (Hillwig et al., 2010). *Panax ginseng* GMP is vegetative storage protein with no ribonuclease activity although it does contain the catalytically active histidine residue (Kim et al., 2004). *Pisum sativum* P43 binds DNA polymerase in chloroplasts and stimulates its activity (Gaikwad et al., 1999).

The presence of the canonical class I & II sequences in liverworts shows that both groups were likely already established in the earliest land plants. This pattern of distribution indicates that class II genes may not represent orthologs of animal and fungal T2/S-RNases, and may not be essential, as they are possibly missing from some land plants. A second line of evidence in this vein is that the conserved functions of rRNA regulation, phosphate-harvesting, and scavenging, are attributed to enzymes in both classes I and II. Our findings show that the T2/S-RNase family is remarkable in its evolutionary lability, and it does not enable us to conclusively identify eukaryotic orthologs and precise ancestral-descendant ordering.

#### Congruence with intron position and pI

A unique challenge faced in the analyses of a gene family evolution, as opposed to the inference of species trees, is that we do not target the loci used for inference in a manner that reduces a variety of challenges (discussed below) and maximize resolution. Thus, it is advantageous to obtain independent lines of evidence to aid in assessment of the recovered relationships. In this vein, we mapped intron presence/absence data and pI values on the T2/S-RNase gene tree to examine the congruence of their distribution with the recovered topology (Fig. 2). The evolution of intron presence and position in T2/S-RNase family is fairly dynamic (Igić & Kohn, 2001). It appears highly unlikely, however, that a lost intron would be regained at exactly the same position, and in identical phase. Similarly, although these features do not provide enough resolution to evaluate the gene tree topology in detail, the three major RNase classes show distinct intron presence/absence patterns and pI value trends. The existence of shared structural features of known S-RNases, basic pI value and an intron at position 5 but not 9 (as well as other features, Vieira, Fonseca & Vieira, 2008), does not by itself guarantee orthology, but it may be a useful heuristic used in searches for new S-RNases.

The functional causes of association of protein pI values are unclear (Drawid & Gerstein, 2000; Brett, Donowitz & Rao, 2006), but pI appears to be a significant correlate of S-RNase function (Fig. 4 and Fig. S2). Isoelectric point is thought to partly determine protein stability and solubility by modulating relative protein-water, protein-protein, protein-membrane, and other interactions (Kirkwood et al., 2015). It is also generally associated with subcellular location (Drawid & Gerstein, 2000; Ho, Hayen & Wilkins, 2006). We use it here as a rough whole-sequence point estimate proxy for a vaguely defined functional aspect of proteins. Specifically, we suspect that strong local departures in pI of closely related clades of proteins inform us about possible departures in function or expression from the rest of the clade. It is of some interest that repeated losses of ribonucleic function result in rapid shifts in pI. This strongly suggests that, whatever its role, the maintenance of a particular pI range is related to protein function. And yet it, too, tells us very little. In many instances, non-S-RNases display pI values well within the basic range of S-RNases, despite the commonly-held view that they generally show preference for acidic pH.

#### Conservation of haplotype structure

We were particularly interested in the co-occurrence of tandem-replicated copies SLF/SFB genes, in the vicinity of T2/S-RNases. Published genomes of *Citrus clementina*, *Theobroma cacao*, *Gossypium raimondii*, *Gossypium arboreum*, and *Phaseolus vulgaris* contain regions with RNases and SLFs/SFBs matching these criteria, and may present clues that S-RNase-based SI is widespread in the core eudicots. Many of the species sequenced to date are cultivated varieties, self-compatible, and possibly specifically selected for loss of SI function, which is reflected in their apparently decayed S-loci. For example, the S-haplotype of the domesticated tomato, *Solanum lycopersicum*, which lost SI function millions of years ago, retains a set of pseudogenized F-box loci, spread over 18 Mb of tightly linked subcentromeric region (Kubo et al., 2015). Therefore, it is possible that other such losses of S-RNase based SI may be preserved in the genomes of self-fertilizing species. This observation may seem particularly encouraging in efforts to find S-loci in new, non-model systems. One complicating factor is the the presence of hundreds of copies of F-box-containing genes in many angiosperm species (Yang et al., 2008; Hua et al., 2011). We find that clusters of F-boxes sometimes surround class I and class II RNases, as well. An intriguing explanation may posit that the T2/S-type RNase association with F-box proteins predates the evolution of S-RNases. On the other hand, for example, the *Arabidopsis thaliana* genome contains around 660 F-box loci (Yang et al., 2008; Hua et al., 2011), and this association is particularly likely to be spurious. The data on patterns and processes that govern the distribution of F-box genes is limited, and it is not prudent or possible to derive a generally valid probability of co-location of RNases and F-boxes at this time. Accumulating data from complete genomes should enable tests in the near future. In any case, functional S-loci are expected to lack the recombination rates necessary to break their salient feature—extensive linkage disequilibrium.

It is increasingly clear that the RNase gene trees (e.g., Richman, Broothaerts & Kohn, 1997; Igić & Kohn, 2001; Steinbachs & Holsinger, 2002) alone are insufficient to generate clear expectations about a number of long-standing questions regarding evolutionary history of S-RNase-based SI. We used genomic features to identify what may be partly preserved relictual S-loci in genomes of plants with unknown mechanisms of SI, or wholly lacking SI. Candidates that may have expressed S-RNase-based SI ancestrally can be found in this way, but await discovery and functional studies in taxa that express SI. Nevertheless, existing genome assemblies present new challenges, including a relatively narrow taxon sample, not aimed at SI species, and the technical challenges that possibly yield poor assemblies in the region housing the S-locus. The finding of possible “molecular fossils”, in the form of relict S-loci, enables a somewhat informed speculation about the distribution of this mechanism, and outlines clear forward procedures to establish the history of self-incompatibility in plants.

### Implications for the evolution of self-incompatibility

The distribution of both the S-RNase-based SI and class III RNases has so far been restricted within the core eudicots. Combined with the shared intron presence–absence patterns found in S-RNases and similarities in the male components of SI, this provides considerable evidence for the single origin of S-RNase-based SI. Class III sequences are not found in all core eudicots, and do not appear to be strictly essential in any organism in which they were studied to date.

It is perhaps significant that they are absent from several sequenced species in the genera *Arabidopsis* and *Brassica*, which both express a sporophytic SI system (or are otherwise self-fertile). This observation suggests that class III RNases originated before the divergence of core eudicots, and may be maintained due to their role in RNase-based SI. It is possible then, that novel functions of proteins found within class III originated from S-RNase paralogs, with current utility unrelated to sexual systems. Another possibility has gained traction.

Starting with Sassa et al. (1996), many have proposed that S-RNase-based SI in Rosids and Asterids evolved independently, based on the non-monophyly (polyphyly) of S-RNases, although their conclusions contrasted with two contemporary analyses (Xue et al., 1996; Richman, Broothaerts & Kohn, 1997). This argument is consistent with repeated recruitment of class III T2/S-RNases for a role in SI. Later work involved a steadily increasing number of sequenced genes, and clarified that strict monophyly of S-RNases is not a necessary condition for shared ancestry (e.g., Steinbachs & Holsinger, 2002). For example, gene duplication, followed by functional changes, could easily account for the widespread occurrence of paralogs, and yield non-monophyly of functional S-RNases, as could a great variety of sources of error in phylogenetic inference. Moreover, subsequent studies also found that the male component of SI response in this system, SFBs, are expressed in the pollen of species from each of the well-studied families (Zhou et al., 2003; Ushijima et al., 2003; Sijacic et al., 2004), a development that appeared to affirm the single-origin hypothesis beyond doubt. The debate has now acquired a new twist.

It has come to light that an increasing number of differences distinguish the mechanism of action of S-RNase-based SI in *Prunus* (Rosaceae) from that acting in the relatively closely related subtribe Malinae (Rosaceae) and in the euasterid families Solanaceae and Plantaginaceae. The inferred dissimilarities include the mode of recognition (self- vs. non-self; e.g., Ushijima et al., 2004; Fujii, Kubo & Takayama, 2016), phenomenology and causes of breakdown of SI (Golz et al., 2001; Ushijima et al., 2004; Hauck et al., 2006; Xue et al., 2009), magnitude of selection and sites experiencing it (Ashkani & Rees, 2016), S-locus structure (Akagi et al., 2016), as well as the patterns of divergence and relationships (Kohn, 2008; Akagi et al., 2016) among both S-RNases and F-box-containing genes (SLF/SFB). A range of novel evolutionary scenarios have been proposed to explain them, ranging from surprising wholesale convergence to homology with divergence (Morimoto, Akagi & Tao, 2015; Aguiar et al., 2015a; Akagi et al., 2016).

On the other hand, it seems particularly likely that precise models describing the evolution of RNase-based SI remain elusive in part due to its apparent variation and complexity. The problem is compounded by a notable lack of detailed functional studies outside of a few species in Solanaceae, Plantaginaceae, and Rosaceae, which ensures that we have very little ability to shape reasonable expectations regarding the capacity of such systems to undergo the kinds of divergence observed over the relevant timescales.

Even the most sophisticated evolutionary analyses can merely supply a scorecard of similarities and differences in the discovered components of RNase-based SI, in the absence of context provided by extensive comparative data. The question is somewhat academic, in the sense that there are well-documented examples of the incredible capacity for change among molecular components underlying conserved traits. It is possible to imagine a total turnover of genetic components, which would eliminate many outward diagnostic signs of homology. This process is clearly exemplified by the repeated independent co-option of unrelated genes as lens crystallins in different vertebrate lineages (True & Carroll, 2002). Replacement or modification of one or more genetic components of a complex system by co-option of unrelated genes does not necessarily interrupt its genealogical continuity and function. Therefore, just as we discarded the expectation of strict monophyly among the molecular components of SI, due to the vast capacity of genes to undergo duplication and subsequent changes, perhaps we should do the same with the expectation of identity for all functional details of SI response for all lineages. It seems that the main difficulty concerns the development of a common framework of approaches that can delineate ‘deep homology’ (Shubin, Tabin & Carroll, 1997) from convergence, comprised of re-recruitment of similar components.

The presently employed framework or haphazard data collection—and often flawed analyses—from a number of unrelated lineages is insufficient for detailing this instance of deep homology. The growing list of differences and interacting units that cause RNase-based SI, each with possible unknown pleiotropic effects, is increasing the complexity of our task. In Brassicaceae, where a distinct kinase-based mechanism operates, we are aware of one complicating instance within the family. Species in the genus *Leavenworthia* appear to contain two paralogous loci, one of which apparently encodes S-allele phenotypes, and another with an as yet undetermined function (Chantha et al., 2013). As the authors point out, such a finding illustrates the vastness of the problems before us, but it does not necessitate rejection of homologous ancestry of the trait across the family. In the present study, with perhaps the most extensive collection of data on female and male components of RNase-based SI at hand (to date), we likewise find that there is little evidence to overturn the long-standing hypothesis of a single RNase-based GSI system origination predating the common ancestor of rosid and asterid eudicots. This conclusion, like those of its detractors, is necessarily rife with potential problems.

### Challenges for the inference of history of T2/S-RNases and self-incompatibility

Phylogenetic analyses of the components that interact to affect SI responses are vulnerable to a number of sources of error and bias (Felsenstein, 2004). Most studies that posit some manner of convergent re-evolution of RNase-based SI principally rely on the precise relationships of S-RNase (or SLF/SFB) gene trees. It is trivially unsurprising that a particular group of functional S-RNases with a shared history may be recovered as para- or polyphyletic, even under perfectly specified inference models. Sequence-based inferences of evolutionary relationships among very distantly related genes should be viewed with some skepticism. In the particular case of the T2/S-RNase family in angiosperms, our focus here, the available gene sequences are relatively short and highly divergent, so that significant loss of information is expected due to the accumulation of multiple changes per site, even under correctly specified models of sequence evolution. Under such conditions there may simply not be enough information to accurately recover the historical relationships. This uncertainty may not be reflected in node support values due to methodological artifacts, such as long branch attraction—a spurious, yet confident, association of distantly related sequences.

More seriously, inference bias and error can result from a vast range of unspecified evolutionary processes, such as gene duplication, large (multi-nucleotide) subsequent changes or loss. Phylogenetic tree inference is conditional on both the correct data (sequences and their alignment) and the model of sequence evolution. While we may be somewhat confident that the gene sequences are adequate, the assessment of alignment accuracy is less trivial, especially with high sequence divergence (Felsenstein, 2004; Kumar et al., 2012). Substitution models encompass only a narrow range of biologically possible processes, and they do not easily accommodate indels, recombination, variation of substitution rates over time (heterotachy) and between clades (Kumar et al., 2012). Perhaps critically for analyses involving the numerous SLF/SFB paralogs, the presently used models of tree inference do not accommodate gene conversion, which can result in spectacular model-misspecification, and subsequently erroneous inference. Such models exist (Song et al., 2011), but are not easily integrated into the common workflows. Despite these difficulties, molecular phylogenetic approaches remain indispensable, and are often the only hypothesis generating tools available. The task of reconstructing evolutionary events on timescales of ca. 50–100 My ought to be daunting, carefully framed, and generally include circumspect qualification of the resulting analyses.

Nevertheless, even in the presence of a variety of flaws, evaluation of a variety of protein features in phylogenetic context may help to narrow down the list of candidate T2/S-RNases for functional characterization. In this vein we provide an on-line service (http://t2.karol.is), which places user-provided sequences on the T2/S-RNase phylogeny used in this study. If amino acid sequences are provided, pI values will be calculated. Users of such automated workflows would do best not to ignore a variety of possible problems, as outlined above.

## Conclusions

The number of sequenced T2/S-RNase variants continues to grow dramatically, principally as a byproduct of sequencing projects. Haphazard collection of data offers glances into this enigmatic protein family, but the great expansion of sequence number has not qualitatively improved our understanding of their evolutionary history and function. The accumulating pile of sequences is, however, becoming proportionately cumbersome, and it demands caution, given that many assemblies are often automatically generated with little or no validation.

We surmise that a great deal of circumstantial evidence, especially the identity of male and female component genes, phylogenetic relationships among them, and other comparative patterns concerning physical and functional features—still best support a single ancestral origin of S-RNase-based SI, followed by rampant losses of SI, as well as transitions to wholly new molecular mechanisms of SI. While the system is presently found across distantly related core eudicots, detailed functional studies are usually performed in a select few species across this yawning divide. Specifically, Rosaceae, Solanaceae, and Plantaginaceae receive a disproportionate amount of attention, largely due to inertia and their economic value. Detailed functional data from additional groups is sorely needed as a comparative metric of expectations for functional divergences.

Clarification on the status and extent of homology is lacking partly because discovery of the system in new families, of enormous value for comparative work, has slowed. Since 1992, the only family with a newly characterized S-RNase-based system is Rubiaceae (Asquini et al., 2011; Nowak et al., 2011), although additional efforts were made, at least in species of Campanulaceae and cultivated species of Fabaceae (Good-Avila et al., 2008; Aguiar et al., 2015b). Virtually all recent reviews of the distribution of SI lament the lack of discovery of genes underlying this phenomenon in the remaining 99% of angiosperm families, which could clarify the distribution of S-RNase-based systems and relationships among them, as well as shape our expectations regarding the evolutionary history of all SI systems, many of which are not yet characterized (Allen & Hiscock, 2008; Igić, Lande & Kohn, 2008; Gibbs, 2014).

## Supplemental Information

10.7717/peerj.3790/supp-1 Supplemental Information 1 Supplemental information A list of ENTREZ search terms used to obtain T2/S-RNase sequences from GenBank. List of species removed from the dataset. Gene tree of T2/S-RNases in land plants with tip names and other annotations. The relationship between calculated pI values and experimental pH optima for several T2/S-RNases in land plants. Gene tree of F-box-containing genes near T2/S-RNase loci. Table of GenBank accessions used. Table of the referenced functional T2/S-RNase studies. Table of genomes used.

## Additional Information and Declarations

### Data Availability

The following information was supplied regarding data availability: Ramanauskas K, Igić B (2017) Data from: the evolutionary history of plant T2/S-type ribonucleases. Dryad Digital Repository. http://dx.doi.org/10.5061/dryad.8hv0k.

## References

[ref-1] Aguiar B, Vieira J, Cunha AE, Fonseca NA, Iezzoni AF, Van Nocker S, Vieira CP (2015a). Convergent evolution at the gametophytic self-incompatibility system in *Malus* and *Prunus*. PLOS ONE.

[ref-2] Aguiar B, Vieira J, Cunha AE, Vieira CP (2015b). No evidence for Fabaceae gametophytic self-incompatibility being determined by Rosaceae, Solanaceae, and Plantaginaceae S-RNase lineage genes. BMC Plant Biology.

[ref-3] Akagi T, Henry IM, Morimoto T, Tao R (2016). Insights into the *Prunus*-specific S-RNase-based self-incompatibility system from a genome-wide analysis of the evolutionary radiation of S locus-related F-box genes. Plant and Cell Physiology.

[ref-4] Allen AM, Hiscock SJ (2008). Evolution and phylogeny of self-incompatibility systems in angiosperms. Self-incompatibility in flowering plants: evolution, diversity, and mechanisms.

[ref-5] Altschul SF, Gish W, Miller W, Myers EW, Lipman DJ (1990). Basic local alignment search tool. Journal of Molecular Biology.

[ref-6] Ambrosio L, Morriss S, Riaz A, Bailey R, Ding J, MacIntosh GC (2014). Phylogenetic analyses and characterization of RNase X25 from *Drosophila melanogaster* suggest a conserved housekeeping role and additional functions for RNase T2 enzymes in protostomes. PLOS ONE.

[ref-7] Anderson MA, Cornish EC, Mau S-L, Williams EG, Hoggart R, Atkinson A, Bonig I, Grego B, Simpson RJ, Roche PJ, Haley JD, Penschow JD, Niall HD, Tregear GW, Coghlan JP, Crawford RJ, Clarke AE (1986). Cloning of cDNA for a stylar glycoprotein associated with expression of self-incompatibility in *Nicotiana alata*. Nature.

[ref-8] Ashkani J, Rees DJG (2016). A comprehensive study of molecular evolution at the self-incompatibility locus of Rosaceae. Journal of Molecular Evolution.

[ref-9] Asquini E, Gerdol M, Gasperini D, Igić B, Graziosi G, Pallavicini A (2011). S-RNase-like sequences in styles of *Coffea* (Rubiaceae). Evidence for S-RNase based gametophytic self-incompatibility?. Tropical Plant Biology.

[ref-10] Bariola PA, Howard CJ, Taylor CB, Verburg MT, Jaglan VD, Green PJ (1994). The *Arabidopsis* ribonuclease gene *RNS1* is tightly controlled in response to phosphate limitation. The Plant Journal.

[ref-11] Bariola PA, MacIntosh GC, Green PJ (1999). Regulation of S-like ribonuclease levels in *Arabidopsis*. Antisense inhibition of *RNS1* or *RNS2* elevates anthocyanin accumulation. Plant Physiology.

[ref-12] Bjellqvist B, Basse B, Olsen E, Celis JE (1994). Reference points for comparisons of two-dimensional maps of proteins from different human cell types defined in a pH scale where isoelectric points correlate with polypeptide compositions. Electrophoresis.

[ref-13] Bjellqvist B, Hughes GJ, Pasquali C, Paquet N, Ravier F, Sanchez J-C, Frutiger S, Hochstrasser D (1993). The focusing positions of polypeptides in immobilized pH gradients can be predicted from their amino acid sequences. Electrophoresis.

[ref-14] Brett CL, Donowitz M, Rao R (2006). Does the proteome encode organellar pH?. FEBS Letters.

[ref-15] Chantha S-C, Herman AC, Platts AE, Vekemans X, Schoen DJ (2013). Secondary evolution of a self-incompatibility locus in the Brassicaceae genus *Leavenworthia*. PLOS Biology.

[ref-16] Christin P-A, Weinreich DM, Besnard G (2010). Causes and evolutionary significance of genetic convergence. Trends in Genetics.

[ref-17] Cock PJA, Antao T, Chang JT, Chapman BA, Cox CJ, Dalke A, Friedberg I, Hamelryck T, Kauff F, Wilczynski B, De Hoon MJL (2009). Biopython: freely available Python tools for computational molecular biology and bioinformatics. Bioinformatics.

[ref-18] De Nettancourt D (1977). Incompatibility in angiosperms. Monographs on theoretical and applied genetics.

[ref-19] Drawid A, Gerstein M (2000). A Bayesian system integrating expression data with sequence patterns for localizing proteins: comprehensive application to the yeast genome. Journal of Molecular Biology.

[ref-20] Edgar RC (2010). Search and clustering orders of magnitude faster than BLAST. Bioinformatics.

[ref-21] Entani T, Iwano M, Shiba H, Che F-S, Isogai A, Takayama S (2003). Comparative analysis of the self-incompatibility (S-) locus region of *Prunus mume*: identification of a pollen-expressed F-box gene with allelic diversity. Genes to Cells.

[ref-22] Felsenstein J (2004). Inferring phylogenies.

[ref-23] Foote HC, Ride JP, Franklin-Tong VE, Walker EA, Lawrence MJ, Franklin FC (1994). Cloning and expression of a distinctive class of self-incompatibility (S) gene from *Papaver rhoeas* L. Proceedings of the National Academy of Sciences of the United States of America.

[ref-24] Franklin-Tong VE, Franklin FC (2003). Gametophytic self-incompatibility inhibits pollen tube growth using different mechanisms. Trends in Plant Science.

[ref-25] Fujii S, Kubo K-I, Takayama S (2016). Non-self- and self-recognition models in plant self-incompatibility. Nature Plants.

[ref-26] Gaikwad A, Tewari KK, Kumar D, Chen W, Mukherjee SK (1999). Isolation and characterisation of the cDNA encoding a glycosylated accessory protein of pea chloroplast DNA polymerase. Nucleic Acids Research.

[ref-27] Gausing K (2000). A barley gene (*rsh1*) encoding a ribonuclease S-like homologue specifically expressed in young light-grown leaves. Planta.

[ref-28] Gibbs PE (2014). Late-acting self-incompatibility—the pariah breeding system in flowering plants. New Phytologist.

[ref-29] Golz JF, Oh H-Y, Su V, Kusaba M, Newbigin E (2001). Genetic analysis of *Nicotiana* pollen-part mutants is consistent with the presence of an S-ribonuclease inhibitor at the S locus. Proceedings of the National Academy of Sciences of the United States of America.

[ref-30] Good-Avila SV, Majumder D, Amos H, Stephenson AG (2008). Characterization of self-incompatibility in *Campanula rapunculoides* (Campanulaceae) through genetic analyses and microscopy. Botany.

[ref-31] Groß N, Wasternack C, Köck M (2004). Wound-induced *RNaseLE* expression is jasmonate and systemin independent and occurs only locally in tomato (*Lycopersicon esculentum* cv. Lukullus). Phytochemistry.

[ref-32] Hauck NR, Yamane H, Tao R, Iezzoni AF (2006). Accumulation of nonfunctional S-haplotypes results in the breakdown of gametophytic self-incompatibility in tetraploid *Prunus*. Genetics.

[ref-33] Hillwig MS, Contento AL, Meyer A, Ebany D, Bassham DC, Macintosh GC (2011). RNS2, a conserved member of the RNase T2 family, is necessary for ribosomal RNA decay in plants. Proceedings of the National Academy of Sciences of the United States of America.

[ref-34] Hillwig MS, LeBrasseur ND, Green PJ, MacIntosh GC (2008). Impact of transcriptional, ABA-dependent, and ABA-independent pathways on wounding regulation of *RNS1* expression. Molecular Genetics and Genomics.

[ref-35] Hillwig MS, Liu X, Liu G, Thornburg RW, MacIntosh GC (2010). *Petunia* nectar proteins have ribonuclease activity. Journal of Experimental Botany.

[ref-36] Hillwig MS, Rizhsky L, Wang Y, Umanskaya A, Essner JJ, MacIntosh GC (2009). Zebrafish RNase T2 genes and the evolution of secretory ribonucleases in animals. BMC Evolutionary Biology.

[ref-37] Hino M, Kawano S, Kimura M (2002). Expression of *Nicotiana glutinosa* ribonucleases in *Escherichia coli*. Bioscience, Biotechnology, and Biochemistry.

[ref-38] Hiscock SJ, Kües U, Dickinson HG (1996). Molecular mechanisms of self-incompatibility in flowering plants and fungi—different means to the same end. Trends in Cell Biology.

[ref-39] Ho E, Hayen A, Wilkins MR (2006). Characterisation of organellar proteomes: a guide to subcellular proteomic fractionation and analysis. Proteomics.

[ref-40] Hua Z, Zou C, Shiu S-H, Vierstra RD (2011). Phylogenetic comparison of F-Box (FBX) gene superfamily within the plant kingdom reveals divergent evolutionary histories indicative of genomic drift. PLOS ONE.

[ref-41] Igić B, Kohn JR (2001). Evolutionary relationships among self-incompatibility RNases. Proceedings of the National Academy of Sciences of the United States of America.

[ref-42] Igić B, Lande R, Kohn JR (2008). Loss of self incompatibility and its evolutionary consequences. International Journal of Plant Sciences.

[ref-43] Innan H (2009). Population genetic models of duplicated genes. Genetica.

[ref-44] Ioerger TR, Clark AG, Kao TH (1990). Polymorphism at the self-incompatibility locus in Solanaceae predates speciation. Proceedings of the National Academy of Sciences of the United States of America.

[ref-45] Irie M (1999). Structure-function relationships of acid ribonucleases: lysosomal, vacuolar, and periplasmic enzymes. Pharmacology & Therapeutics.

[ref-46] Jost W, Bak H, Glund K, Terpstra P, Beintema JJ (1991). Amino acid sequence of an extracellular, phosphate-starvation-induced ribonuclease from cultured tomato (*Lycopersicon esculentum*) cells. European Journal of Biochemistry.

[ref-47] Kariu T, Sano K, Shimokawa H, Itoh R, Yamasaki N, Kimura M (1998). Isolation and characterization of a wound-inducible ribonuclease from *Nicotiana glutinosa* leaves. Bioscience, Biotechnology, and Biochemistry.

[ref-48] Katoh K, Standley DM (2013). MAFFT multiple sequence alignment software version 7: improvements in performance and usability. Molecular Biology and Evolution.

[ref-49] Kawano S, Kakuta Y, Nakashima T, Kimura M (2006). Crystal structures of the *Nicotiana glutinosa* ribonuclease NT in complex with nucleoside monophosphates. The Journal of Biochemistry.

[ref-50] Kim SI, Kweon S-M, Kim EA, Kim JY, Kim S, Yoo JS, Park YM (2004). Characterization of RNase-like major storage protein from the ginseng root by proteomic approach. Journal of Plant Physiology.

[ref-51] Kirkwood J, Hargreaves D, O’Keefe S, Wilson J (2015). Using isoelectric point to determine the pH for initial protein crystallization trials. Bioinformatics.

[ref-52] Köck M, Groß N, Stenzel I, Hause G (2004). Phloem-specific expression of the wound-inducible Ribonuclease LE from tomato (*Lycopersicon esculentum cv. Lukullus*). Planta.

[ref-53] Köck M, Stenzel I, Zimmer A (2006). Tissue-specific expression of tomato Ribonuclease LX during phosphate starvation-induced root growth. Journal of Experimental Botany.

[ref-54] Kohn JR (2008). What genealogies of S-alleles tell us. Self-incompatibility in flowering plants: evolution, diversity, and mechanisms.

[ref-55] Köthke S, Köck M (2011). The *Solanum lycopersicum* RNaseLER is a class II enzyme of the RNase T2 family and shows preferential expression in guard cells. Journal of Plant Physiology.

[ref-56] Kubo K-I, Entani T, Takara A, Wang N, Fields AM, Hua Z, Toyoda M, Kawashima S-I, Ando T, Isogai A, Kao T-H, Takayama S (2010). Collaborative non-self recognition system in S-RNase-based self-incompatibility. Science.

[ref-57] Kubo K-I, Paape T, Hatakeyama M, Entani T, Takara A, Kajihara K, Tsukahara M, Shimizu-Inatsugi R, Shimizu KK, Takayama S (2015). Gene duplication and genetic exchange drive the evolution of S-RNase-based self-incompatibility in *Petunia*. Nature Plants.

[ref-58] Kumar S, Filipski AJ, Battistuzzi FU, Kosakovsky Pond SL, Tamura K (2012). Statistics and truth in phylogenomics. Molecular Biology and Evolution.

[ref-59] Kurata N, Kariu T, Kawano S, Kimura M (2002). Molecular cloning of cDNAs encoding ribonuclease-related proteins in *Nicotiana glutinosa* leaves, as induced in response to wounding or to TMV-infection. Bioscience, Biotechnology, and Biochemistry.

[ref-60] Lai Z, Ma W, Han B, Liang L, Zhang Y, Hong G, Xue Y (2002). An F-box gene linked to the self-incompatibility (S) locus of *Antirrhinum* is expressed specifically in pollen and tapetum. Plant Molecular Biology.

[ref-61] LeBrasseur ND, MacIntosh GC, Pérez-Amador MA, Saitoh M, Green PJ (2002). Local and systemic wound-induction of RNase and nuclease activities in *Arabidopsis*: RNS1 as a marker for a JA-independent systemic signaling pathway. The Plant Journal.

[ref-62] Lers A, Khalchitski A, Lomaniec E, Burd S, Green PJ (1998). Senescence-induced RNases in tomato. Plant Molecular Biology.

[ref-63] Lers A, Sonego L, Green PJ, Burd S (2006). Suppression of LX ribonuclease in tomato results in a delay of leaf senescence and abscission. Plant Physiology.

[ref-64] Liang L, Lai Z, Ma W, Zhang Y, Xue Y (2002). *AhSL28*, a senescence-and phosphate starvation-induced S-like RNase gene in *Antirrhinum*. Biochimica et Biophysica Acta (BBA)—Gene Structure and Expression.

[ref-65] Liu W, Fan J, Li J, Song Y, Li Q, Zhang Y, Xue Y (2014). SCF^SLF^-mediated cytosolic degradation of S-RNase is required for cross-pollen compatibility in S-RNase-based self-incompatibility in *Petunia hybrida*. Frontiers in Genetics.

[ref-66] Löffler A, Abel S, Jost W, Beintema JJ, Glund K (1992). Phosphate-regulated induction of intracellular ribonucleases in cultured tomato (*Lycopersicon esculentum*) cells. Plant Physiology.

[ref-67] Luu DT, Qin X, Morse D, Cappadocia M (2000). S-RNase uptake by compatible pollen tubes in gametophytic self-incompatibility. Nature.

[ref-68] Ma RC, Oliveira MM (2000). The *RNase PD2* gene of almond (*Prunus dulcis*) represents an evolutionarily distinct class of S-like RNase genes. Molecular and General Genetics.

[ref-69] MacIntosh GC (2011). RNase T2 family: enzymatic properties, functional diversity, and evolution of ancient ribonucleases. Nucleic acids and molecular biology.

[ref-70] MacIntosh GC, Bariola PA, Newbigin E, Green PJ (2001). Characterization of Rny1, the *Saccharomyces cerevisiae* member of the T2 RNase family of RNases: unexpected functions for ancient enzymes?. Proceedings of the National Academy of Sciences of the United States of America.

[ref-71] MacIntosh GC, Hillwig MS, Meyer A, Flagel L (2010). RNase T2 genes from rice and the evolution of secretory ribonucleases in plants. Molecular Genetics and Genomics.

[ref-72] Matsumoto D, Tao R (2016). Distinct self-recognition in the *Prunus* S-RNase-based gametophytic self-incompatibility system. The Horticulture Journal.

[ref-73] Matton DP, Nass N, Clarke AE, Newbigin E (1994). Self-incompatibility: how plants avoid illegitimate offspring. Proceedings of the National Academy of Sciences of the United States of America.

[ref-74] McClure BA, Haring V, Ebert PR, Anderson MA, Simpson RJ, Sakiyama F, Clarke AE (1989). Style self-incompatibility gene products of *Nicotlana alata* are ribonucleases. Nature.

[ref-75] Morimoto T, Akagi T, Tao R (2015). Evolutionary analysis of genes for S-RNase-based self-incompatibility reveals S locus duplications in the ancestral Rosaceae. The Horticulture Journal.

[ref-76] Nasrallah JB, Kao T-H, Goldberg ML, Nasrallah ME (1985). A cDNA clone encoding an S-locus-specific glycoprotein from *Brassica oleracea*. Nature.

[ref-77] Nishimura E, Jumyo S, Arai N, Kanna K, Kume M, Nishikawa J-I, Tanase J-I, Ohyama T (2014). Structural and functional characteristics of S-like ribonucleases from carnivorous plants. Planta.

[ref-78] Nishimura E, Kawahara M, Kodaira R, Kume M, Arai N, Nishikawa J-I, Ohyama T (2013). S-like ribonuclease gene expression in carnivorous plants. Planta.

[ref-79] Nowak MD, Davis AP, Anthony F, Yoder AD (2011). Expression and trans-specific polymorphism of self-incompatibility RNases in *Coffea* (Rubiaceae). PLOS ONE.

[ref-80] Nürnberger T, Abel S, Jost W, Glund K (1990). Induction of an extracellular ribonuclease in cultured tomato cells upon phosphate starvation. Plant Physiology.

[ref-81] Ohkama-Ohtsu N, Kishimoto N, Yazaki J, Fujii F, Shinbo K, Shimatani Z, Nagata Y, Hashimoto A, Ohta T, Sato Y, Honda S, Yamamoto K, Sakata K, Sasaki T, Kikuchi S, Hayashi H, Yoneyama T, Fujiwara T (2004). Upregulation of the genes for ferritin, RNase, and DnaJ in leaves of rice plants in response to sulfur deficiency. Soil Science and Plant Nutrition.

[ref-82] Okabe T, Iwakiri Y, Mori H, Ogawa T, Ohyama T (2005). An S-like ribonuclease gene is used to generate a trap-leaf enzyme in the carnivorous plant *Drosera adelae*. FEBS Letters.

[ref-83] Qiao H, Wang H, Zhao L, Zhou J, Huang J, Zhang Y, Xue Y (2004). The F-box protein AhSLF-S2 physically interacts with S-RNases that may be inhibited by the ubiquitin/26S proteasome pathway of protein degradation during compatible pollination in *Antirrhinum*. The Plant Cell.

[ref-84] Richman AD, Broothaerts W, Kohn JR (1997). Self-incompatibility RNases from three plant families: homology or convergence?. American Journal of Botany.

[ref-85] Richman AD, Kohn JR (1996). Learning from rejection: the evolutionary biology of single-locus incompatibility. Trends in Ecology & Evolution.

[ref-86] Ronquist F, Teslenko M, Van der Mark P, Ayres DL, Darling A, Höhna S, Larget B, Liu L, Suchard MA, Huelsenbeck JP (2012). MrBayes 3.2: efficient Bayesian phylogenetic inference and model choice across a large model space. Systematic Biology.

[ref-87] Sassa H, Nishio T, Kowyama Y, Hirano H, Koba T, Ikehashi H (1996). Self-incompatibility (*S*) alleles of the Rosaceae encode members of a distinct class of the T2/S ribonuclease superfamily. Molecular and General Genetics.

[ref-88] Shubin N, Tabin C, Carroll SB (1997). Fossils, genes and the evolution of animal limbs. Nature.

[ref-89] Sijacic P, Wang X, Skirpan AL, Wang Y, Dowd PE, McCubbin AG, Huang S, Kao T-H (2004). Identification of the pollen determinant of S-RNase-mediated self-incompatibility. Nature.

[ref-90] Slater GSC, Birney E (2005). Automated generation of heuristics for biological sequence comparison. BMC Bioinformatics.

[ref-91] Song G, Hsu C-H, Riemer C, Miller W (2011). Evaluation of methods for detecting conversion events in gene clusters. BMC Bioinformatics.

[ref-92] Stamatakis A (2014). RAxML version 8: a tool for phylogenetic analysis and post-analysis of large phylogenies. Bioinformatics.

[ref-93] Steinbachs JE, Holsinger KE (2002). S-RNase-mediated gametophytic self-incompatibility is ancestral in eudicots. Molecular Biology and Evolution.

[ref-94] Sukumaran J, Holder MT (2010). DendroPy: a python library for phylogenetic computing. Bioinformatics.

[ref-95] Sutherland BG, Tobutt KR, Robbins TP (2008). Trans-specific S-RNase and SFB alleles in *Prunus* self-incompatibility haplotypes. Molecular Genetics and Genomics.

[ref-96] Takayama S, Isogai A (2005). Self-incompatibility in plants. Annual Review of Plant Biology.

[ref-97] Tanaka N, Arai J, Inokuchi N, Koyama T, Ohgi K, Irie M, Nakamura KT (2000). Crystal structure of a plant ribonuclease, RNase LE. Journal of Molecular Biology.

[ref-98] Tao R, Watari A, Hanada T, Habu T, Yaegaki H, Yamaguchi M, Yamane H (2007). Self-compatible peach (*Prunus persica*) has mutant versions of the S haplotypes found in self-incompatible *Prunus* species. Plant Molecular Biology.

[ref-99] Taylor CB, Bariola PA, DelCardayré SB, Raines RT, Green PJ (1993). RNS2: a senescence-associated RNase of *Arabidopsis* that diverged from the S-RNases before speciation. Proceedings of the National Academy of Sciences of the United States of America.

[ref-100] The Angiosperm Phylogeny Group (2016). An update of the angiosperm phylogeny group classification for the orders and families of flowering plants: APG IV. Botanical Journal of the Linnean Society.

[ref-101] Thompson DM, Lu C, Green PJ, Parker R (2008). tRNA cleavage is a conserved response to oxidative stress in eukaryotes. RNA.

[ref-102] True JR, Carroll SB (2002). Gene co-option in physiological and morphological evolution. Annual Review of Cell and Developmental Biology.

[ref-103] Ushijima K, Sassa H, Dandekar AM, Gradziel TM, Tao R, Hirano H (2003). Structural and transcriptional analysis of the self-incompatibility locus of almond: identification of a pollen-expressed F-box gene with haplotype-specific polymorphism. The Plant Cell.

[ref-104] Ushijima K, Yamane H, Watari A, Kakehi E, Ikeda K, Hauck NR, Iezzoni AF, Tao R (2004). The S haplotype-specific F-box protein gene, SFB, is defective in self-compatible haplotypes of *Prunus avium* and *P. mume*. The Plant Journal.

[ref-105] Van Damme EJM, Hao Q, Barre A, Rougé P, Van Leuven F, Peumans WJ (2000). Major protein of resting rhizomes of *Calystegia sepium* (hedge bindweed) closely resembles plant RNases but has no enzymatic activity. Plant Physiology.

[ref-106] Vaughan SP, Russell K, Sargent DJ, Tobutt KR (2006). Isolation of S-locus F-box alleles in *Prunus avium* and their application in a novel method to determine self-incompatibility genotype. Theoretical and Applied Genetics.

[ref-107] Vieira J, Fonseca NA, Vieira CP (2008). An S-RNase-based gametophytic self-incompatibility system evolved only once in eudicots. Journal of Molecular Evolution.

[ref-108] Vieira J, Morales-Hojas R, Santos RAM, Vieira CP (2007). Different positively selected sites at the gametophytic self-incompatibility pistil S-RNase gene in the Solanaceae and Rosaceae (*Prunus*, *Pyrus*, and *Malus*). Journal of Molecular Evolution.

[ref-109] Wang L, Dong L, Zhang Y, Zhang Y, Wu W, Deng X, Xue Y (2004). Genome-wide analysis of S-Locus F-box-like genes in *Arabidopsis thaliana*. Plant Molecular Biology.

[ref-110] Warren DL, Geneva AJ, Lanfear R (2017). RWTY (R We There Yet): an R package for examining convergence of Bayesian phylogenetic analyses. Molecular Biology and Evolution.

[ref-111] Wei JY, Li AM, Li Y, Wang J, Liu XB, Liu LS, Xu ZF (2006). Cloning and characterization of an RNase-related protein gene preferentially expressed in rice stems. Bioscience, Biotechnology, and Biochemistry.

[ref-112] Williams JS, Der JP, DePamphilis CW, Kao T-H (2014). Transcriptome analysis reveals the same 17 S-Locus F-Box genes in two haplotypes of the self-incompatibility locus of *Petunia inflata*. The Plant Cell.

[ref-113] Williams JS, Wu L, Li S, Sun P, Kao T-H (2015). Insight into S-RNase-based self-incompatibility in *Petunia*: recent findings and future directions. Frontiers in Plant Science.

[ref-114] Xue Y, Carpenter R, Dickinson HG, Coen ES (1996). Origin of allelic diversity in *Antirrhinum* S locus RNases. The Plant Cell.

[ref-115] Xue Y, Zhang Y, Yang Q, Li Q, Cheng Z, Dickinson HG (2009). Genetic features of a pollen-part mutation suggest an inhibitory role for the *Antirrhinum* pollen self-incompatibility determinant. Plant Molecular Biology.

[ref-116] Yamane H, Ikeda K, Ushijima K, Sassa H, Tao R (2003a). A pollen-expressed gene for a novel protein with an F-box motif that is very tightly linked to a gene for S-RNase in two species of cherry, *Prunus cerasus* and *P. avium*. Plant and Cell Physiology.

[ref-117] Yamane H, Ushijima K, Sassa H, Tao R (2003b). The use of the S haplotype-specific F-box protein gene, SFB, as a molecular marker for S-haplotypes and self-compatibility in Japanese apricot (*Prunus mume*). Theoretical and Applied Genetics.

[ref-118] Yang X, Kalluri UC, Jawdy S, Gunter LE, Yin T, Tschaplinski TJ, Weston DJ, Ranjan P, Tuskan GA (2008). The F-box gene family is expanded in herbaceous annual plants relative to woody perennial plants. Plant Physiology.

[ref-119] Zhou J, Wang F, Ma W, Zhang Y, Han B, Xue Y (2003). Structural and transcriptional analysis of S-locus F-box genes in *Antirrhinum*. Sexual Plant Reproduction.

