# Supplementary material for: The evolutionary history of plant T2/S-type ribonucleases
Source: PeerJ. 2017 Sep 11;5:e3790. doi: 10.7717/peerj.3790 (PMC5598434; doi:10.7717/peerj.3790)
Supplement: Supplemental Information 1 — A list of ENTREZ search terms used to obtain T2/S-RNase sequences from GenBank. List of species removed from the dataset. Gene tree of T2/S-RNases in land plants with tip names and other annotations. The relationship between calculated pI values and experimental pH optima for several T2/S-RNases in land plants. Gene tree of F-box-containing genes near T2/S-RNase loci. Table of GenBank accessions used. Table of the referenced functional T2/S-RNase studies. Table of genomes used. [file peerj-05-3790-s001.pdf]

Contents

S1 Protein database ENTREZ query **2**

S2 Nucleotide blast search ENTREZ query **2**

S3 Species Filtered from GenBank Results **2**

List of Figures

S1 Phylogenetic relationships between T2/S-RNases in land plants. 3
- S1a Class II 3
- S1b Class III, Section A 9
- S1c Class III, Section B 9
- S1d Class I, Section A 9
- S1e Class I, Section B 9
- S1f Class I, Section C 9

S2 The relationship between pI values and pH optima for several T2/S-type RNases in land plants. 9

S3 Gene tree of F-box-containing genes near T2/S-type RNases. 10

List of Tables

S1 Accessions and Genomic Sequences Used in this Study 11

S2 Genomes Examined 63

S3 Studies of Expression Patterns and Function of T2/S-RNases 67

S1 Protein database ENTREZ query

```
(RNase OR Ribonuclease) AND T2 AND 150:400[Sequence Length] AND
txid33090[Organism] AND srcdb_refseq[PROP]
```

S2 Nucleotide blast search ENTREZ query

```
300:10000[Sequence Length] AND txid33090[Organism]
```

S3 Species Filtered from GenBank Results

Antirrhinum majus × *Antirrhinum hispanicum*
Aquilegia formosa × *Aquilegia pubescens*
Chrysanthemum × *morifolium*
Citrus × *paradisi*
Coffea arabica
Gerbera hybrid
Juglans hindsii × *Juglans regia*
Leymus cinereus × *Leymus triticoides*
Malus domestica
Musa ABB
Nicotiana tabacum
Oncidium hybrid
Petunia × *hybrida*
Picea engelmannii × *Picea glauca*
Populus alba × *Populus glandulosa*
Populus fremontii × *Populus angustifolia*
Populus tremula × *Populus alba*
Populus tremula × *Populus tremuloides*
Populus trichocarpa × *Populus deltoides*
Populus trichocarpa × *Populus nigra*
Prunus × *yedoensis*
Pyrus × *bretschneideri*
Pyrus hybrid
Rosa hybrid
Rubus ulmifolius × *Rubus thyrsiger*
Saccharum hybrid

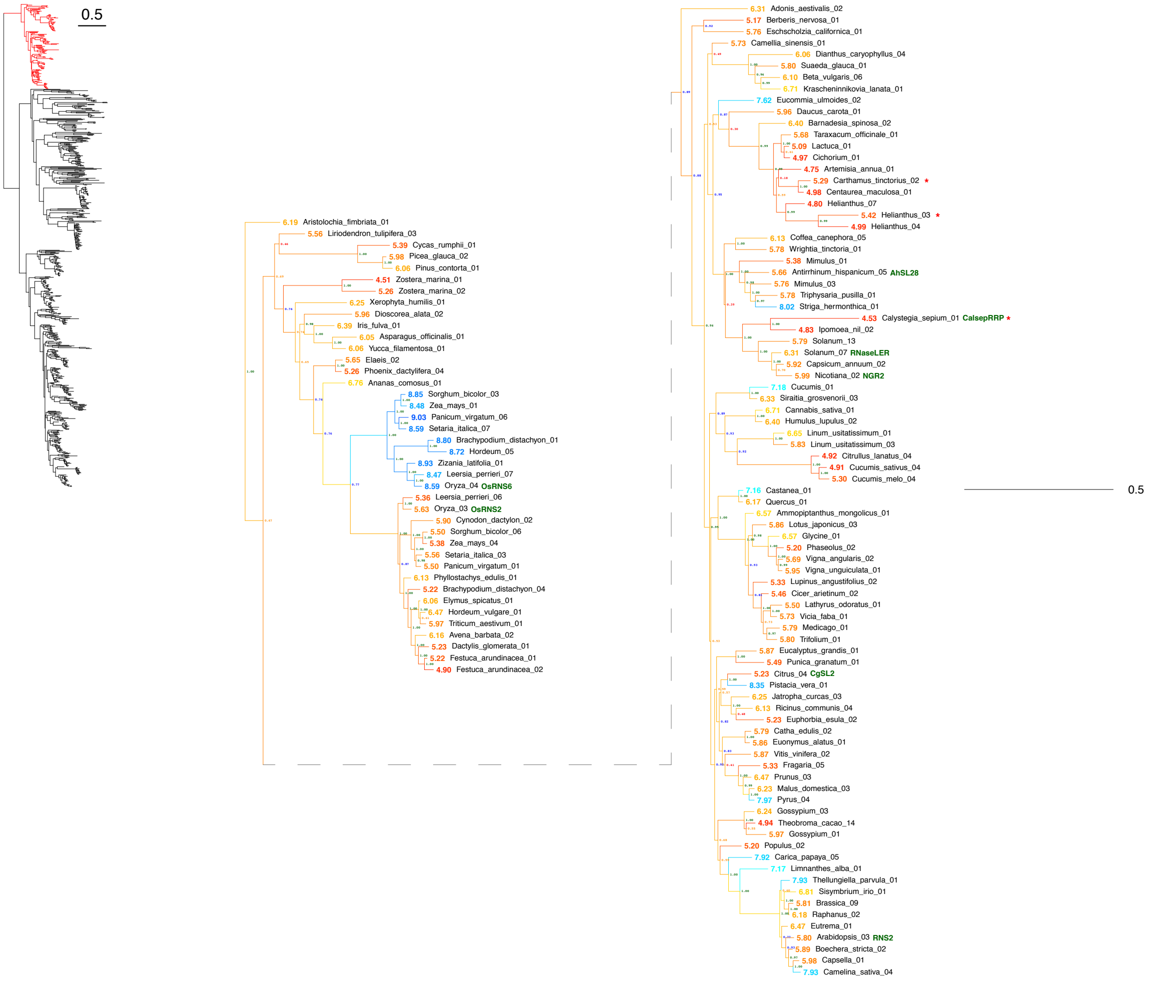

(a) Class II

Figure S1: Phylogenetic relationships between T2/S-RNases in land plants. This is a more detailed version of the Figure 2. The rooted maximum-likelihood phylogram is shown, along with posterior support. Numbers to the right of the tree tips indicate the predicted isoelectric point (pI) value of the amino acid sequence. Corresponding tree tips are colored using these values, and values for the internal branches were reconstructed as described in the Methods. S-RNase alleles are indicated with dark blue "S-RNase". The names of previously studied proteins are given in dark green. Red stars show sequences known to lack RNase function, or are inferred to lack the function based on absence of a histidine thought to be essential for that function.

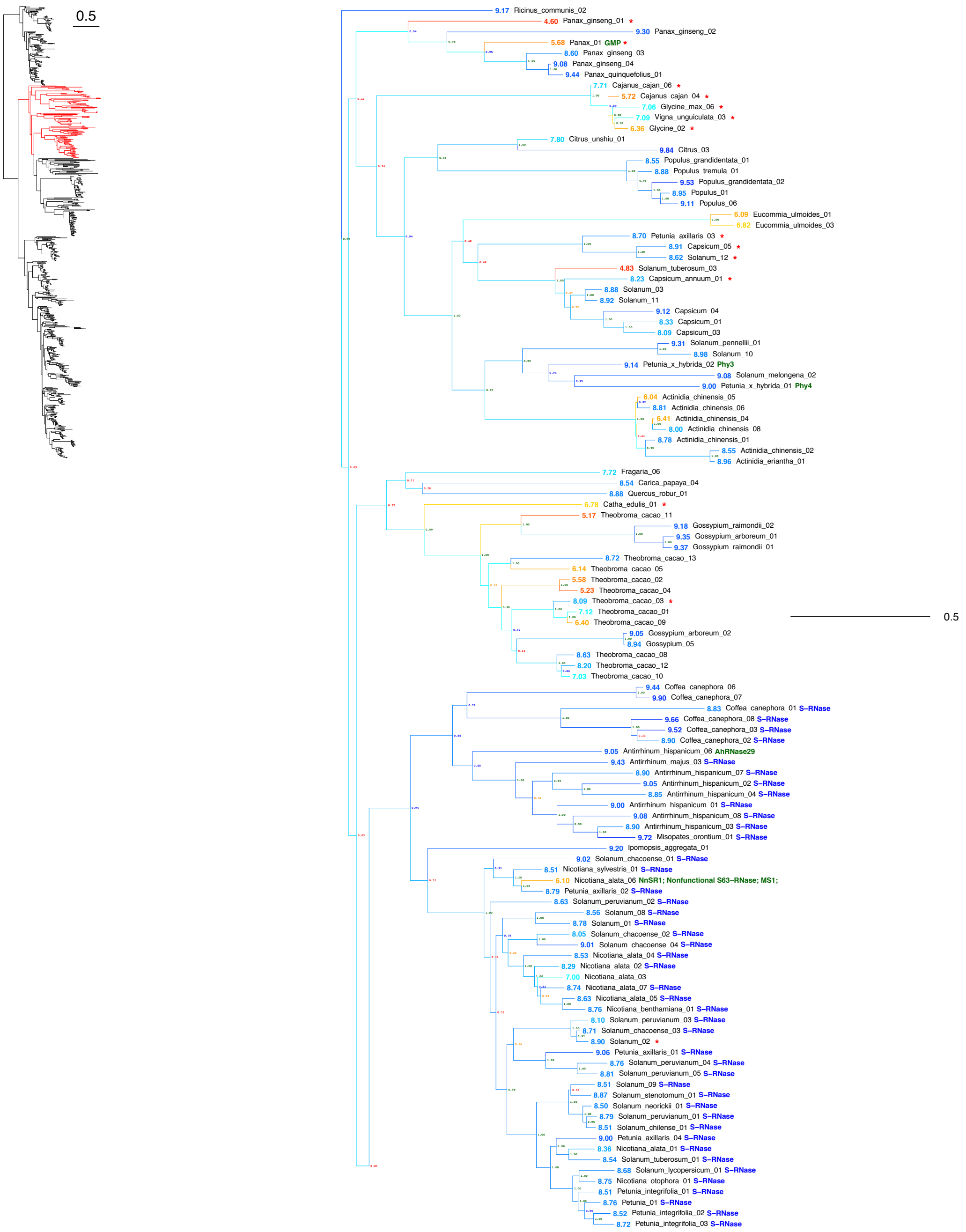

(b) Class III, Section A

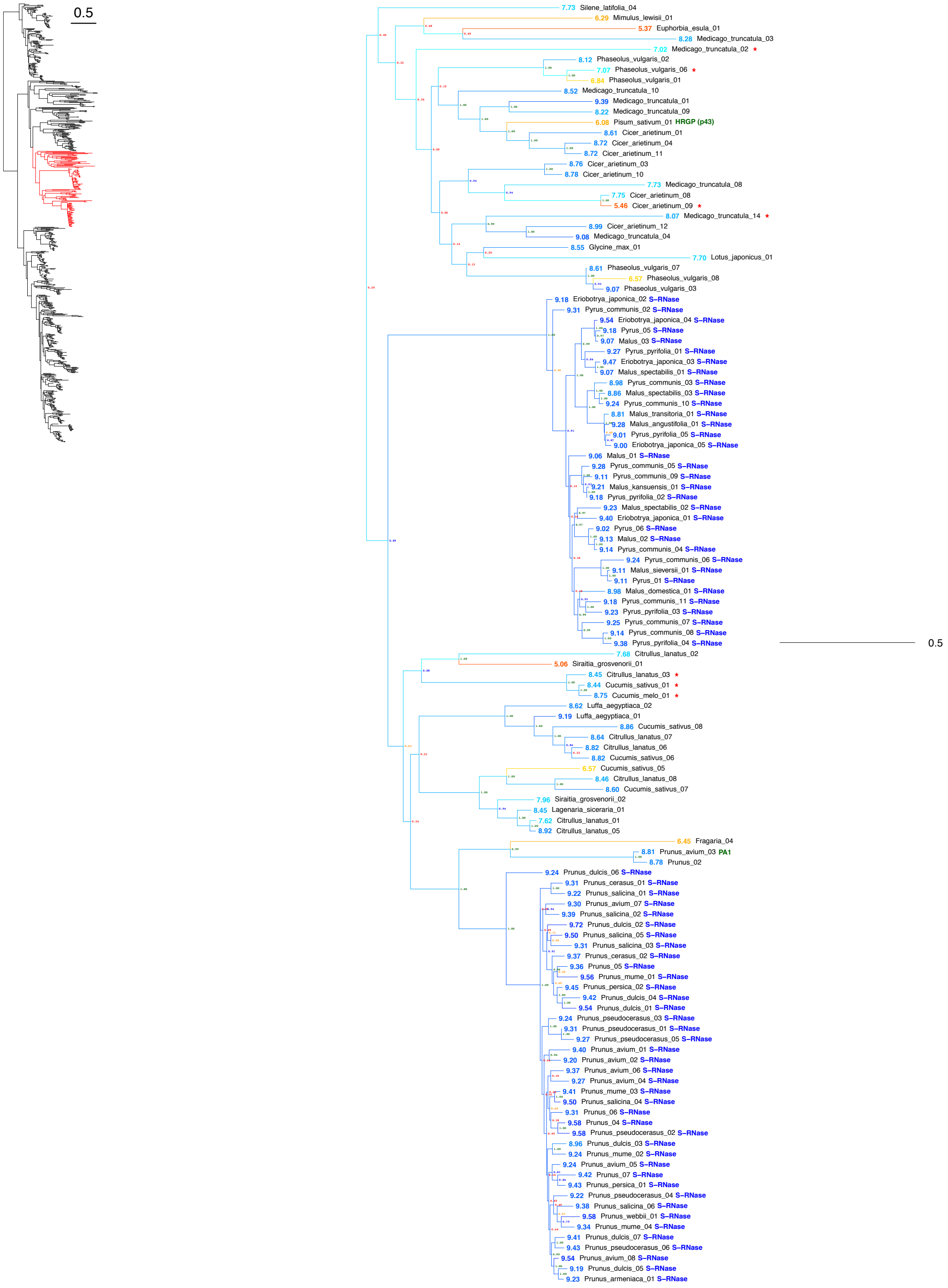

(c) Class III, Section B

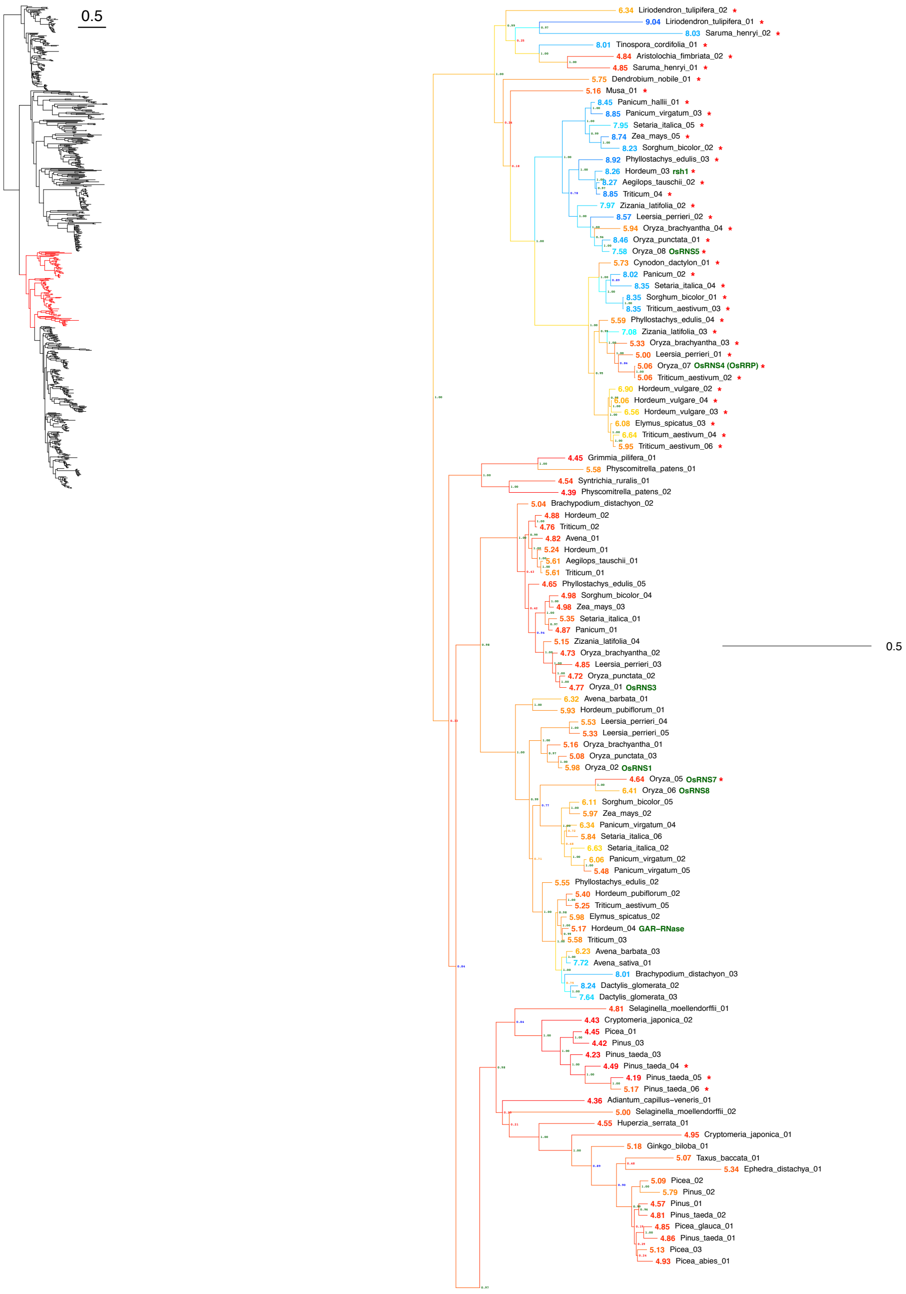

(d) Class I, Section A

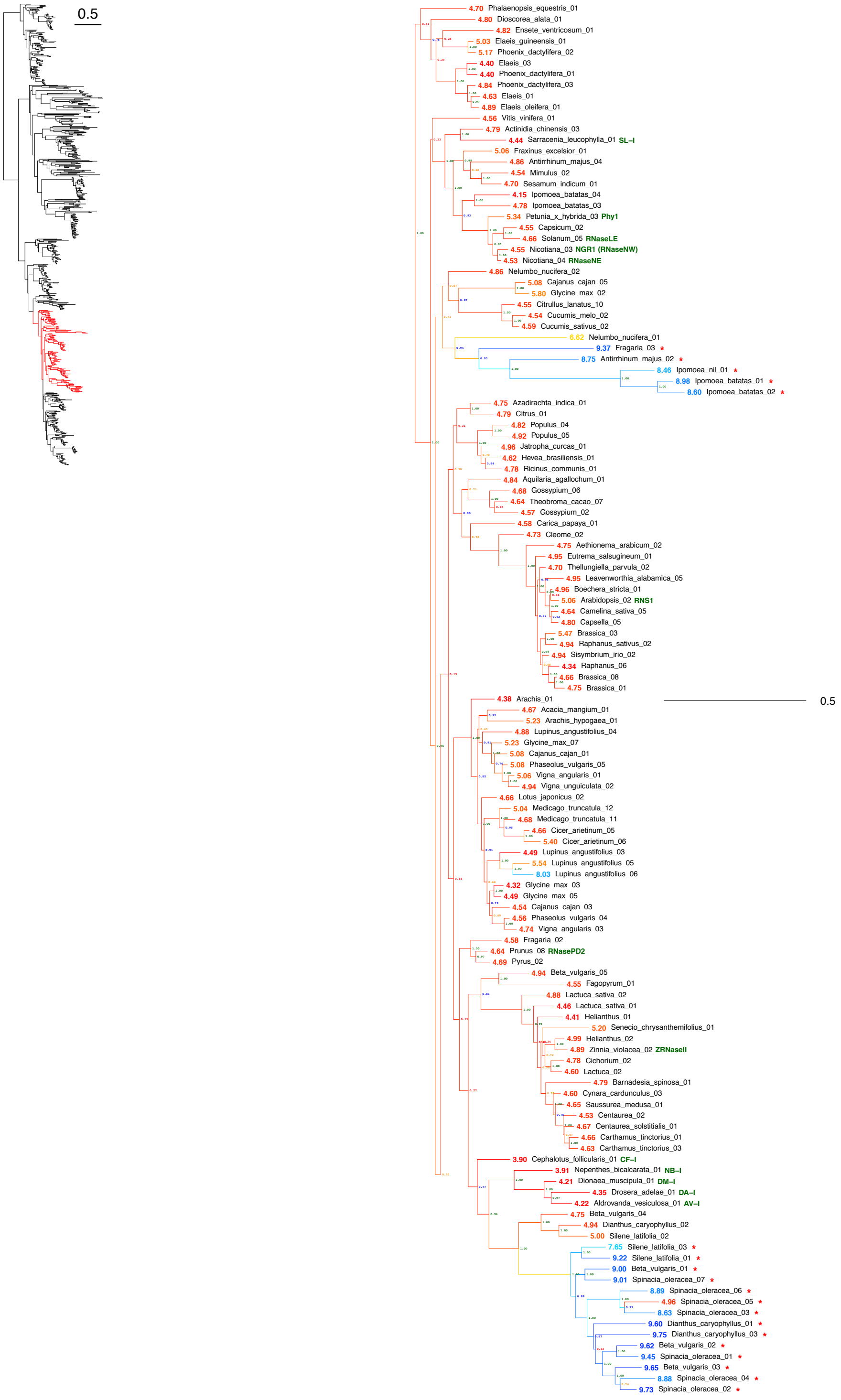

(e) Class I, Section B

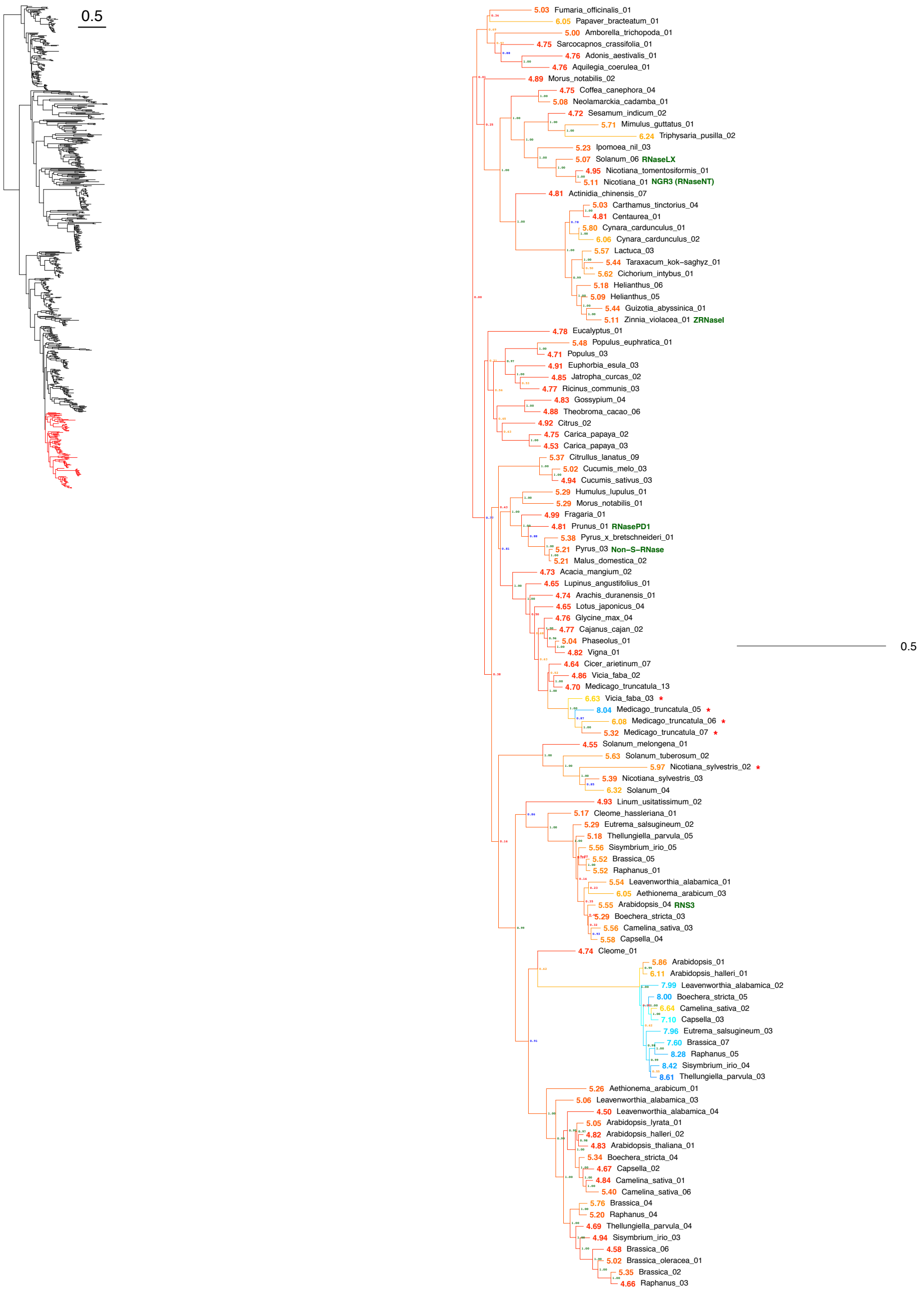

(f) Class I, Section C

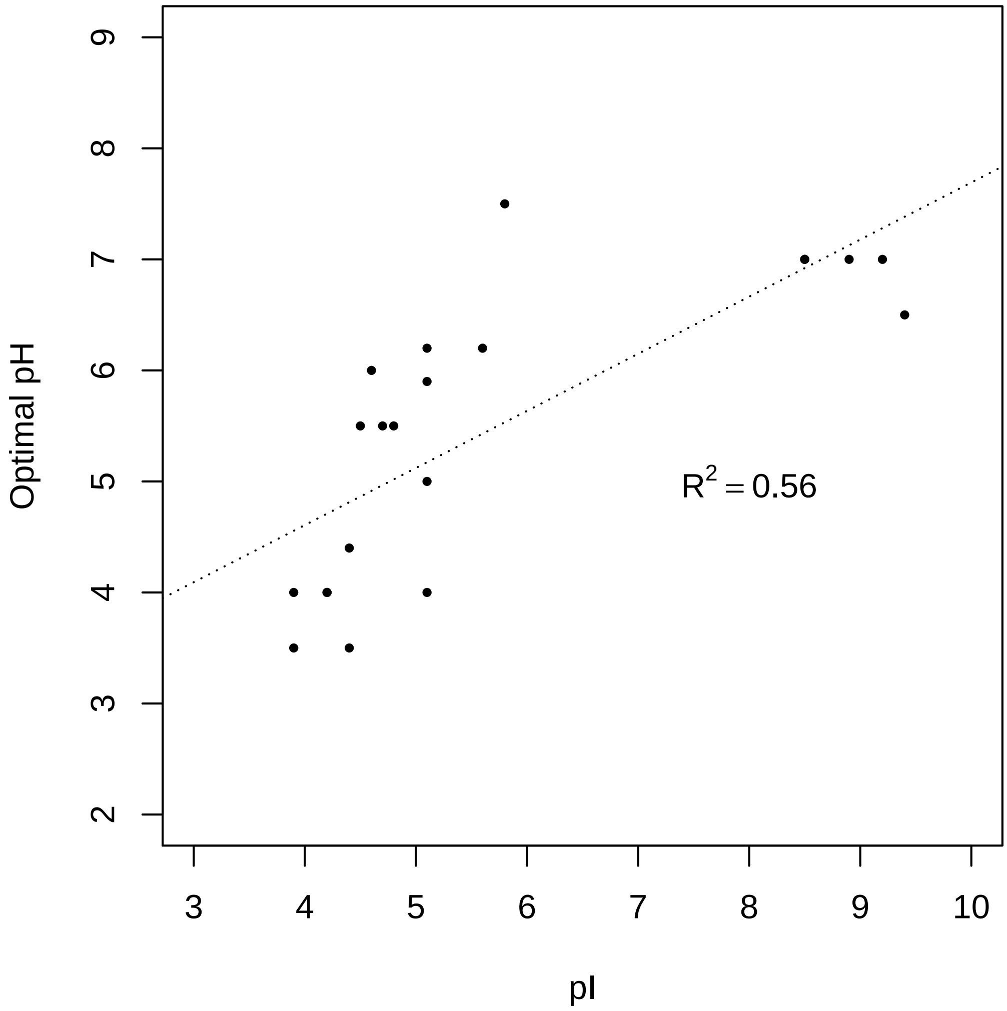

Figure S2: The relationship between calculated pI values and experimental pH optima for several T2/S-type RNases in land plants. The linear relationship is drawn only for visual reference.

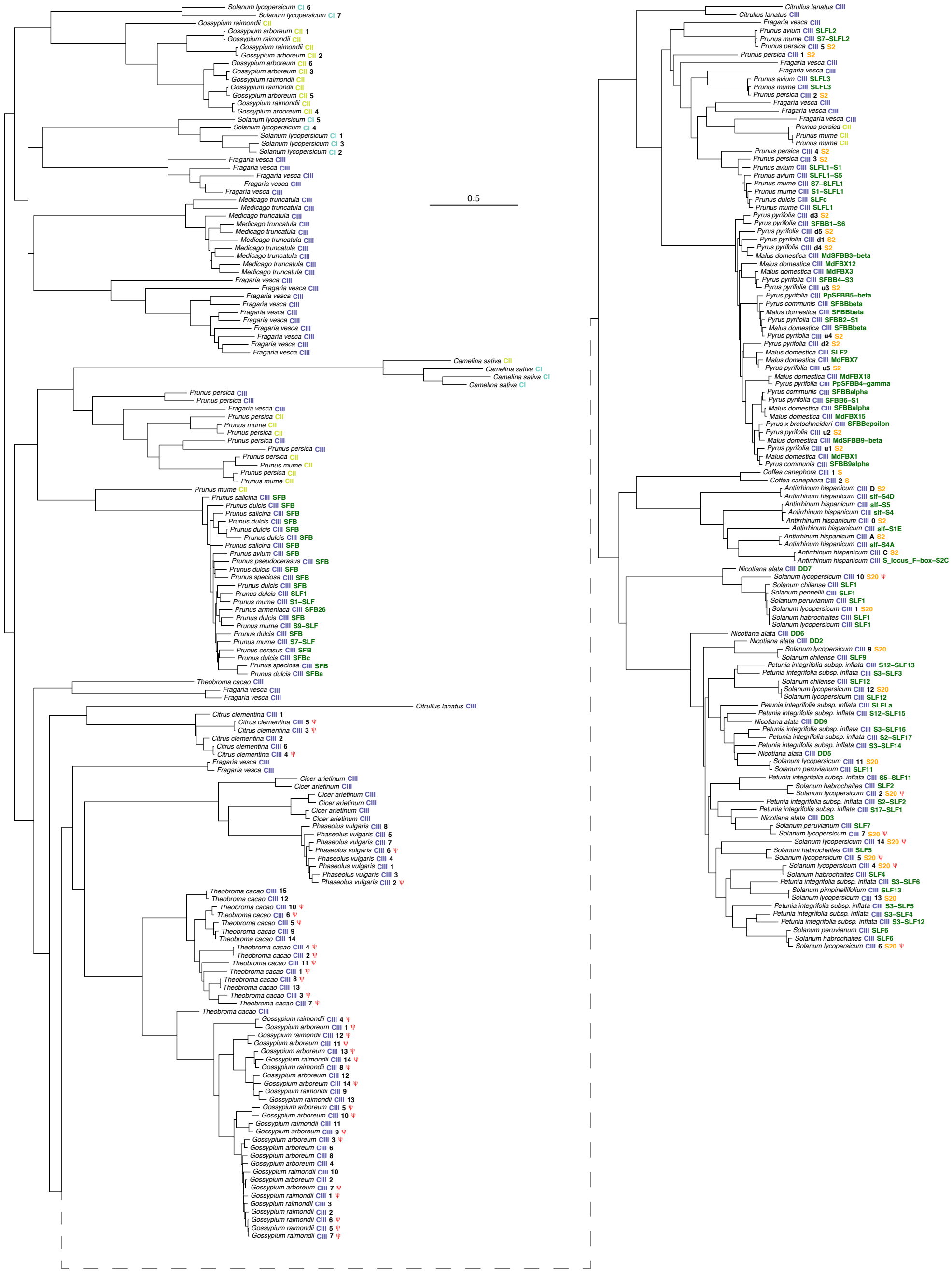

Figure S3: Gene tree of F-box-containing genes near T2/S-type RNases. 'Class' of the closest linked RNase is indicated immediately to the right of the species name. This figure includes all the F-box genes shown in Figure 5, and several other known S-locus F-box genes (names given in dark green). Black bold letters following the class designation indicate the F-box locus designation, and match the numbers given in Figure 5. Where available, the linked S-RNase allele is given (in orange). Possible pseudogenes—sequences with stop codons that prematurely interrupt reading frames—are indicated by ψ.

Table S1: Accessions and Genomic Sequences Used in this Study

**Tip Name**	**EST**	**NT**	**Genomic**
Acacia_mangium_01	FS588527.1	-	-
Acacia_mangium_02	FS590467.1	-	-
Actinidia_chinensis_01	-	-	Achinensi_04
Actinidia_chinensis_02	FG491469.1, FG495535.1, FG497037.1, FG497281.1, FG500389.1, FG500436.1, FG501326.1, FG513143.1	-	Achinensi_07, Achinensi_10
Actinidia_chinensis_03	-	-	Achinensi_15
Actinidia_chinensis_04	FG495163.1	-	Achinensi_03
Actinidia_chinensis_05	FG488689.1, FG489605.1, FG490006.1, FG491542.1, FG494339.1, FG494455.1, FG494563.1, FG496651.1, FG498987.1, FG501418.1, FG525523.1, FG526342.1, FG527807.1	-	-
Actinidia_chinensis_06	FG488513.1, FG490909.1, FG492393.1, FG492662.1, FG493600.1, FG495376.1, FG496414.1, FG496613.1, FG498230.1, FG499004.1, FG500280.1, FG501523.1, FG502059.1, FG513718.1, FG514133.1	-	-
Actinidia_chinensis_07	FG526389.1, FG526432.1, FG527623.1	-	-
Actinidia_chinensis_08	FG511324.1, FG517989.1, FG525987.1, FG527061.1	-	-
Actinidia_eriantha_01	FG482843.1, FG508897.1	-	-
Adiantum_capillus-veneris_01	DK953962.1, DK956820.1	-	-
Adonis_aestivalis_01	FL510152.1	-	-
Adonis_aestivalis_02	FL509121.1, FL509488.1, FL511827.1	-	-
Aegilops_tauschii_01	-	-	Atauschii_06
Aegilops_tauschii_02	-	-	Atauschii_07
Aethionema_arabicum_01	-	-	Aarabicum_02
Aethionema_arabicum_02	-	-	Aarabicum_03
Aethionema_arabicum_03	-	-	Aarabicum_04
Aldrovanda_vesiculosa_01	-	AB872467.1	-
Amborella_trichopoda_01	CK760482.1, FD441563.1	XM_006827195.1	Atrichopo_01
Ammopiptanthus_mongolicus_01	JZ389161.1	-	-

Tip Name	EST	NT	Genomic
Ananas_comosus_01	CO730744.1, CO730750.1, CO730775.1, CO730783.1, CO730849.1, CO730906.1, CO730924.1, CO731011.1, CO731024.1, CO731029.1, CO731051.1, CO731055.1, CO731074.1, CO731107.1, CO731116.1, CO731327.1, CO731361.1, CO731368.1, CO731380.1, CO731389.1, CO731395.1, CO731422.1, CO731507.1, CO731516.1, CO731556.1, CO731727.1, CO731737.1, CO731744.1, CO731762.1, CO731791.1, CO731795.1, CO731814.1, CO731831.1, CO731875.1, CO731898.1, CO731921.1, CO731942.1, CO731946.1, CO731967.1, CO732011.1, CO732026.1, CO732028.1, CO732033.1, CO732042.1, CO732080.1, CO732102.1, CO732125.1, CO732157.1, CO732179.1, CO732210.1, CO732212.1, CO732250.1, CO732253.1	-	-
Antirrhinum_hispanicum_01	-	X96465.1	-
Antirrhinum_hispanicum_02	-	X96466.1	-
Antirrhinum_hispanicum_03	-	X96464.1	-
Antirrhinum_hispanicum_04	-	AJ315593.1	-
Antirrhinum_hispanicum_05	-	AJ315591.1	-
Antirrhinum_hispanicum_06	-	AJ315592.1	-
Antirrhinum_hispanicum_07	-	AJ440730.1	-
Antirrhinum_hispanicum_08	-	HE805271.1	-
Antirrhinum_majus_02	AJ793159.1, AJ808829.1	-	-
Antirrhinum_majus_03	AJ790678.1, AJ804705.1	-	-
Antirrhinum_majus_04	AJ802912.1, AJ808165.1	-	-
Aquilaria_agallochum_01	-	-	Aagalloch_01
Aquilegia_coerulea_01	-	-	Acoerulea_01
Arabidopsis_halleri_01	-	-	Ahalleri_03
Arabidopsis_halleri_02	-	-	Ahalleri_04
Arabidopsis_lyrata_01	-	XM_002892750.1	Alyrata_02
Arabidopsis_thaliana_01	-	NM_101288.1	Athaliana_02

Tip Name	EST	NT	Genomic
Arabidopsis_01	AI998583.1, AV537553.1, AV538896.1, AV549500.1, AV551274.1, BE037901.1, BP571466.1	BT010136.1, BX816723.1, NM_101287.3, XM_002892749.1	Alyrata_01, Athaliana_01
Arabidopsis_02	AV553021.1, AV830199.1, BE523813.1, CB252787.1, CB254203.1, CB255251.1, DR374194.1, EG517946.1, EG517947.1	AY072413.1, AY086747.1, AY114710.1, BX821791.1, DQ446504.1, DQ652992.1, NM_126351.2, U05206.1, XM_002875137.1	Alyrata_05, Athaliana_04
Arabidopsis_03	AV527174.1, AV825172.1, CD529387.1, CK121456.1, DR268917.1, DR268918.1, DR268919.1, DR268920.1, DR268921.1, DR268923.1, DR268925.1, DR268927.1, DR268928.1, DR268929.1, DR268930.1, DR268934.1, DR268935.1, DR268936.1, DR268937.1	BT025792.1, BX819005.1, BX819504.1, BX821463.1, M98336.1, NM_129536.4, XM_002879771.1	Alyrata_04, Athaliana_05
Arabidopsis_04	AV824900.1, AV828266.1, BP811763.1, DR279160.1, DR279162.1, DR279163.1, DR279164.1, DR279165.1, DR279166.1, DR279167.1, DR279168.1, DR279170.1, DR279171.1, DR371552.1, DR377825.1	AY063844.1, AY088821.1, AY117288.1, BX816205.1, BX818538.1, NM_102446.2, U05207.1, XM_002890585.1	Ahalleri_01, Alyrata_03, Athaliana_03
Arachis_duranensis_01	GW927913.1, GW928481.1, GW934909.1, GW936332.1, GW938313.1, GW938837.1, GW947666.1, GW955425.1	-	-
Arachis_hypogaea_01	EE127593.1, ES759700.1, GO259579.1	-	-
Arachis_01	EH042149.1, EH042940.1, EH043002.1, EH043293.1, GW956370.1, GW987623.1	-	-
Aristolochia_fimbriata_01	FD755703.1	-	-
Aristolochia_fimbriata_02	FD755819.1, FD763976.1, FD764154.1, FD764251.1	-	-
Artemisia_annua_01	EY110594.1, EY110595.1	-	-

Tip Name	EST	NT	Genomic
Asparagus_officinalis_01	CV291711.1	-	-
Avena_barbata_01	GR363868.1	-	-
Avena_barbata_02	GR366131.1	-	-
Avena_barbata_03	GR350960.1, GR350961.1, GR351290.1, GR351291.1	-	-
Avena_sativa_01	GO594607.1	-	-
Avena_01	GO594668.1, GR343335.1, GR343336.1, GR361714.1, GR361715.1, GR366858.1, GR366859.1	-	-
Azadirachta_indica_01	-	-	Aindica_02
Barnadesia_spinosa_01	GE523589.1	-	-
Barnadesia_spinosa_02	GE548969.1	-	-
Berberis_nervosa_01	JG621270.1	-	-
Beta_vulgaris_01	CK136765.1, CV301955.1, CV301956.1, CV301970.1, FG344579.1	-	Bvulgaris_04
Beta_vulgaris_02	-	-	Bvulgaris_01
Beta_vulgaris_03	-	-	Bvulgaris_02
Beta_vulgaris_04	-	-	Bvulgaris_05
Beta_vulgaris_05	-	-	Bvulgaris_06
Beta_vulgaris_06	-	-	Bvulgaris_07
Boechera_stricta_01	-	-	Bstricta_05
Boechera_stricta_02	-	-	Bstricta_04
Boechera_stricta_03	-	-	Bstricta_01
Boechera_stricta_04	-	-	Bstricta_02
Boechera_stricta_05	-	-	Bstricta_03
Brachypodium_distachyon_01	-	XM_003567311.1	Bdistachy_03
Brachypodium_distachyon_02	DV469105.1, DV469570.1, DV469893.1, DV470064.1, DV470073.1, DV470214.1, DV470623.1, DV470669.1, DV471802.1, DV471970.1, DV472223.1, DV480909.1, DV482102.1, DV485062.1, HX799208.1, HX810128.1, HX813127.1, HX849197.1	AK428392.1, AK432789.1, XM_003574507.1	-
Brachypodium_distachyon_03	DV469148.1, DV469525.1, DV472668.1, DV478177.1, DV479070.1, DV479431.1, DV487490.1, DV487610.1, DV487695.1, GT768236.1	XM_003562584.1	Bdistachy_01

**Tip Name**	**EST**	**NT**	**Genomic**
Brachypodium_distachyon_04	GT802058.1, GT802059.1, GT802801.1, GT802802.1, GT839939.1, GT857087.1, GT857088.1	XM_003564788.1	Bdistachy_02
Brassica_oleracea_01	-	-	Boleracea_06
Brassica_01	-	-	Bnapus_04, Boleracea_02
Brassica_02	-	-	Bnapus_10, Brapa_07
Brassica_03	AM056981.1, BG544073.1, DK479624.1, DK492679.1, DK512724.1, DK513017.1, DK539221.1, DK572295.1, DK572589.1, EG020458.1, ES914420.1, ES924200.1, ES999572.1, EV058045.1, EV084943.1, EV108074.1, EV108336.1, EV143112.1, EV143322.1, EV175440.1, EV226407.1, EV226595.1, EV227123.1, EX029702.1, EX031006.1, EX031841.1, EX034109.1, EX038258.1, EX054449.1, EX121537.1, EX132544.1, FG562410.1, FG563283.1, FG568654.1, FG572345.1, FY419976.1, FY424953.1	-	Bnapus_23, Boleracea_03, Brapa_01
Brassica_04	-	-	Boleracea_04, Brapa_05
Brassica_05	CD820402.1, CD821532.1, CD822406.1, CN727519.1, CX189046.1, DK468071.1, DK477875.1, DK527692.1, DK537473.1, EE471039.1, EE477074.1, EE529422.1, ES994755.1, EV041828.1, EV055301.1, EV078567.1, EV108094.1, EV108378.1, EV178717.1, EV178746.1, EV178801.1, EV178837.1, EX065458.1, EX080443.1, FG554243.1, FG561185.1	-	Bnapus_07, Bnapus_20, Boleracea_08, Brapa_04

**Tip Name**	**EST**	**NT**	**Genomic**
Brassica_06	EX114048.1	-	Bnapus_17, Brapa_06
Brassica_07	EE427567.1, EE473060.1, ES926971.1, ES982475.1, EV000363.1, EV003794.1, EV057217.1, EV086590.1, EX075292.1, FG572729.1, FG576312.1	-	Boleracea_05, Brapa_08
Brassica_08	EE558593.1, EE566282.1, ES981091.1, EV043872.1, EX120531.1, FY418762.1, FY419588.1, FY423805.1, FY424593.1	-	Brapa_03

Tip Name	EST	NT	Genomic
Brassica_09	BQ791108.1, CX187879.1, CX192184.1, DK462087.1, DK462128.1, DK472869.1, DK477240.1, DK477490.1, DK478901.1, DK497331.1, DK500091.1, DK508417.1, DK508904.1, DK510642.1, DK512437.1, DK532487.1, DK536841.1, DK537090.1, DK538497.1, DK556906.1, DK559658.1, DK567975.1, DK568462.1, DK570210.1, DK572008.1, DN964240.1, DY022538.1, DY024678.1, EE419065.1, EE439150.1, EE444732.1, EE455270.1, EE459219.1, EE459601.1, EE518278.1, EE520465.1, EH429345.1, EH429349.1, EL588658.1, ES905544.1, ES911554.1, ES930251.1, EV020544.1, EV025000.1, EV039587.1, EV039598.1, EV042300.1, EV047257.1, EV058891.1, EV062511.1, EV087573.1, EV115631.1, EV115711.1, EV148223.1, EV148421.1, EV148564.1, EV148607.1, EV148835.1, EV169887.1, EV179837.1, EV179910.1, EV210347.1, EV210648.1, EV217905.1, EV218129.1, EX026280.1, EX026471.1, EX031926.1, EX034305.1, EX036983.1, EX044586.1, EX055299.1, EX086802.1, EX090579.1, EX095057.1, EX112100.1, EX112965.1, EX112983.1, EX122102.1, EX125266.1, EX134890.1, FY063751.1, FY068420.1, FY418592.1, FY420627.1, FY420650.1, FY422825.1, FY423639.1, FY425586.1, FY425608.1, FY427737.1, GR717444.1, GR727199.1, GT082220.1	-	Bnapus_06, Boleracea_07, Brapa_02

Tip Name	EST	NT	Genomic
Bryopsis_maxima_01	-	AB164319.1	-
Cajanus_cajan_01	-	-	Ccajan_01
Cajanus_cajan_02	-	-	Ccajan_03
Cajanus_cajan_03	-	-	Ccajan_04
Cajanus_cajan_04	-	-	Ccajan_06, Ccajan_07
Cajanus_cajan_05	-	-	Ccajan_09
Cajanus_cajan_06	-	-	Ccajan_10
Calystegia_sepium_01	-	AF139660.1	-
Camelina_sativa_01	-	-	Csativa_05, Csativa_11, Csativa_22
Camelina_sativa_02	-	-	Csativa_09, Csativa_20
Camelina_sativa_03	-	-	Csativa_07, Csativa_12, Csativa_23
Camelina_sativa_04	-	-	Csativa_16, Csativa_17, Csativa_19
Camelina_sativa_05	-	-	Csativa_13, Csativa_14, Csativa_18
Camelina_sativa_06	-	-	Csativa_03, Csativa_21
Camellia_sinensis_01	FS946497.1, JZ495050.1	-	-
Cannabis_sativa_01	JK493968.1	-	Csativa_02
Capsella_01	-	XM_006294762.1	Cgrandifl_01, Crubella_04
Capsella_02	-	XM_006303663.1	Cgrandifl_04, Crubella_02
Capsella_03	-	XM_006304074.1, XM_006304075.1, XM_006304190.1	Cgrandifl_05, Crubella_01
Capsella_04	-	XM_006305453.1	Cgrandifl_02, Crubella_03
Capsella_05	-	XM_006291733.1	Cgrandifl_06, Crubella_05
Capsicum_annuum_01	GD091034.1, GD099048.1, GD130204.1, GD130568.1, GD135382.1	-	-
Capsicum_annuum_02	GD053097.1, GD078635.1, GD114480.1, GO344514.1	-	-

Tip Name	EST	NT	Genomic
Capsicum_01	CA524640.1, CA525274.1, GD105480.1, GD108347.1	-	Cchinense_02
Capsicum_02	BM062630.1, BM064258.1, GD102513.1	-	Cchinense_10

**Tip Name**	**EST**	**NT**	**Genomic**
Capsicum_03	BM067364.1, CA514041.1, CA522278.1, CA522797.1, CA522802.1, CA522971.1, CA523320.1, CA524353.1, CA525501.1, CA525906.1, CA526000.1, CA526151.1, CA526252.1, CA526262.1, GD063560.1, GD064008.1, GD065163.1, GD066316.1, GD081583.1, GD081685.1, GD081824.1, GD081983.1, GD082209.1, GD082231.1, GD082315.1, GD082324.1, GD082337.1, GD082901.1, GD082983.1, GD082991.1, GD083386.1, GD083703.1, GD083788.1, GD083943.1, GD084158.1, GD084224.1, GD084533.1, GD084876.1, GD084877.1, GD085143.1, GD085252.1, GD085333.1, GD085443.1, GD085445.1, GD085453.1, GD085456.1, GD085663.1, GD085676.1, GD086011.1, GD086175.1, GD086581.1, GD086700.1, GD086708.1, GD086763.1, GD086787.1, GD086853.1, GD087019.1, GD087346.1, GD087774.1, GD087852.1, GD088290.1, GD088510.1, GD088823.1, GD089339.1, GD089441.1, GD089455.1, GD089568.1, GD089739.1, GD089906.1, GD090030.1, GD090204.1, GD090460.1, GD090606.1, GD090618.1, GD091319.1, GD091601.1, GD091987.1, GD092350.1, GD092584.1, GD092966.1, GD093107.1, GD093118.1, GD093218.1, GD093225.1, GD095430.1, GD096359.1, GD097128.1, GD097645.1, GD097755.1, GD097803.1, GD098455.1, GD110415.1, GD128419.1, GD128931.1, GD129080.1, GD129523.1	-	Cchinense_06

Tip Name	EST	NT	Genomic
Capsicum_04	CA517702.1, GD087609.1, GD091398.1, GD097925.1, GD128857.1, GD129888.1, GD132096.1, GD132368.1, GD132770.1, GD133753.1, GD133764.1	-	Cchinense_08
Capsicum_05	BM063612.1, CA514048.1, GD065126.1, GD065629.1, GD069409.1, GD087117.1, GD087166.1, GD087417.1, GD087579.1, GD088204.1, GD088755.1, GD089531.1, GD089909.1, GD090894.1, GD091246.1, GD091295.1, GD091320.1, GD091436.1, GD092002.1, GD092192.1, GD092523.1, GD092796.1, GD094442.1, GD094560.1, GD130472.1	AF168415.1	Cchinense_12
Carica_papaya_01	EX290976.1, EX294020.1	-	Cpapaya_01
Carica_papaya_02	-	-	Cpapaya_02
Carica_papaya_03	-	-	Cpapaya_03, Cpapaya_04
Carica_papaya_04	EX293834.1	-	Cpapaya_05
Carica_papaya_05	EX283022.1, EX287534.1, EX299763.1	-	Cpapaya_06
Carthamus_tinctorius_01	EL409301.1	-	-
Carthamus_tinctorius_02	EL410866.1	-	-
Carthamus_tinctorius_03	EL394361.1, EL399055.1	-	-
Carthamus_tinctorius_04	EL404327.1, EL405018.1, EL412373.1, EL511134.1	-	-
Castanea_01	GO923926.1	AB609975.1	-
Catha_edulis_01	JG724177.1, JG724380.1, JG725576.1, JG728114.1	-	-
Catha_edulis_02	JG726341.1	-	-
Centaurea_maculosa_01	EH734241.1	-	-
Centaurea_solstitialis_01	EH756434.1	-	-
Centaurea_01	EH726676.1, EH726937.1, EH728982.1, EH731390.1, EH736019.1, EH737022.1, EH737238.1, EH738989.1, EH739977.1, EH746764.1, EH754204.1, EH782627.1, EH790438.1, EH791022.1	-	-

Tip Name	EST	NT	Genomic
Centaurea_02	EH741788.1, EH746815.1, EH779170.1, EH781872.1, EH782470.1, EH783954.1, EH788668.1	-	-
Cephalotus_follicularis_01	-	AB811227.1, AB811228.1	-
Chlamydomonas_reinhardtii_01	-	XM_001691327.1	-
Cicer_arietinum_01	-	XM_004515129.1	Carietinu_04
Cicer_arietinum_02	-	XM_004510673.1	Carietinu_10
Cicer_arietinum_03	-	-	Carietinu_11
Cicer_arietinum_04	-	XM_004505964.1	Carietinu_13
Cicer_arietinum_05	-	NM_001279158.1, XM_004490009.1	Carietinu_26, Carietinu_27
Cicer_arietinum_06	-	XM_004490010.1	Carietinu_29
Cicer_arietinum_07	-	XM_004490011.1	Carietinu_30
Cicer_arietinum_08	-	XM_004486248.1	Carietinu_31
Cicer_arietinum_09	-	-	Carietinu_33
Cicer_arietinum_10	-	XM_004505328.1	Carietinu_12
Cicer_arietinum_11	-	XM_004506033.1	Carietinu_14
Cicer_arietinum_12	-	XM_004506950.1, XM_004506951.1, XM_004506952.1, XM_004506953.1	Carietinu_15
Cichorium_intybus_01	EH689540.1, EH690990.1, EH691693.1, EH694059.1, EH694971.1, EH705764.1, EH710070.1	-	-
Cichorium_01	EH675645.1, EH686758.1, EH690447.1, EH697682.1, EL362594.1	-	-
Cichorium_02	EH700926.1, EH700931.1, EH701605.1, EH703994.1, EH706515.1, EH708041.1, EH710258.1, EL368961.1	-	-
Citrullus_lanatus_01	JG701453.1, JG703248.1, JG703489.1, JG703960.1, JG704854.1, JG705223.1	-	-
Citrullus_lanatus_02	GD176302.1, GD176310.1, GD176866.1, GD177196.1, GD178968.1, GD179422.1, GD179662.1, GD180521.1	-	Clanatus_13

Tip Name	EST	NT	Genomic
Citrullus_lanatus_03	DV737378.1, DV737589.1, GD174072.1, GD174216.1, GD178242.1	-	Clanatus_14
Citrullus_lanatus_04	-	-	Clanatus_01
Citrullus_lanatus_05	-	-	Clanatus_02
Citrullus_lanatus_06	-	-	Clanatus_07
Citrullus_lanatus_07	-	-	Clanatus_08
Citrullus_lanatus_08	-	-	Clanatus_09
Citrullus_lanatus_09	-	-	Clanatus_10
Citrullus_lanatus_10	-	-	Clanatus_11
Citrus_unshiu_01	DC899732.1	-	-
Citrus_01	CF835859.1, CX044215.1, CX044216.1, CX295559.1	XM_006438634.1, XM_006438635.1, XM_006483087.1	Cclementi_02, Csinensis_01
Citrus_02	CX045273.1, CX045274.1, DC884478.1	XM_006438636.1, XM_006483086.1	Cclementi_03, Csinensis_02
Citrus_03	DC898757.1	XM_006431668.1, XM_006494311.1	Cclementi_04, Cclementi_06
Citrus_04	CB292631.1, CB292632.1, CF417379.1, CF653378.1, CF838055.1, CK936770.1, CV715706.1, CX289736.1, CX289755.1, CX293658.1, DY259885.1, EY656016.1, EY834284.1, EY840262.1, EY840708.1, EY840891.1, EY841218.1, FC927211.1	FJ917371.1, GU120096.1, GU220071.1, GU220072.1, XM_006447854.1, XM_006447855.1, XM_006469304.1	-
Cleome_hassleriana_01	-	-	Chassleri_01
Cleome_01	GR933379.1	-	Chassleri_03
Cleome_02	GR935235.1	-	Chassleri_02
Coffea_canephora_01	-	FN547910.1	-
Coffea_canephora_02	-	FN547914.1	-
Coffea_canephora_03	-	FN547915.1	-
Coffea_canephora_04	DV671894.1, DV675591.1, DV676616.1, DV680291.1, DV683019.1, DV683253.1, DV683890.1, DV711148.1, EE197299.1, EE199203.1	-	-
Coffea_canephora_05	DV663954.1, DV677885.1, DV679833.1, DV691178.1, DV691510.1, EE193267.1, GT652885.1	-	-

Tip Name	EST	NT	Genomic
Coffea_canephora_06	-	FN547912.1, FN547913.1	-
Coffea_canephora_07	-	FN547911.1	-
Coffea_canephora_08	-	FN547919.1	-
Cryptomeria_japonica_01	BW994478.1, BW997145.1, BY888545.1, BY889589.1, BY901048.1	-	-
Cryptomeria_japonica_02	BY881078.1, BY893346.1, BY899680.1, BY910815.1	-	-
Cucumis_melo_01	-	-	Cmelo_10
Cucumis_melo_02	AM715481.2, AM716051.2, AM720647.2, AM729864.1	-	Cmelo_06
Cucumis_melo_03	AM714647.2, AM723622.2, AM724941.2, AM725433.2, AM725949.2, AM728018.2, AM730911.2, AM734040.2, AM737077.2, JG499862.1	-	Cmelo_07
Cucumis_melo_04	JG508853.1, JG516100.1	-	Cmelo_11
Cucumis_sativus_01	-	XM_004148648.1, XM_004170212.1	Csativus_03
Cucumis_sativus_02	-	XM_004135406.1, XM_004155338.1	Csativus_05
Cucumis_sativus_03	-	XM_004135127.1	Csativus_04
Cucumis_sativus_04	-	XM_004137114.1	Csativus_10
Cucumis_sativus_05	-	XM_004137939.1	Csativus_02
Cucumis_sativus_06	-	XM_004147451.1	Csativus_07
Cucumis_sativus_07	-	XM_004173295.1	Csativus_06
Cucumis_sativus_08	-	XM_004147449.1, XM_004159596.1	Csativus_08
Cucumis_01	AM714094.1, AM715895.1, AM720546.2, AM721089.1, AM721148.2, AM722721.2, AM735731.2, DV632833.1, JG470782.1, JG478941.1, JG492397.1	XM_004148894.1, XM_004161486.1	Cmelo_08, Csativus_09
Cycas_rumphii_01	EX926848.1	-	-
Cynara_cardunculus_01	GE588088.1	-	-
Cynara_cardunculus_02	GE612095.1	-	-
Cynara_cardunculus_03	GE588043.1, GE589119.1, GE592853.1, GE596396.1, GE596990.1, GE600718.1	-	-
Cynodon_dactylon_01	ES295830.1	-	-
Cynodon_dactylon_02	ES300140.1	-	-

**Tip Name**	**EST**	**NT**	**Genomic**
Dactylis_glomerata_01	HO124290.1, HO149943.1, HO151735.1, HO158445.1, HO158527.1, HO159350.1, HO159616.1, HO159739.1, HO161598.1, HO161826.1, HO161958.1, HO162183.1, HO167622.1, HO168790.1, HO169123.1, HO169833.1, HO173685.1, HO179214.1, HO180515.1	-	-
Dactylis_glomerata_02	HO128658.1, HO128867.1, HO179637.1, HO179642.1	-	-
Dactylis_glomerata_03	HO139456.1, HO139912.1	-	-
Daucus_carota_01	JG754867.1	-	-
Dendrobium_nobile_01	HO190249.1, HO190276.1, HO190501.1, HO191489.1, HO191691.1, HO199220.1	-	-
Dianthus_caryophyllus_01	-	-	Dcaryophy_01
Dianthus_caryophyllus_02	-	-	Dcaryophy_03
Dianthus_caryophyllus_03	-	-	Dcaryophy_06
Dianthus_caryophyllus_04	FY395220.1, FY397286.1	-	Dcaryophy_09
Dionaea_muscipula_01	-	AB481098.1	-
Dioscorea_alata_01	HO852386.1	-	-
Dioscorea_alata_02	HO826052.1, HO852738.1	-	-
Drosera_adelae_01	-	AB211503.1, AB231461.1	-
Elaeis_guineensis_01	-	-	Eguineens_05
Elaeis_oleifera_01	-	-	Eoleifera_02
Elaeis_01	-	-	Eguineens_03, Eoleifera_01
Elaeis_02	DW248263.1, DW248694.1, EL608801.1, EL681934.1, EL681991.1, EL685224.1, EL690419.1, EL694516.1, EL694517.1, EL695346.1	AY548534.1	-
Elaeis_03	-	-	Eguineens_01, Eoleifera_04
Elymus_spicatus_01	FF342552.1, FF343227.1, FF357550.1	-	-
Elymus_spicatus_02	FF342766.1, FF342767.1, FF352931.1, FF352932.1	-	-

Tip Name	EST	NT	Genomic
Elymus_spicatus_03	FF340156.1, FF340261.1, FF340352.1, FF340416.1, FF340538.1, FF341204.1, FF342502.1, FF342503.1, FF344090.1, FF345278.1, FF347568.1, FF347762.1, FF349322.1, FF349718.1, FF350519.1, FF350834.1, FF354960.1, FF355464.1, FF355989.1, FF356914.1, FF357950.1, FF359620.1, FF362662.1, FF362934.1, FF363963.1	-	-
Ensete_ventricosum_01	-	-	Eventrico_01
Ephedra_distachya_01	JG720844.1	-	-
Eriobotrya_japonica_01	-	EU442286.1, GU384665.1	-
Eriobotrya_japonica_02	-	EU442288.1, GQ202269.4	-
Eriobotrya_japonica_03	-	EU442287.1, GU384666.1	-
Eriobotrya_japonica_04	-	EU442285.1	-
Eriobotrya_japonica_05	-	EU442289.1, GU384667.1	-
Eschscholzia_californica_01	CK752708.1, JG613014.1, JG614524.1	-	-
Eucalyptus_grandis_01	HS044775.1, HS064787.1, HS068111.1	-	Egrandis_03
Eucalyptus_01	-	-	Ecamaldul_01, Egrandis_01
Eucommia_ulmoides_01	FY902195.1, FY907790.1	-	-
Eucommia_ulmoides_02	FY897552.1, FY897614.1, FY904512.1, FY906691.1, FY908375.1, FY914839.1, FY915319.1, FY915477.1, FY915600.1, FY916245.1, FY918470.1, FY918480.1, FY920243.1, FY923987.1	-	-
Eucommia_ulmoides_03	FY908258.1, FY917770.1, FY922170.1	-	-
Euonymus_alatus_01	HS006241.1, HS251463.1	-	-
Euphorbia_esula_01	DV120817.1, DV121487.1, DV122232.1	-	-

Tip Name	EST	NT	Genomic
Euphorbia_esula_02	DV115613.1, DV117038.1, DV118409.1, DV140278.1, DV140780.1, DV144557.1	-	-
Euphorbia_esula_03	DV115838.1, DV117206.1, DV118588.1	-	-
Eutrema_salsugineum_01	-	XM_006395681.1	Esalsugin_05
Eutrema_salsugineum_02	-	XM_006415904.1	Esalsugin_02
Eutrema_salsugineum_03	-	XM_006416977.1, XM_006416978.1	Esalsugin_04
Eutrema_01	BY801883.1, BY801884.1, BY829958.1	XM_006414924.1	Esalsugin_01
Fagopyrum_01	-	AB232934.1, AB232935.1, AB232936.1	-
Festuca_arundinacea_01	DT710046.1, DT712402.1, DT712730.1, DT713107.1	-	-
Festuca_arundinacea_02	DT689815.1, DT690074.1, DT690158.1, DT690338.1	-	-
Fragaria_01	-	XM_004297250.1	Fiinumae_04, Fnipponic_05, Fnubicola_05, Fx_09
Fragaria_02	-	XM_004297251.1	Fiinumae_02, Fnipponic_06, Foriental_04
Fragaria_03	-	XM_004301714.1	Fiinumae_06, Fvesca_03, Fx_08
Fragaria_04	DV440585.1	XM_004304857.1	Fiinumae_03, Fvesca_05
Fragaria_05	EX665664.1, EX665717.1, EX665831.1, EX668189.1, EX670882.1	XM_004290057.1	Fiinumae_01, Fnipponic_08, Fnubicola_04, Fvesca_02, Fx_10
Fragaria_06	-	XM_004287659.1	Fnubicola_07, Fvesca_01
Fraxinus_excelsior_01	-	-	Fexcelsio_01
Fumaria_officinalis_01	JG658550.1	-	-
Ginkgo_biloba_01	EX932034.1	-	-
Glycine_max_01	-	XM_006577007.1	Gmax_10
Glycine_max_02	EV278085.1, EV281800.1, FK015688.1	NM_001250157.1	Gmax_18

Tip Name	EST	NT	Genomic
Glycine_max_03	BE609956.1, BU544321.1	XM_003518684.2	Gmax_08
Glycine_max_04	BI972582.1, BW661627.1, CX549006.1, CX711401.1, EV265984.1, FG988411.1, FG994523.1, FG994524.1, FK001242.1, GR850906.1, GR850907.1, GR851647.1, GR851648.1, HO017288.1	AK245022.1, NM_001248254.1, XM_003517942.2	Gmax_02, Gmax_09
Glycine_max_05	-	XM_003517941.2	Gmax_01
Glycine_max_06	-	XM_003519879.2, XM_003547972.1	Gmax_05
Glycine_max_07	BF325375.1, EV264023.1, EV267427.1, EV270351.1, EV274635.1, FG991005.1	NM_001251520.1	Gmax_17
Glycine_01	AI416638.1, BE020073.1, BE191140.1, BE611605.1, BG510292.1, BG726388.1, BG882209.1, BI469866.1, BI787850.1, BQ628757.1, BQ742455.1, BU090131.1, BU091537.1, BW652661.1, BW669587.1, CA799030.1, CA801284.1, DB960292.1, EV272077.1, EV278099.1, FK008542.1, FK008543.1, GR826078.1, GR845837.1, HO016466.1, HO019657.1	AK245443.1, XM_003528758.2, XM_003548506.2	Gmax_13
Glycine_02	BG156052.1, BI425210.1, BI470540.1, BM520744.1, BM523270.1, BM523369.1, BM526581.1, BM526584.1, BQ612399.1, BU081349.1, CA852912.1, EV279232.1, EV279385.1, EV281645.1, EV282053.1, EV282744.1, FK017953.1, FK024571.1, FK024572.1, HO212305.1	BT099269.1, NM_001248243.1, XM_003518068.2, XM_003518071.2	Gmax_04, Gmax_06, Gmax_07
Gossypium_arboreum_01	-	-	Garboreum_04
Gossypium_arboreum_02	-	-	Garboreum_10, Garboreum_11
Gossypium_raimondii_01	-	-	Graimondi_08
Gossypium_raimondii_02	-	-	Graimondi_17

**Tip Name**	**EST**	**NT**	**Genomic**
Gossypium_01	DR452451.1, DT545608.1, DT545724.1, DT545780.1, DT545791.1, DW505462.1	-	Garboreum_07, Graimondi_02
Gossypium_02	DR462086.1, DW500330.1, DW500331.1	-	Garboreum_08, Graimondi_04
Gossypium_03	AI729386.1, AI729649.1, BM359116.1, BQ405390.1, CO099187.1, CO105024.1, CO112780.1, CO112781.1, DR458181.1, DT552848.1, DT557061.1, DT558244.1, DT562773.1, DT563576.1, DT574569.1, DW224232.1, DW224741.1, DW511786.1, DW516456.1, ES793056.1, ES799270.1, ES799914.1, ES826089.1, ES846751.1, EV490351.1, EV490379.1, EV492392.1, EV495145.1, EV496829.1, HO091693.1, JG842523.1, JG843027.1, JG847864.1, JK801605.1	-	Garboreum_06, Graimondi_01
Gossypium_04	-	-	Garboreum_09, Graimondi_03
Gossypium_05	DT463266.1, DT463267.1, ES815921.1, GR926562.1, GR926656.1, GR929575.1, GR929579.1, HO096514.1, HO105898.1, HO109133.1	-	Graimondi_05
Gossypium_06	-	-	Garboreum_05, Graimondi_19
Grimmia_pilifera_01	GR307381.1, GR307673.1	-	-
Guizotia_abyssinica_01	GE564326.1, GE569344.1	-	-
Helianthus_01	EE638765.1, EE639184.1, EE643073.1, EE650763.1, EL459416.1	-	-
Helianthus_02	DY953463.1, GE517775.1	-	-

Tip Name	EST	NT	Genomic
Helianthus_03	DY905680.1, DY906289.1, DY909029.1, DY909046.1, DY911865.1, DY914640.1, DY914727.1, DY914914.1, DY915628.1, DY915879.1, DY916036.1, DY916836.1, DY917049.1, DY917809.1, DY920355.1, DY922049.1, DY922811.1, DY923179.1, DY923304.1, DY937190.1, DY940122.1, DY940421.1, DY941964.1, DY942592.1, DY942695.1, DY942890.1, DY943192.1, DY943319.1, DY943345.1, DY943457.1, DY943611.1, DY944871.1, DY944915.1, DY945230.1, DY945268.1, DY945493.1, DY945727.1, DY945798.1, DY945945.1, DY945983.1, DY946033.1, DY946127.1, DY946639.1, DY947208.1, DY947209.1, DY947402.1, DY947543.1, DY948326.1, DY948866.1, DY949250.1, DY949412.1, DY949572.1, DY949696.1, DY949753.1, DY950115.1, DY950132.1, DY950188.1, DY950725.1, DY950748.1, DY951502.1, DY951593.1, DY951917.1, DY951964.1, DY952133.1, DY952398.1, DY952876.1, DY953201.1, DY953277.1, EE610720.1, EE613962.1, EE620497.1, EL427482.1, EL428306.1, EL432766.1, EL435265.1, EL440723.1, EL463845.1, EL464359.1, EL464453.1, EL465319.1, EL470296.1, EL471271.1, GE506734.1	-	-

Tip Name	EST	NT	Genomic
Helianthus_04	DY911059.1, DY915241.1, DY917269.1, DY920159.1, DY921847.1, DY924062.1, DY925079.1, DY942884.1, DY944462.1, DY949620.1, EL412580.1, EL425553.1, EL427543.1, EL428233.1, EL434588.1, EL447387.1, EL460997.1, EL461428.1, EL461551.1, EL466382.1, EL487616.1, EL513003.1, EL513130.1, EL513233.1	-	-
Helianthus_05	DY914656.1, DY914842.1, DY917823.1, DY920334.1, DY943979.1, DY945420.1, EE608813.1, EE623589.1, EE650068.1, EL428038.1, EL460101.1, EL462197.1, EL488096.1, EL513859.1, GE518687.1, GE518688.1, GE518767.1, GE518768.1, GE519329.1	-	-
Helianthus_06	DY922217.1, DY945231.1, DY953056.1, DY953259.1, EE621342.1	-	-
Helianthus_07	BQ914410.1, DY917820.1, DY921407.1, DY923716.1, DY944864.1, DY946241.1, EE642837.1, EE646434.1, EE656059.1, EL484816.1, GE500598.1	-	-
Hevea_brasiliensis_01	-	-	Hbrasilie_24
Hordeum_pubiflorum_01	-	-	Hpubiflor_06
Hordeum_pubiflorum_02	-	-	Hpubiflor_07
Hordeum_vulgare_01	AL506966.1, BE422325.1, BG368983.1, BJ479061.1, BY846022.1, BY846428.1, DK587721.1, DK605033.1, DK612942.1, DK618077.1, DK620621.1, DK623310.1, DK624080.1, DK625545.1, DK626587.1, DK638568.1, DK647671.1, DK659886.1, DK687025.1, DK722767.1	AK354480.1, AK362017.1, AK364745.1	-

Tip Name	EST	NT	Genomic
Hordeum_vulgare_02	-	-	Hvulgare_03
Hordeum_vulgare_03	-	-	Hvulgare_04
Hordeum_vulgare_04	BE601973.2, BF623030.2, BF625343.2	AK250005.1, AK252681.1, AY120886.1	-
Hordeum_01	DK802410.1	AK365286.1	Hpubiflor_05, Hvulgare_09
Hordeum_02	BI949346.1, BM816162.1, BM816163.1, BQ740129.1, DK587671.1, DK601711.1, DK605632.1, DK613176.1, DK666066.1, DK717260.1, DK721335.1, DK769714.1, DK866239.1, GH224721.1	AF000940.1, AK368782.1	Hpubiflor_09, Hvulgare_12
Hordeum_03	-	AF182197.1, AK250669.1	Hpubiflor_02, Hvulgare_05
Hordeum_04	AJ433177.1, AJ464321.1, AL504955.1, AL505169.1, AL505254.1, AV914669.1, AV932837.1, AV932845.1, AV933847.1, AV933848.1, AV935472.1, AV936080.1, AV941745.1, BE060118.2, BF253875.2, BF254391.2, BF255157.2, BF255271.2, BF255408.2, BF618612.2, BG343116.2, BI779336.2, BJ544303.1, BJ544602.1, BJ545766.1, BJ546077.1, BJ547671.1, BJ547993.1, BM099203.2, BM373545.2, BQ462174.1, BQ766723.1, BQ767126.1, BU968813.1, BY846554.1, BY847038.1, CA020561.1, CA021585.1, CA027459.1, CB883496.1, CD054807.1, CK125917.1, CK567638.1, DK587056.1, DK611253.1, DK614427.1, DK640936.1, DK770817.1, DK793051.1, FD518947.1, GH218544.1, GH218545.1	AF000939.1, AK251876.1	Hpubiflor_04, Hvulgare_06

Tip Name	EST	NT	Genomic
Hordeum_05	BF627174.2, BG343833.1, BI960324.1, BU998770.1, BY842839.1, BY849333.1, CA024441.1, CB869630.1, DK586850.1, DK593386.1, DK593516.1, DK597625.1, DK624337.1, DK632743.1, DK647049.1, DK647566.1, DK739959.1, DK741352.1, DK743765.1	AK354377.1, AK355768.1, AK364534.1	Hpubiflor_01, Hvulgare_10
Humulus_lupulus_01	EX515510.1, GD249872.1, GD250704.1	-	-
Humulus_lupulus_02	EX517546.1, EX520643.1	-	-
Huperzia_serrata_01	GO248796.1, GO248859.1, GO912362.1, GO914242.1	-	-
Ipomoea_batatas_01	EE875665.1	-	-
Ipomoea_batatas_02	EE882831.1, EE883036.1, EE883141.1	-	-
Ipomoea_batatas_03	EE876702.1, JG697716.1, JG697814.1, JG697924.1, JG698021.1, JG698103.1	-	-
Ipomoea_batatas_04	EE877251.1, EE877662.1	-	-
Ipomoea_nil_01	CJ754416.1	-	-
Ipomoea_nil_02	BJ560140.1, BJ568552.1, CJ743080.1, CJ746892.1, CJ749639.1, CJ750833.1, CJ756606.1	-	-
Ipomoea_nil_03	BJ556286.1, BJ572130.1	-	-
Ipomopsis_aggregata_01	DT575316.1	-	-
Iris_fulva_01	EX954717.1, EX955804.1, EX955993.1	-	-
Jatropha_curcas_01	GT978984.1, GT980119.1	-	Jcurcas_01
Jatropha_curcas_02	-	-	Jcurcas_02
Jatropha_curcas_03	GT978644.1, GW616373.1, GW877769.1	-	Jcurcas_03
Krascheninnikovia_lanata_01	HS587315.1	-	-
Lactuca_sativa_01	-	-	Lsativa_03
Lactuca_sativa_02	-	-	Lsativa_04

Tip Name	EST	NT	Genomic
Lactuca_01	BQ848018.1, BQ856204.1, BQ857299.1, DW056114.1, DW062454.1, DW062929.1, DW096090.1, DW097057.1, DW097430.1, DW125237.1, DY977363.1, DY977411.1, DY979305.1, DY979479.1, DY984956.1	-	-
Lactuca_02	DW084365.1, DW084652.1	-	Lsativa_05
Lactuca_03	BQ991918.1, DW110112.1, DW124816.1, DW145116.1, DW150085.1, DW156183.1, DW157791.1, DW165257.1, DY983286.1	-	-
Lagenaria_siceraria_01	-	-	Lsicerari_12
Lathyrus_odoratus_01	GO315198.1, GO316275.1, GO317387.1, GO318179.1, GO318300.1, GO318683.1, GO319469.1, GO320798.1, GO321081.1, GO322355.1, GO322431.1	-	-
Leavenworthia_alabamica_01	-	-	Lalabamic_01
Leavenworthia_alabamica_02	-	-	Lalabamic_02
Leavenworthia_alabamica_03	-	-	Lalabamic_03
Leavenworthia_alabamica_04	-	-	Lalabamic_04
Leavenworthia_alabamica_05	-	-	Lalabamic_06
Leersia_perrieri_01	-	-	Lperrieri_02
Leersia_perrieri_02	-	-	Lperrieri_03
Leersia_perrieri_03	-	-	Lperrieri_04
Leersia_perrieri_04	-	-	Lperrieri_06
Leersia_perrieri_05	-	-	Lperrieri_07
Leersia_perrieri_06	-	-	Lperrieri_08
Leersia_perrieri_07	-	-	Lperrieri_09
Limnanthes_alba_01	FD654739.1	-	-
Linum_usitatissimum_01	JG218276.1, JG250847.1	-	Lusitatis_02
Linum_usitatissimum_02	-	-	Lusitatis_01
Linum_usitatissimum_03	-	-	Lusitatis_03
Liriodendron_tulipifera_01	FD495669.1	-	-
Liriodendron_tulipifera_02	DT584387.1, FD491845.1, FD493328.1, FD495379.1, FD495878.1	-	-

Tip Name	EST	NT	Genomic
Liriodendron_tulipifera_03	DT582846.1, DT596269.1, FD492742.1, FD493759.1, FD494098.1, FD495069.1, FD497605.1, FD497720.1, FD498480.1, FD501848.1	-	-
Lotus_japonicus_01	GO022452.1, GO028056.1	BT137426.1, BT148442.1	-
Lotus_japonicus_02	FS318593.1, FS320654.1, FS321695.1, FS326312.1, FS327292.1, FS333298.1, FS334339.1, FS335812.1, FS345534.1, FS345821.1, FS347352.1, FS348267.1, FS352646.1, FS353627.1, FS359827.1, FS362292.1, GO009251.1	-	-
Lotus_japonicus_03	BI419984.1, BI420700.1, BP041087.1, BW595750.1, CB828705.1, DC595428.1, FS336467.1, GO031730.1, GO032406.1, GO038501.1	BT135211.1, BT142812.1	-
Lotus_japonicus_04	GO024523.1	BT141961.1	-
Luffa_aegyptiaca_01	-	D64011.1	-
Luffa_aegyptiaca_02	-	D64012.1	-
Lupinus_angustifolius_01	-	-	Langustif_01
Lupinus_angustifolius_02	-	-	Langustif_02
Lupinus_angustifolius_03	-	-	Langustif_04
Lupinus_angustifolius_04	-	-	Langustif_05
Lupinus_angustifolius_05	-	-	Langustif_06
Lupinus_angustifolius_06	-	-	Langustif_08
Malus_angustifolia_01	-	FJ535239.1	-
Malus_domestica_01	-	-	Mdomestic_05
Malus_domestica_02	-	-	Mdomestic_07
Malus_domestica_03	-	-	Mdomestic_18
Malus_kansuensis_01	-	FJ535242.1	-
Malus_sieversii_01	-	AB540121.1	-
Malus_spectabilis_01	-	FJ943266.1	-
Malus_spectabilis_02	-	FJ943268.1	-
Malus_spectabilis_03	-	FJ943272.1	-
Malus_transitoria_01	-	AB035928.1	-
Malus_01	-	FJ535243.1, FJ943270.1	-

Tip Name	EST	NT	Genomic
Malus_02	-	FJ535241.1, FJ943264.1	-
Malus_03	-	AB096138.1, FJ535240.1	-
Medicago_truncatula_01	GT142477.1	-	-
Medicago_truncatula_02	-	XM_003620719.1	Mtruncatu_16
Medicago_truncatula_03	-	XM_003624084.1	-
Medicago_truncatula_04	-	XM_003637773.1	Mtruncatu_21
Medicago_truncatula_05	BE319199.2, CX526757.1, CX539524.1	XM_003613749.1	Mtruncatu_10
Medicago_truncatula_06	-	BT136026.1, XM_003613754.1	Mtruncatu_12
Medicago_truncatula_07	BG646994.1, BG647157.1	XM_003613752.1	Mtruncatu_11
Medicago_truncatula_08	-	XM_003594090.1	Mtruncatu_02
Medicago_truncatula_09	GE345676.1, GE350597.1	XM_003599193.1, XM_003599194.1	Mtruncatu_03, Mtruncatu_04
Medicago_truncatula_10	AJ498015.1	-	Mtruncatu_05
Medicago_truncatula_11	-	XM_003613741.1	Mtruncatu_07
Medicago_truncatula_12	AW775298.1, BF519171.1, BF645898.1, BG455183.1, BI263423.1, BQ165730.1, CA918628.1, CF067881.1, DW016055.1, DW016749.1, EY476689.1, GT141725.1	BT148860.1, XM_003613743.1	Mtruncatu_08
Medicago_truncatula_13	AW684365.1, BE202582.1, BE239743.1, BG451455.1, CA922286.1, EY478157.1, GT139697.1, GT139923.1, GT140673.1, GT144145.1, GT144358.1	BT133720.1, XM_003613747.1	Mtruncatu_09
Medicago_truncatula_14	-	XM_003616934.1	Mtruncatu_13
Medicago_01	AJ503533.1, AL371801.1, AL376111.1, AW775566.1, AW775880.1, AW980478.1, BE124916.1, BF518644.1, BF520401.1, BF649134.1, BG454963.1, BG455225.1, BG455347.1, BG580140.1, BG581199.1, BG581672.1, BI263919.1, CF069167.1, CX535430.1, DY615831.1, EV255202.1, EV256367.1, EV262558.1, EX524557.1, GE347841.1	BT148419.1, XM_003637193.1	Mtruncatu_20

**Tip Name**	**EST**	**NT**	**Genomic**
Mimulus_guttatus_01	GO946429.1, GR008377.1, GR038524.1, GR038525.1, GR133402.1, GR144373.1, GR144374.1	-	Mguttatus_01
Mimulus_lewisii_01	GR203325.1, GR208676.1	-	-
Mimulus_01	GO979765.1, GR084192.1, GR134009.1, GR134010.1, GR184732.1, GR189852.1	-	Mguttatus_04
Mimulus_02	GO990564.1, GO990565.1, GR070495.1, GR070496.1, GR086807.1, GR086808.1, GR088328.1, GR088329.1	-	Mguttatus_02
Mimulus_03	GO984157.1, GR027606.1, GR040389.1, GR066153.1, GR068386.1, GR090022.1, GR118923.1, GR126556.1, GR128121.1	-	-
Misopates_orontium_01	-	AY013906.1, AY013907.1	-
Morus_notabilis_01	-	-	Mnotabili_01
Morus_notabilis_02	-	-	Mnotabili_02
Musa_01	FF560739.1, FL649419.1, FL651345.1	-	Macuminat_01, Mbalbisia_01
Nelumbo_nucifera_01	-	M83668.1	-
Nelumbo_nucifera_02	-	-	Nnucifera_04
Neolamarckia_cadamba_01	JZ545113.1	-	-
Nepenthes_bicalcarata_01	-	AB872469.1	-
Nicotiana_alata_01	-	U45957.1	-
Nicotiana_alata_02	-	U66427.1	-
Nicotiana_alata_03	-	D63888.1	-
Nicotiana_alata_04	-	U08860.1	-
Nicotiana_alata_05	-	U08861.1	-
Nicotiana_alata_06	-	D63887.1, GQ850520.1	-
Nicotiana_alata_07	-	U13255.1, U45959.1	-
Nicotiana_benthamiana_01	-	-	Nbenthami_07
Nicotiana_otophora_01	-	-	Notophora_04
Nicotiana_sylvestris_01	-	AJ002296.1	-
Nicotiana_sylvestris_02	-	-	Nsylvestr_05
Nicotiana_sylvestris_03	-	-	Nsylvestr_12
Nicotiana_tomentosiformis_01	-	-	Ntomentos_04

Tip Name	EST	NT	Genomic
Nicotiana_01	-	AB032257.1, AB112026.1	Nbenthami_05, Nbenthami_12, Notophora_02, Nsylvestr_06
Nicotiana_02	-	AB032256.1, AB112027.1	Nbenthami_02, Nbenthami_06, Notophora_05, Nsylvestr_08, Ntomentos_03
Nicotiana_03	-	AB112028.1	Notophora_01, Ntomentos_05
Nicotiana_04	-	U13256.1	Nbenthami_04, Nbenthami_11, Nsylvestr_07
Oryza_brachyantha_01	-	XM_006657930.1	-
Oryza_brachyantha_02	-	XM_006659370.1	Obrachyan_04
Oryza_brachyantha_03	-	XM_006660835.1	-
Oryza_brachyantha_04	-	XM_006660836.1	-
Oryza_punctata_01	-	-	Opunctata_03
Oryza_punctata_02	-	-	Opunctata_04
Oryza_punctata_03	-	-	Opunctata_06
Oryza_01	AU163822.1, BI305383.1, BI306312.1, CB686024.1, CB965180.1, CB967070.1, CF984156.1, CF984462.1, CF984875.1, CF987123.1, CF987585.1, CF987637.1, CF988078.1, CF988214.1, CF989293.1, CF989297.1, CF989924.1, CF990398.1, CF991233.1, CF991547.1, CF991582.1, CF991946.1, CF992209.1, CF992291.1, CF993377.1, CF993409.1, CF993555.1, CI164384.1, CI215334.1, CI252890.1, CI641995.1, CK076160.1, CK081222.1, CT848079.1, CX102339.1	AB052842.1, AB052843.1, AB052844.1, AK058502.1, AK059802.1, NM_001068412.1	Obarthii_03, Omeridion_03, Onivara_04, Osativa_06
Oryza_02	AU164607.1, CI005327.1, CT862596.1, CV723345.1	AK104512.1, CT830173.1, CT830174.1, NM_001066891.1	Oglaberri_04, Oglumipat_06, Omeridion_05, Onivara_06, Osativa_05

**Tip Name**	**EST**	**NT**	**Genomic**
Oryza_03	CA763847.2, CB660432.1, CI048175.1, CI063712.1, CI312327.1, CI563863.1, CK073765.1, CK083779.1, CX117118.1, EX450416.1	AK061505.1, AK065055.1, AK100403.1, AK105061.1, AK121862.1, NM_001051621.1	Oglaberri_01, Omeridion_07, Opunctata_07, Osativa_01
Oryza_04	AU092918.1, CA763095.1, CI010113.1, CI685069.1, CR290472.1	AK106167.1, NM_001051622.1	Oglumipat_08, Omeridion_08, Opunctata_08, Osativa_02
Oryza_05	-	-	Oglumipat_04, Osativa_03
Oryza_06	-	-	Onivara_01, Osativa_04
Oryza_07	-	AF439449.1, AK098949.1, AK104221.1, AK104454.1, AK104509.1, AK104553.1, AK104599.1, AK104888.1, AK119186.1, AY056038.1, AY061961.1, CT833414.1, EF576382.1, EF576436.1, NM_001070328.1	Oglumipat_01, Omeridion_01, Opunctata_02, Osativa_07
Oryza_08	-	AK059757.1, AK061442.1, AK104237.1, AK104261.1, AK104263.1, AK104390.1, AK104456.1, AK104648.1, AK119171.1, CT834731.1, EF575920.1, NM_001070329.2	Oglumipat_02, Omeridion_06, Onivara_03, Osativa_08
Panax_ginseng_01	DV553990.1, DV555270.1, DV555906.1, JK985713.1, JK987084.1, JK987317.1	-	-

**Tip Name**	**EST**	**NT**	**Genomic**
Panax_ginseng_02	DV555518.1, JK985185.1	-	-
Panax_ginseng_03	CN846941.1, CN847550.1, CN848547.1, HS076088.1	-	-
Panax_ginseng_04	DV553395.1, DV553439.1, DV553563.1, DV553745.1, DV553775.1, DV553866.1, DV553887.1, DV554370.1, DV554371.1, DV554400.1, DV554660.1, DV554689.1, DV554719.1, DV554785.1, DV554884.1, DV555013.1, DV555065.1, DV555086.1, DV555499.1, DV555580.1, DV555607.1, DV555640.1, DV555740.1, DV555824.1, DV555872.1, DV555951.1, DV556293.1, DV556344.1, JK988361.1	-	-
Panax_quinquefolius_01	GR872250.1	-	-
Panax_01	-	AY496964.1, KC751542.1	-
Panicum_hallii_01	-	-	Phallii_05

Tip Name	EST	NT	Genomic
Panicum_virgatum_01	FL722616.1, FL736710.1, FL775130.1, FL775131.1, FL781606.1, FL781607.1, FL829273.1, FL829274.1, FL832716.1, FL832717.1, FL844428.1, FL844429.1, FL848408.1, FL886490.1, FL886491.1, FL898203.1, FL898204.1, FL951427.1, GD015858.1, GD015859.1, GD020714.1, GD030401.1, GD030402.1, GD044834.1, HO244454.1, HO270120.1, HO272688.1, HO272689.1, HO283191.1, HO326434.1, HO331041.1, HO343647.1, HO343648.1, HO346108.1, HO346109.1, JG775690.1, JG775691.1, JG784556.1, JG784557.1, JG793312.1, JG793313.1, JG859480.1, JG867799.1, JG872678.1, JG874736.1, JG887259.1, JG896569.1, JG915647.1, JG934394.1, JG968359.1	-	Pvirgatum_11, Pvirgatum_16
Panicum_virgatum_02	-	-	Pvirgatum_01
Panicum_virgatum_03	-	-	Pvirgatum_03, Pvirgatum_05
Panicum_virgatum_04	FE620023.1, FE620024.1	-	Pvirgatum_06
Panicum_virgatum_05	-	-	Pvirgatum_13
Panicum_virgatum_06	-	-	Pvirgatum_14
Panicum_01	DN145948.1, DN151038.1, DN151646.1, FE597622.1, FE603868.1, FE610776.1, FE610777.1, FE612060.1, FE613496.1, FE613497.1, FE614762.1, FE614763.1, FE620179.1, FE620180.1, FL834015.1, FL834016.1, FL887203.1, FL887204.1, FL887670.1, FL887671.1, FL887791.1, FL887792.1, FL892515.1, FL892516.1	-	Phallii_01, Pvirgatum_08

Tip Name	EST	NT	Genomic
Panicum_02	FL772680.1, FL784303.1, FL791564.1	-	Phallii_06, Pvirgatum_04, Pvirgatum_10
Papaver_bracteatum_01	JG635005.1	-	-
Petunia_axillaris_01	-	AF239907.1	-
Petunia_axillaris_02	-	AF239908.1	-
Petunia_axillaris_03	FN014180.1	-	-
Petunia_axillaris_04	-	AY180049.1, AY180050.1	-
Petunia_integrifolia_01	-	M67990.2	-
Petunia_integrifolia_02	-	AF301533.1	-
Petunia_integrifolia_03	-	AB094599.1, M67991.2	-
Petunia_x_hybrida_01	-	GQ465918.1	-
Petunia_x_hybrida_02	-	GQ465919.1	-
Petunia_x_hybrida_03	-	GQ465920.1	-
Petunia_01	-	AB094600.1, AF239910.1	-
Phalaenopsis_equestris_01	CB033285.1	-	-
Phaseolus_vulgaris_01	-	XM_007147242.1	Pvulgaris_10
Phaseolus_vulgaris_02	-	XM_007147244.1	Pvulgaris_12
Phaseolus_vulgaris_03	-	XM_007151438.1	Pvulgaris_08
Phaseolus_vulgaris_04	-	XM_007157555.1	Pvulgaris_02
Phaseolus_vulgaris_05	-	XM_007158588.1	Pvulgaris_03
Phaseolus_vulgaris_06	GW889193.1	XM_007147243.1	Pvulgaris_11
Phaseolus_vulgaris_07	-	-	Pvulgaris_06
Phaseolus_vulgaris_08	-	XM_007151580.1	Pvulgaris_09
Phaseolus_01	CA914129.1, CA914130.1, EX303659.1, EX304583.1, HS104286.1	KF033466.1, XM_007157554.1	Pvulgaris_01
Phaseolus_02	FE677978.1, FE677979.1, FE677980.1, FE677981.1, FE677982.1, HO781084.1, JK040411.1	XM_007135147.1	Pvulgaris_13
Phoenix_dactylifera_01	-	XM_008804374.1	Pdactylif_01
Phoenix_dactylifera_02	-	XM_008792751.1	Pdactylif_02
Phoenix_dactylifera_03	-	XM_008785763.1	Pdactylif_04
Phoenix_dactylifera_04	-	XM_008790020.1	-
Phyllostachys_edulis_01	-	FP101767.1	-
Phyllostachys_edulis_02	-	FP098974.1	-
Phyllostachys_edulis_03	-	FP099346.1	-
Phyllostachys_edulis_04	-	FP099555.1	-

Tip Name	EST	NT	Genomic
Phyllostachys_edulis_05	-	FP093065.1, FP097450.1	Pedulis_09
Physcomitrella_patens_01	BJ167003.1, BJ198719.1, BJ199181.1, BJ595980.1, BJ605035.1, BJ605545.1, BJ943993.1, BJ944709.1, BJ948737.1, BJ954718.1, BJ955485.1, BJ959631.1, BJ969681.1, BJ977964.1, BQ827204.1, BU052467.1, BY961703.1, DC902237.1, DC904653.1, DC910592.1, DC911518.1, DC918545.1, DC924415.1, DC925313.1, DC931855.1, DC939456.1, DC943336.1, DC946362.1, DC953048.1, FC402811.1, FC402812.1, FC423007.1, FC423008.1, FC431513.1, FC431514.1, FC454378.1, FC454379.1	XM_001763089.1	Ppatens_01
Physcomitrella_patens_02	BJ182277.1, BJ183696.1, BJ584821.1, BJ587210.1, BJ588520.1, BJ588996.1, BJ590470.1, BJ594154.1, BJ959425.1, BJ968870.1, BJ969462.1, BJ977765.1, BQ041844.1, DC903250.1, DC911807.1, DC917158.1, DC925594.1	XM_001783253.1	Ppatens_02
Picea_abies_01	-	-	Pabies_16
Picea_glauca_01	CK444703.2, CO250761.1, DV993604.1, GE476662.1	BT101223.1	-
Picea_glauca_02	DV994762.1, EX387059.1, EX387106.1, EX387436.1, EX417855.1, GE477570.1	BT115923.1	-
Picea_01	EX322444.1, EX322815.1	BT105140.1	Pabies_04
Picea_02	CO476294.2, DV994274.1, EX386424.1, EX386654.1, EX386794.1, EX386828.1, EX387019.1, EX387082.1, EX390207.1, EX416526.1, EX437355.1, EX440507.1, GE475988.1	BT101085.1	Pabies_19

Tip Name	EST	NT	Genomic
Picea_03	CO224322.1, CO242486.1, DR548004.1, DR551667.1, DR552860.1, DR554326.1, DR571192.1, DV981147.2, DV982018.2, EX371459.1, EX371685.1, EX373310.1, EX381499.1, EX381841.1, EX427423.1, FD736018.1	BT102207.1	-
Pinus_contorta_01	GT233987.1, GT266473.1	-	-
Pinus_taeda_01	DR117606.1, DR117675.1, DR119280.1, DR119366.1	-	Ptaeda_11
Pinus_taeda_02	-	-	Ptaeda_03
Pinus_taeda_03	-	-	Ptaeda_06
Pinus_taeda_04	-	-	Ptaeda_07
Pinus_taeda_05	-	-	Ptaeda_10
Pinus_taeda_06	-	-	Ptaeda_16
Pinus_01	AI812905.1, BF186438.1, BF777643.1, BQ701198.1, BX253470.1, CT574625.1, FE523476.1, GT261220.1, GT261353.1, GT261711.1, GT264781.1, GW768231.1, GW768590.1	FN257073.1	-
Pinus_02	DR023234.1, DR685771.1, DT633007.1, GW757016.1, GW763726.1, GW770303.1	-	Ptaeda_27
Pinus_03	CO361681.1, CO361761.1, DR178219.1, GT248524.1, GT248868.1, GT260231.1, GT260565.1, GW762256.1, GW762578.1	-	Ptaeda_15, Ptaeda_17
Pistacia_vera_01	GH271766.1	-	-
Pisum_sativum_01	-	Y11824.1	-
Populus_euphratica_01	-	-	Peuphrati_01, Peuphrati_07
Populus_grandidentata_01	-	-	Pgrandide_07
Populus_grandidentata_02	-	-	Pgrandide_08
Populus_tremula_01	-	-	Ptremula_09
Populus_01	-	-	Pgrandide_04, Ptremuloi_01

Tip Name	EST	NT	Genomic
Populus_02	AJ777201.1, BU820598.1, CA933881.1, CA934198.1, CV252907.1, DB886016.1, DB887436.1, DT495581.1, DT500664.1	EF144433.1, XM_002321192.2	Peuphrati_05, Pgrandide_05, Ptremula_03, Ptremuloi_03, Ptrichoca_06
Populus_03	BU869396.1, BU881802.1, DN485375.1, DN494843.1	XM_002311267.2, XM_002316099.2	Peuphrati_02, Peuphrati_10, Pgrandide_06, Ptremula_08, Ptremuloi_08, Ptrichoca_02
Populus_04	-	XM_002316100.1	Peuphrati_08, Pgrandide_03, Ptremula_04, Ptrichoca_04
Populus_05	DB879124.1, DB901722.1	XM_002311266.2	Peuphrati_09, Pgrandide_01, Ptremula_07, Ptremuloi_07, Ptrichoca_01
Populus_06	-	XM_002319864.2, XM_006376453.1	Peuphrati_03, Peuphrati_04, Ptrichoca_05
Prunus_armeniaca_01	-	AY587561.1	-
Prunus_avium_01	-	AJ298314.1	-
Prunus_avium_02	-	EU035974.1	-
Prunus_avium_03	-	AB096918.1	-
Prunus_avium_04	-	AB028154.1, AJ298313.1	-
Prunus_avium_05	-	AB028153.1, AJ298310.1	-
Prunus_avium_06	-	AB010305.1, AJ298315.1	-
Prunus_avium_07	-	AB010306.1, AJ298312.1	-
Prunus_avium_08	-	AB010304.1, AJ298311.1	-
Prunus_cerasus_01	-	EU042128.1	-
Prunus_cerasus_02	-	DQ241590.1	-
Prunus_dulcis_01	-	AB011471.1, AB481108.1	-
Prunus_dulcis_02	-	AY291117.1	-
Prunus_dulcis_03	-	AB011470.1	-
Prunus_dulcis_04	-	DQ093825.1	-

Tip Name	EST	NT	Genomic
Prunus_dulcis_05	-	DQ157873.1	-
Prunus_dulcis_06	-	AB026836.1	-
Prunus_dulcis_07	-	AB011469.1, DQ157874.1	-
Prunus_mume_01	-	EU020123.1	-
Prunus_mume_02	-	AB101437.1	-
Prunus_mume_03	-	EU020118.1, EU020122.1	-
Prunus_mume_04	-	AB047100.1, AB101438.1	-
Prunus_persica_01	-	AB537563.1	-
Prunus_persica_02	-	AB537565.1	-
Prunus_pseudocerasus_01	-	EU073939.1	-
Prunus_pseudocerasus_02	-	EU253960.1	-
Prunus_pseudocerasus_03	-	FJ543097.1	-
Prunus_pseudocerasus_04	-	FJ543099.1	-
Prunus_pseudocerasus_05	-	FJ628598.1, HQ913635.1	-
Prunus_pseudocerasus_06	-	EU073938.1, EU253959.1	-
Prunus_salicina_01	-	AB084102.1	-
Prunus_salicina_02	-	AB026982.1	-
Prunus_salicina_03	-	DQ512911.1	-
Prunus_salicina_04	-	DQ512912.1	-
Prunus_salicina_05	-	DQ512913.1	-
Prunus_salicina_06	-	DQ512914.1	-
Prunus_webbii_01	-	DQ993661.1	-
Prunus_01	-	AF227522.1, XM_007223857.1	Pmume_06, Ppersica_02
Prunus_02	-	XM_007209513.1	Pmume_02, Ppersica_04
Prunus_03	BU045659.1, FC861706.1	XM_007201249.1	Pmume_05, Ppersica_06
Prunus_04	-	AB084103.1, DQ157875.1	-
Prunus_05	-	AB092644.1, AB101439.1, EU253962.1	-
Prunus_06	-	AB026981.1, DQ099895.1, XM_007207516.1	Ppersica_05
Prunus_07	-	DQ157876.1, EU253961.1	-

Tip Name	EST	NT	Genomic
Prunus_08	GR410459.1	AF202030.1, XM_007223859.1	Pmume_07, Ppersica_01
Punica_granatum_01	JZ123328.1	-	-
Pyrus_communis_02	-	AB258360.1	-
Pyrus_communis_03	-	AB731592.1, EU855799.1, KF500092.1, KF500093.1, KF500094.1, KF500095.1	-
Pyrus_communis_04	-	KF500082.1	-
Pyrus_communis_05	-	KF500089.1	-
Pyrus_communis_06	-	KF500069.1, KF500070.1, KF500071.1, KF500072.1, KF500090.1	-
Pyrus_communis_07	-	KF500091.1	-
Pyrus_communis_08	-	AB236432.1, KF500073.1, KF500074.1, KF500075.1	-
Pyrus_communis_09	-	KF500087.1, KF500088.1	-
Pyrus_communis_10	-	KF500083.1, KF500084.1, KF500085.1, KF500086.1	-
Pyrus_communis_11	-	KF500079.1, KF500080.1, KF500081.1	-
Pyrus_pyrifolia_01	-	D49527.1	-
Pyrus_pyrifolia_02	-	AB002139.1, AB014072.1, D49528.1	-
Pyrus_pyrifolia_03	-	AB002142.1	-
Pyrus_pyrifolia_04	-	AB104909.1	-
Pyrus_pyrifolia_05	-	AB002140.1, AB002141.1, AB025421.1, AB045711.1, D88282.1	-
Pyrus_x_bretschneideri_01	-	-	Px_05
Pyrus_01	-	AB284262.1	Pcommunis_06

Tip Name	EST	NT	Genomic
Pyrus_02	-	-	Pcommunis_01, Pcommunis_08, Px_07
Pyrus_03	-	D49529.1	Pcommunis_02, Px_06
Pyrus_04	-	-	Pcommunis_05, Pcommunis_07, Px_08
Pyrus_05	-	AB426604.1, KF500076.1, KF500077.1, KF500078.1	-
Pyrus_06	-	AB002143.1	Px_03
Quercus_robur_01	FP024803.1, FP026336.1, FP027288.1, FP027904.1, FP030531.1	-	-
Quercus_01	FN698267.1, FN698652.1, FN701320.1, FN707157.1, FN726935.1, FN727424.1, FN735842.1, FN749412.1, FR631903.1, FR633503.1	-	-
Raphanus_sativus_02	-	-	Rsativus_10, Rsativus_11
Raphanus_01	EV529122.1, EV532941.1, EV537523.1, EV537599.1, EV541798.1, EV548667.1, EW716601.1, EW716869.1, EW721186.1, EW721990.1, EX747791.1, EX751897.1, EX758247.1, EX761651.1, EX772252.1, EX887881.1, EX891919.1, EY897856.1, EY927123.1, EY928590.1, EY931078.1, EY933002.1, EY933719.1, EY936647.1, EY937174.1, EY937436.1, EY940976.1, EY942779.1, EY946748.1, FD533241.1, FD539032.1, FD543137.1, FD544259.1, FD569252.1, FD570438.1, FD573492.1, FD966590.1, FD969503.1	-	Rsativus_02

Tip Name	EST	NT	Genomic
Raphanus_02	EV543436.1, EV546075.1,<br>EV547595.1, EW714617.1,<br>EW719449.1, EW723025.1,<br>EW725342.1, EW725377.1,<br>EW727831.1, EW728728.1,<br>EW730730.1, EW730759.1,<br>EX747025.1, EX747748.1,<br>EX751318.1, EX763865.1,<br>EX764017.1, EX768021.1,<br>EX770054.1, EX774312.1,<br>EX886832.1, EX888177.1,<br>EX892163.1, EY906100.1,<br>EY917495.1, EY921326.1,<br>EY923344.1, EY935557.1,<br>EY937115.1, EY941563.1,<br>EY947967.1, FD542897.1,<br>FD545265.1, FD549177.1,<br>FD552456.1, FD556095.1,<br>FD560447.1, FD564747.1,<br>FD570683.1, FD574645.1,<br>FD950616.1, FD954341.1,<br>FD964344.1, FD989950.1,<br>FY428434.1	-	-
Raphanus_03	EW735374.1, EX762349.1,<br>EX766624.1, EX897376.1,<br>EX901148.1, EX901971.1,<br>EY894773.1, EY898830.1,<br>FD943097.1, FD947092.1	-	Rsativus_05
Raphanus_04	EX762958.1, EX767119.1,<br>EX895670.1, EY894225.1,<br>EY898063.1, EY904738.1,<br>FD556340.1, FD568640.1,<br>FD572358.1, FD572951.1,<br>FD576014.1, FD951555.1,<br>FD955498.1, FD955762.1	-	Rsativus_12
Raphanus_05	EV536807.1, EV541172.1,<br>EX758032.1, EX761474.1,<br>EX764843.1, EY933373.1,<br>FD583073.1, FD970181.1	-	-
Raphanus_06	EV527105.1, EV531426.1,<br>FD534737.1, FD960160.1,<br>FD963621.1, FD964030.1,<br>FD974709.1, FD978468.1	-	Rsativus_09
Ricinus_communis_01	-	XM_002522396.1	Rcommunis_01

Tip Name	EST	NT	Genomic
Ricinus_communis_02	-	XM_002512884.1	Rcommunis_05
Ricinus_communis_03	-	XM_002522397.1	Rcommunis_02
Ricinus_communis_04	EG658351.1, EG660818.1, EG664696.1, GE635005.1	XM_002524508.1, XM_002524535.1	-
Sarcocapnos_crassifolia_01	JG643252.1	-	-
Sarracenia_leucophylla_01	-	AB872468.1	-
Saruma_henryi_01	DT599406.1	-	-
Saruma_henryi_02	DT576274.1, DT587986.1, DT590202.1, DT590500.1, DT598928.1, DT600891.1, DT602889.1, DT603017.1	-	-
Saussurea_medusa_01	FG269154.1	-	-
Selaginella_moellendorffii_01	-	XM_002960270.1, XM_002967350.1	Smoellend_01
Selaginella_moellendorffii_02	FE476369.1, FE476370.1, FE500619.1, FE500620.1, FE513434.1, FE513435.1	XM_002965953.1, XM_002989181.1	Smoellend_02
Senecio_chrysanthemifolius_01	DY658128.1	-	-
Sesamum_indicum_01	-	-	Sindicum_02
Sesamum_indicum_02	-	-	Sindicum_03
Setaria_italica_01	-	XM_004973464.1	Sitalica_07
Setaria_italica_02	-	XM_004958353.1	Sitalica_04
Setaria_italica_03	EC612152.1	XM_004970841.1	Sitalica_05
Setaria_italica_04	-	XM_004957426.1, XM_004957427.1	Sitalica_01
Setaria_italica_05	-	NM_001287232.1	Sitalica_02
Setaria_italica_06	-	XM_004958352.1	Sitalica_03
Setaria_italica_07	-	XM_004970840.1	Sitalica_06
Silene_latifolia_01	GH291844.1	-	-
Silene_latifolia_02	GH292195.1	-	-
Silene_latifolia_03	GH293331.1	-	-
Silene_latifolia_04	GH291783.1, GH293996.1	-	-
Siraitia_grosvenorii_01	-	HQ141616.1	-
Siraitia_grosvenorii_02	HS403338.1	-	-
Siraitia_grosvenorii_03	HS408153.1	-	-
Sisymbrium_irio_01	-	-	Sirio_01
Sisymbrium_irio_02	-	-	Sirio_02
Sisymbrium_irio_03	-	-	Sirio_03
Sisymbrium_irio_04	-	-	Sirio_04
Sisymbrium_irio_05	-	-	Sirio_05
Solanum_chacoense_01	-	X56896.1	-
Solanum_chacoense_02	-	X56897.1	-

Tip Name	EST	NT	Genomic
Solanum_chacoense_03	-	AF232304.1,<br>L36666.1	-
Solanum_chacoense_04	-	AF176533.1,<br>AF191732.1	-
Solanum_chilense_01	-	AB072469.1,<br>EF680106.1	-
Solanum_lycopersicum_01	-	XM_004229015.1	Slycopers_01
Solanum_melongena_01	FS074787.1, FS074788.1,<br>FS084880.1, FS084881.1	-	-
Solanum_melongena_02	FS027270.1, FS027286.1,<br>FS027520.1, FS027743.1,<br>FS027753.1, FS027970.1,<br>FS028991.1, FS030329.1,<br>FS030330.1, FS030348.1,<br>FS031360.1, FS032889.1,<br>FS079451.1, FS079459.1,<br>FS079816.1	-	-
Solanum_neorickii_01	-	AB072475.1	-
Solanum_pennellii_01	-	-	Spennelli_04
Solanum_peruvianum_01	-	AB072466.1	-
Solanum_peruvianum_02	-	S61768.1,<br>X76065.1	-
Solanum_peruvianum_03	-	Z26583.1	-
Solanum_peruvianum_04	-	Z26582.1	-
Solanum_peruvianum_05	-	D17325.1	-
Solanum_stenotomum_01	-	HM446648.1	-
Solanum_tuberosum_01	-	X62727.1	-
Solanum_tuberosum_02	-	XM_006344269.1	Stuberosu_01
Solanum_tuberosum_03	BE920237.1, BE920972.1,<br>BE923485.1, BF053199.1,<br>BF054468.1, BG096450.1,<br>BG591767.1	XM_006343488.1	Stuberosu_16
Solanum_01	-	AB072464.1,<br>AB072478.1,<br>S81597.1,<br>U28796.1	Shabrocha_02
Solanum_02	-	AB072477.1,<br>Z26581.1	Sarcanum_03

Tip Name	EST	NT	Genomic
Solanum_03	AI483500.1, AI483637.1, AI484924.1, AI484997.1, AI485790.1, AI487298.1, AI487581.1, AI487712.1, AI490252.1, AI899246.1, BE434000.1, BE441192.1, BF051673.1, BI930311.1, BP879013.1, BW690582.1, GO372869.1	XM_004242617.1	Sarcanum_09, Slycopers_10, Spennelli_10, Spimpinel_01
Solanum_04	-	XM_004236993.1, XM_006344205.1	Sarcanum_05, Shabrocha_07, Spennelli_01, Spimpinel_09
Solanum_05	AI484830.1, AI485206.1, AI486253.1, AI488432.1, AI489460.1, AI772676.1, AI775352.1	AJ536577.1, NM_001247266.1, XM_006355040.1, Y17444.1, Y17445.1	Sarcanum_12, Shabrocha_09, Slycopers_04, Spennelli_03
Solanum_06	BI210026.1, CK862438.1, CK864217.1, DB682061.1, DB694833.1, DN906554.1, DN906555.1, DV105079.2, FS192769.1, JG561767.1	AK329027.1, AM231403.1, NM_001247622.1, XM_006355041.1, Y17446.1	Sarcanum_11, Shabrocha_10, Slycopers_03, Spennelli_02, Spimpinel_10
Solanum_07	AW039494.1, AW216541.1, AW223027.1, AW224120.1, BE435686.1, BF098167.1, BF113095.1, BF187990.1, BG350033.1, BG597684.1, BP877116.1, BP888309.1, BQ119665.2, BW685467.1, BW685550.1, CK263423.1, CK263485.1, CK263486.1, CK271885.1, CK271886.1, CK276081.1, CK276082.1, CK276102.1, CK717429.1, CK718633.1, DB689435.1, DB689465.1, DB704869.1, DB711181.1, DN743819.1, DR034528.1, ES891495.1, ES893943.1, ES896627.1, FS194487.1	AK324819.1, AM408589.1, XM_004246194.1, XM_006344282.1	Sarcanum_04, Shabrocha_01, Spennelli_11, Spimpinel_02
Solanum_08	-	GU361149.1, JQ040817.1, U28795.1	-

Tip Name	EST	NT	Genomic
Solanum_09	-	L36464.1, L36667.1, XM_006347185.1	Stuberosu_09
Solanum_10	DN982172.1	XM_006367757.1	Stuberosu_04
Solanum_11	FG646836.1, FG647168.1	XM_006343638.1	Stuberosu_11
Solanum_12	AI484064.1, AI488160.1, AI488272.1, AW094303.1, AW928412.1, BE463383.1, JG567141.1	XM_004242616.1	Sarcanum_08, Shabrocha_04, Spennelli_09, Spimpinel_08
Solanum_13	AW034883.1, AW623083.1, BE432989.1, CK254404.1, CK254405.1, CK264609.1, CK264610.1, CV503834.1, DB681039.1, DB689334.1, DB711096.1, DB715473.1	AK320420.1, XM_004242409.1, XM_006366747.1, XM_006366748.1	Shabrocha_08, Slycopers_07, Spimpinel_07, Stuberosu_10
Sorghum_bicolor_01	CD206103.1, CD429506.1, CF769657.1, CN135898.1, CN137644.1, CN140516.1, CN141890.1, CN143042.1, CN143355.1, CN143680.1, CN143686.1, CN143787.1, CN143840.1, CN143847.1, CN144335.1, CN144345.1, CN144369.1, CN144379.1, CN144764.1, CN144945.1, CN145077.1, CN145269.1, CN145275.1, CN145415.1, CN145693.1, CN145826.1, CN146004.1, CN146317.1, CN147050.1, CN147051.1, CN147247.1, CN147542.1, CN148605.1, CN148770.1, CN149104.1, CN149588.1, CN150400.1, CN151162.1, CN151341.1, CN152348.1, CN152382.1, CN152782.1	XM_002460533.1, XM_002462696.1	Sbicolor_01
Sorghum_bicolor_02	-	XM_002462697.1	Sbicolor_02
Sorghum_bicolor_03	-	XM_002458873.1	Sbicolor_05
Sorghum_bicolor_04	-	XM_002445504.1	Sbicolor_06
Sorghum_bicolor_05	-	XM_002461025.1	-

Tip Name	EST	NT	Genomic
Sorghum_bicolor_06	AW671991.1, BE595388.1, BM317485.1, CD231761.1, CF427100.1, CF427912.1, CF482072.1, CN125135.1, CN125196.1, CN133430.1, CN135314.1, CN135382.1, CN142030.1, CX607577.1, CX613710.1, EH407676.1	XM_002458872.1	-
Spinacia_oleracea_01	-	-	Soleracea_02
Spinacia_oleracea_02	-	-	Soleracea_03
Spinacia_oleracea_03	-	-	Soleracea_04
Spinacia_oleracea_04	-	-	Soleracea_05
Spinacia_oleracea_05	-	-	Soleracea_06
Spinacia_oleracea_06	-	-	Soleracea_07
Spinacia_oleracea_07	-	-	Soleracea_08
Striga_hermonthica_01	FS464251.1, FS464325.1, FS474477.1, FS477979.1, FS481098.1, FS484749.1, FS496693.1, FS501666.1, FS505235.1, FS506507.1	-	-
Suaeda_glauca_01	HS411145.1	-	-
Syntrichia_ruralis_01	CN203860.1, CN204134.1, CN204378.1, CN206506.1	-	-
Taraxacum_kok-saghyz_01	DR400763.1, GO668307.1	-	-
Taraxacum_officinale_01	DY818026.1, DY820763.1, DY822438.1, DY823103.1, DY823518.1, DY825188.1, DY826122.1, DY828460.1, DY828503.1, DY830137.1, DY832759.1, DY832799.1	-	-
Taxus_baccata_01	-	-	Tbaccata_01
Thellungiella_parvula_01	-	-	Tparvula_01
Thellungiella_parvula_02	-	-	Tparvula_02
Thellungiella_parvula_03	-	-	Tparvula_03
Thellungiella_parvula_04	-	-	Tparvula_04
Thellungiella_parvula_05	-	-	Tparvula_05
Theobroma_cacao_01	-	XM_007011015.1	Tcacao_04
Theobroma_cacao_02	-	-	Tcacao_09
Theobroma_cacao_03	-	XM_007011024.1	Tcacao_10
Theobroma_cacao_04	-	XM_007011025.1	Tcacao_11
Theobroma_cacao_05	-	XM_007045626.1	Tcacao_15
Theobroma_cacao_06	-	XM_007046018.1	Tcacao_16
Theobroma_cacao_07	-	XM_007046019.1	Tcacao_17

Tip Name	EST	NT	Genomic
Theobroma_cacao_08	-	XM_007038786.1	Tcacao_19
Theobroma_cacao_09	-	XM_007038787.1	Tcacao_20
Theobroma_cacao_10	CU541598.1	XM_007099524.1	Tcacao_21
Theobroma_cacao_11	-	XM_007015690.1	Tcacao_22
Theobroma_cacao_12	-	XM_007011012.1	Tcacao_02
Theobroma_cacao_13	-	XM_007011082.1, XM_007011091.1	Tcacao_13
Theobroma_cacao_14	CU477139.1, CU483035.1, CU483081.1, CU511781.1, CU532484.1, CU572167.1, CU585846.1, CU631208.1	XM_007049456.1	Tcacao_01
Tinospora_cordifolia_01	JG646684.1, JG647636.1, JG647986.1, JG648665.1	-	-
Trifolium_01	FY467235.1	-	Tpratense_13
Triphysaria_pusilla_01	EY125139.1, EY125140.1, EY127480.1, EY129135.1, EY129136.1, EY137596.1	-	-
Triphysaria_pusilla_02	EY129378.1, EY129379.1, EY130399.1, EY130400.1, EY133768.1, EY133769.1	-	-
Triticum_aestivum_01	BE404484.1, BE446370.1, BE490784.1, BF474879.1, BM137478.1, BQ743175.1, BQ839366.1, CD880969.1, CD883497.1, CD934128.1, CD935410.1, CJ609574.1, CJ714149.1, CJ858914.1, CJ871783.1, CJ872106.1, CJ906652.1, CJ906949.1, CJ908004.1, CJ918841.1, CK162572.1, CK196093.1, CK204714.1, CK205063.1, CV765953.1, CV766315.1, CV768622.1, CV768966.1, CV773086.1, CV774799.1, CV776659.1, CV779426.1, GR303949.1, HX165334.1, HX165359.1	-	-
Triticum_aestivum_02	CA485852.1	-	-
Triticum_aestivum_03	CA486081.1, CA486574.1	-	-
Triticum_aestivum_04	-	-	Taestivum_02, Taestivum_04
Triticum_aestivum_05	-	-	Taestivum_17

**Tip Name**	**EST**	**NT**	**Genomic**
Triticum_aestivum_06	CD861839.1, CJ708490.1, CJ719265.1, CJ946214.1, CJ946215.1, CK163004.1, CK163696.1, CV768232.1, DR737634.1, DR738148.1, DR738150.1, DR738403.1, DR740799.1	AF495872.1, BT009061.1	Taestivum_05
Triticum_01	BE490205.1, BE606798.1, BJ234043.1, BJ239911.1, BJ255561.1, CD893232.1, CD902102.1, CJ551219.1, CJ553755.1, CJ578775.1, CJ658858.1, CJ661175.1, CJ661288.1, CJ679536.1, CJ685369.1, CV759414.1	AY528721.1	Taestivum_11, Taestivum_13, Turartu_03

Tip Name	EST	NT	Genomic
Triticum_02	AJ612369.1, AL823028.1, AM285636.1, BF473141.1, BJ260079.1, BJ265782.1, BQ743375.1, BQ789161.1, CD881383.1, CD881384.1, CD882147.1, CD883909.1, CJ559305.1, CJ562454.1, CJ562735.1, CJ562767.1, CJ563993.1, CJ568863.1, CJ581880.1, CJ582159.1, CJ583559.1, CJ585517.1, CJ586244.1, CJ594127.1, CJ666567.1, CJ669616.1, CJ669914.1, CJ671095.1, CJ675789.1, CJ687780.1, CJ688329.1, CJ688623.1, CJ689991.1, CJ691853.1, CJ692545.1, CJ700008.1, CJ859127.1, CJ871950.1, CJ947397.1, CJ959374.1, CK206023.1, CK208373.1, CK213974.1, CK214409.1, CK215112.1, CK216067.1, CV770507.1, CV778383.1, HX040363.1, HX125484.1, HX125504.1, HX129860.1, HX129887.1, HX153677.1, HX153705.1, HX173417.1, HX173443.1, HX197197.1, HX197227.1	AY517470.1	Taestivum_23, Taestivum_25, Turartu_07

**Tip Name**	**EST**	**NT**	**Genomic**
Triticum_03	AJ611563.1, AJ611608.1, BE517987.1, BF483414.1, BJ245521.1, BJ251405.1, BJ281674.1, BJ286768.1, BM135340.1, BQ744153.1, BQ788692.1, BQ788939.1, CD453589.1, CJ558947.1, CJ561158.1, CJ561508.1, CJ666219.1, CJ668379.1, CJ668718.1, CJ670731.1, CJ774086.1, CJ776063.1, CJ782372.1, CJ785705.1, CJ785802.1, CJ788035.1, CJ800098.1, CJ802864.1, CJ812927.1, CJ851388.1, CJ854294.1, CJ856468.1, CJ862882.1, CJ867055.1, CJ869140.1, CJ880523.1, CJ892522.1, CJ931854.1, CJ944152.1, CJ947181.1, CJ948255.1, CJ951307.1, CJ956082.1, CJ959346.1, CJ960332.1, CJ963289.1, CJ967949.1, CK214973.1, CK217856.1, CV763015.1, CV764218.1, CV765166.1, CV767594.1, CV772030.1, DR738977.1, DR739576.1, HX063273.1, HX066856.1, HX066887.1, HX085457.1, HX085490.1, HX093232.1, HX129715.1, HX129740.1, HX131240.1, HX131266.1, HX135004.1, HX135032.1, HX135505.1, HX141595.1, HX141620.1, HX142502.1, HX160163.1, HX160192.1, HX164719.1, HX164744.1, HX165229.1, HX165253.1, HX169286.1, HX169312.1, HX181395.1, HX194985.1, HX197879.1, HX197902.1, HX198440.1, HX198467.1, HX199264.1, HX199285.1, HX201646.1, HX201671.1	AK331025.1	Taestivum_12, Turartu_06

Tip Name	EST	NT	Genomic
Triticum_04	CJ713017.1, CJ725253.1, DR738947.1	-	Taestivum_01, Turartu_09
Vicia_faba_01	HX903971.1	-	-
Vicia_faba_02	HX908502.1	-	-
Vicia_faba_03	HX904792.1, HX906607.1, HX910721.1	-	-
Vigna_angularis_01	-	-	Vangulari_01
Vigna_angularis_02	-	-	Vangulari_02
Vigna_angularis_03	-	-	Vangulari_03
Vigna_unguiculata_01	FG831345.1, FG855444.1, FG862850.1, FG876898.1, FG890912.1, FG905198.1, FG922188.1, FG933616.1	-	-
Vigna_unguiculata_02	FF384683.1, FG820166.1, FG820167.1, FG822746.1, FG822747.1, FG904065.1, FG904066.1	-	-
Vigna_unguiculata_03	FF387861.1, FF393331.1	-	-
Vigna_01	FF390700.1, FF391881.1	-	Vangulari_04
Vitis_vinifera_01	CB971301.1, CB971379.1, CB971712.1, CB971792.1, EE090988.1, EE091145.1	XM_002275235.2, XM_003631287.1	Vvinifera_01
Vitis_vinifera_02	CF517014.1, EC949968.1, EC956207.1, EC966936.1, EE067827.1, EE073309.1, EE075421.1, EE085818.1, EE085819.1, FQ398871.1, FQ401152.1, FQ401647.1, FQ402530.1, FQ405462.1, FQ405932.1, FQ410560.1, FQ412815.1, FQ436505.1, FQ437451.1, FQ441979.1, FQ445037.1, FQ445959.1, FQ456713.1, FQ457149.1, FQ457897.1, FQ478200.1, FQ478812.1, FQ479428.1, FQ479646.1	FQ390131.1, FQ395263.1, FQ395755.1	-
Volvox_carteri_01	-	-	Vcarteri_01

Tip Name	EST	NT	Genomic
Volvox_carteri_02	FD805131.1, FD806574.1, FD818800.1, FD825639.1, FD827764.1, FD828354.1, FD833126.1, FD833658.1, FD835043.1, FD840627.1, FD848229.1, FD905506.1, FD908202.1, FD916973.1, FD922208.1, FD927491.1, FD930906.1	AB034248.1, XM_002946778.1, XM_002946965.1	Vcarteri_02
Wrightia_tinctoria_01	HS563211.1, HS568506.1	-	-
Xerophyta_humilis_01	JK689862.1	-	-
Yucca_filamentosa_01	DT580614.1	-	-
Zea_mays_01	AI770589.1, AI770873.1, DN211529.1, DN224237.1, FL204749.1, FL479180.1, FL480373.1, FM195325.1	AY105459.1, EU963069.1, NM_001156986.1	Zmays_02
Zea_mays_02	CF625213.1, EE681566.1, FL153019.1, FL153022.1, FL478795.1	NM_001157827.1	-
Zea_mays_03	BM079951.1, BM267242.1, BM267253.1, BM335972.1, BM340344.1, BM348991.1, BM379354.1, CB350711.1, CD438627.1, CF625647.1, CK347665.1, CO457224.1, CO458868.1, CO459248.1, CO459272.1, CO465949.1, DR790776.1, DR790777.1, DV033416.1, EB705702.1, EC888851.2, EC888852.2, EC896491.1, EE039174.1, EE185423.2, EE185424.2, EE190895.2, FL145112.1, FL145121.1, FL360470.1, FL372616.1, FL380890.1, FL381740.1, FL401476.1, FL402946.1, FL408597.1, FL415586.1	AY107950.1, BT019161.1, EU965778.1, EU967195.1, NM_001137533.1	Zmays_01

Tip Name	EST	NT	Genomic
Zea_mays_04	AI677179.1, BG316961.1, BG462434.1, BG462435.1, BG518285.1, BI358846.1, BI359343.1, BI595826.1, CA403807.1, CD437965.1, CD440226.1, CD441108.1, CD448106.1, CD968353.1, CD983691.1, CD983719.1, CD984812.1, CF025494.1, CF025498.1, CF025536.1, CF025571.1, CF025838.1, CF026064.1, CF026076.1, CF026515.1, CF636717.1, CK786736.1, CO445653.1, CO452833.1, CO457895.1, CO464056.1, CO468328.1, CO519706.1, DR794797.1, DR794798.1, DR820163.1, DR824140.1, DR957872.1, DR959209.1, DR965520.1, DR968127.1, DR968128.1, DT947106.1, DT947107.1, DV495357.1, DV516184.1, EB405655.1, EB707821.1, EB707822.1, EC906010.2, EE021517.2, EE029839.2, EE029840.2, EE159849.2, EE159850.2, FL002949.1, FL002952.1, FL002955.1, FL002957.1, FL002962.1, FL024976.1, FL157433.1, FL157434.1, FL157435.1, GR421186.1	AY107592.1, BT016738.1, EU962234.1, EU964116.1, NM_001137904.2	Zmays_03
Zea_mays_05	-	AY104404.2, BT083981.1, EU955694.1, EU959004.1, NM_001112600.1	Zmays_06
Zinnia_violacea_01	-	U19923.1	-
Zinnia_violacea_02	FM880519.1, FM880520.1, FM880521.1	U19924.1	-
Zizania_latifolia_01	-	-	Zlatifoli_04
Zizania_latifolia_02	-	-	Zlatifoli_05
Zizania_latifolia_03	-	-	Zlatifoli_06

Tip Name	EST	NT	Genomic
Zizania_latifolia_04	-	-	Zlatifoli_07, Zlatifoli_09
Zostera_marina_01	HS090062.1	-	-
Zostera_marina_02	AM768153.1, AM768501.1	-	-

Table S2: Genomes Examined

**Organism**	**Source**	**Used**[1]
Abies sibirica	Spruce Genome Project[2]	No
Actinidia chinensis	NCBI	Yes
Aegilops tauschii	NCBI	Yes
Aethionema arabicum	NCBI	Yes
Amborella trichopoda	Phytozome version 10.0.2[3]	Yes
Aquilaria agallochum	NCBI	Yes
Aquilegia coerulea	Phytozome version 10.0.2	Yes
Arabidopsis halleri	Phytozome version 10.0.2	Yes
Arabidopsis lyrata	Phytozome version 10.0.2	Yes
Arabidopsis thaliana	Phytozome version 10.0.2	Yes
Auxenochlorella protothecoides	NCBI	No
Azadirachta indica	NCBI	Yes
Azolla filiculoides	Fay-Wei Li, pers. comm.	No
Bathycoccus prasinos	NCBI BioProject (PRJNA233489)[4]	No
Beta vulgaris	NCBI	Yes
Betula nana	Dwarf Birch Genome Project[5]	No
Boechera stricta	Phytozome version 10.0.2	Yes
Brachypodium distachyon	Phytozome version 10.0.2	Yes
Brassica napus	NCBI	Yes
Brassica oleracea	NCBI	Yes
Brassica rapa	Phytozome version 10.0.2	Yes
Cajanus cajan	NCBI BioProject (PRJNA72815)[6]	Yes
Camelina sativa	NCBI	Yes
Cannabis sativa	NCBI	Yes
Capsella grandiflora	Phytozome version 10.0.2	Yes
Capsella rubella	Phytozome version 10.0.2	Yes
Capsicum annuum	SGN[7]	No
Capsicum chinense	SGN	Yes
Carica papaya	Phytozome version 10.0.2	Yes
Chlamydomonas reinhardtii	Phytozome version 10.0.2	Yes
Chlorella variabilis	JGI Genome Portal[8]	No
Chondrus crispus	NCBI	No
Cicer arietinum	NCBI	Yes
Citrullus lanatus	Cucurbit Genomics Database[9]	Yes
Citrus clementina	Phytozome version 10.0.2	Yes

[1]At least one complete RNase sequence without ambiguities and premature stop codons was found.
[2]http://congenie.org/start
[3]http://phytozome.jgi.doe.gov/pz/portal.html
[4]http://www.ncbi.nlm.nih.gov/bioproject/PRJNA233489
[5]http://www.birchgenome.org
[6]http://www.ncbi.nlm.nih.gov/bioproject?term=PRJNA72815
[7]ftp://ftp.solgenomics.net/genomes
[8]http://genome.jgi.doe.gov
[9]ftp://www.icugi.org/pub/genome/watermelon

Organism	Source	Used
Citrus sinensis	Phytozome version 10.0.2	Yes
Cleome hassleriana	NCBI	Yes
Coccomyxa subellipsoidea	Phytozome version 10.0.2	No
Coffea canephora	Coffee Genome Hub[10]	Yes
Cucumis melo	NCBI	Yes
Cucumis sativus	Phytozome version 10.0.2	Yes
Cyanidioschyzon merolae	*Cyanidioschyzon merolae* Genome Project[11]	No
Cyanophora paradoxa	*Cyanophora* Genome Project[12]	No
Dianthus caryophyllus	NCBI	Yes
Ectocarpus siliculosus	NCBI BioProject (PRJEA42625)[13]	No
Elaeis guineensis	NCBI	Yes
Elaeis oleifera	NCBI	Yes
Ensete ventricosum	NCBI	Yes
Eucalyptus camaldulensis	NCBI	Yes
Eucalyptus grandis	Phytozome version 10.0.2	Yes
Eutrema salsugineum	Phytozome version 10.0.2	Yes
Fragaria iinumae	NCBI	Yes
Fragaria nipponica	NCBI	Yes
Fragaria nubicola	NCBI	Yes
Fragaria orientalis	NCBI	Yes
Fragaria vesca	Phytozome version 10.0.2	Yes
Fragaria × ananassa	NCBI	Yes
Fraxinus excelsior	NCBI	Yes
Galdieria sulphuraria	NCBI	No
Genlisea aurea	NCBI	No
Glycine max	Phytozome version 10.0.2	Yes
Gnetum gnemon	Spruce Genome Project	No
Gossypium arboreum	CottonGen[14]	Yes
Gossypium raimondii	Phytozome version 10.0.2	Yes
Hevea brasiliensis	NCBI	Yes
Hordeum pubiflorum	NCBI	Yes
Hordeum vulgare	Ensembl Plants[15]	Yes
Jatropha curcas	NCBI	Yes
Juniperus communis	Spruce Genome Project	No
Klebsormidium flaccidum	NCBI	No
Lactuca sativa	NCBI	Yes
Lagenaria siceraria	NCBI	Yes
Leavenworthia alabamica	NCBI	Yes

[10] http://coffee-genome.org/coffeacanephora
[11] http://merolae.biol.s.u-tokyo.ac.jp/download
[12] http://cyanophora.rutgers.edu/cyanophora/blast.php
[13] http://www.ncbi.nlm.nih.gov/bioproject/PRJEA42625
[14] ftp://ftp.bioinfo.wsu.edu/www.cottongen.org/Gossypium_arboreum
[15] ftp://ftp.ensemblgenomes.org/pub/plants/release-22

Organism	Source	Used
Leersia perrieri	NCBI	Yes
Linum usitatissimum	Phytozome version 10.0.2	Yes
Lotus japonicus	Kazusa DNA Research Institute[16]	No
Lupinus angustifolius	NCBI	Yes
Malus × *domestica*	Phytozome version 10.0.2	Yes
Manihot esculenta	Phytozome version 10.0.2	No
Marchantia polymorpha	Phytozome version 12	Yes[17]
Medicago truncatula	Phytozome version 10.0.2	Yes
Micromonas pusilla	Phytozome version 10.0.2	No
Mimulus guttatus	Phytozome version 10.0.2	Yes
Morus notabilis	NCBI	Yes
Musa acuminata	NCBI	Yes
Musa balbisiana	Banana Genome Hub[18]	Yes
Nelumbo nucifera	NCBI	Yes
Nicotiana benthamiana	SGN	Yes
Nicotiana otophora	NCBI	Yes
Nicotiana sylvestris	NCBI	Yes
Nicotiana tabacum	SGN	No
Nicotiana tomentosiformis	NCBI	Yes
Oryza barthii	NCBI	Yes
Oryza brachyantha	NCBI	Yes
Oryza glaberrima	NCBI	Yes
Oryza glumipatula	NCBI	Yes
Oryza meridionalis	NCBI	Yes
Oryza nivara	NCBI	Yes
Oryza punctata	NCBI	Yes
Oryza sativa	Phytozome version 10.0.2	Yes
Ostreococcus lucimarinus	Phytozome version 10.0.2	No
Ostreococcus sp. RCC809	JGI Genome Portal	No
Ostreococcus tauri	JGI Genome Portal	No
Panicum hallii	Phytozome version 10.0.2	Yes
Panicum virgatum	Phytozome version 10.0.2	Yes
Phaseolus vulgaris	Phytozome version 10.0.2	Yes
Phoenix dactylifera	NCBI	Yes
Phyllostachys edulis	NCGR[19]	Yes
Physcomitrella patens	Phytozome version 10.0.2	Yes
Picea abies	Spruce Genome Project	Yes
Pinus sylvestris	Spruce Genome Project	No
Pinus taeda	Conifer Genome Network[20]	Yes

[16] http://www.kazusa.or.jp/lotus
[17] Genome was searched after the principal analyses were completed. Sequences not on tree.
[18] http://banana-genome.cirad.fr/content/musa-balbisiana-pisang-klutuk–wulung
[19] http://202.127.18.221/bamboo
[20] http://www.pinegenome.org/pinerefseq

Organism	Source	Used
Populus euphratica	NCBI	Yes
Populus grandidentata	Dendrome[21]	Yes
Populus tremula	Dendrome	Yes
Populus tremuloides	Dendrome	Yes
Populus trichocarpa	Phytozome version 10.0.2	Yes
Porphyridium purpureum	*Porphyridium purpureum* Genome Project[22]	No
Prunus mume	NCBI	Yes
Prunus persica	Phytozome version 10.0.2	Yes
Pyrus communis	Genome Database for Rosaceae[23]	Yes
Pyrus × bretschneideri	NCBI	Yes
Raphanus sativus	NCBI	Yes
Ricinus communis	Phytozome version 10.0.2	Yes
Salvinia cucullata	Fay-Wei Li, pers. comm.	No
Selaginella moellendorffii	Phytozome version 10.0.2	Yes
Sesamum indicum	NCBI	Yes
Setaria italica	Phytozome version 10.0.2	Yes
Sisymbrium irio	NCBI	Yes
Solanum arcanum	NCBI	Yes
Solanum habrochaites	NCBI	Yes
Solanum lycopersicum	Phytozome version 10.0.2	Yes
Solanum pennellii	ENA[24] (HG975439 to HG975452)	Yes
Solanum pimpinellifolium	SGN	Yes
Solanum tuberosum	Phytozome version 10.0.2	Yes
Sorghum bicolor	Phytozome version 10.0.2	Yes
Spinacia oleracea	NCBI	Yes
Spirodela polyrhiza	Phytozome version 10.0.2	No
Taxus baccata	Spruce Genome Project	Yes
Thellungiella parvula	NCBI[25]	Yes
Theobroma cacao	Phytozome version 10.0.2	Yes
Trifolium pratense	NCBI	Yes
Triticum aestivum	Ensembl Plants	Yes
Triticum urartu	NCBI	Yes
Vigna angularis	NCBI	Yes
Vitis vinifera	Phytozome version 10.0.2	Yes
Volvox carteri	Phytozome version 10.0.2	Yes
Zea mays	Phytozome version 10.0.2	Yes
Zizania latifolia	NCBI	Yes

[21] http://dendrome.ucdavis.edu
[22] http://cyanophora.rutgers.edu/porphyridium
[23] http://www.rosaceae.org/species/pyrus/pyrus_communis
[24] European Nucleotide Archive: http://www.ebi.ac.uk/ena
[25] http://www.ncbi.nlm.nih.gov/nuccore/AFAN00000000.1

Table S3: Studies of Expression Patterns and Function of T2/S-RNases

Class	Gene	Protein	Tip Name	References
I	*av-I*	AV-I	Aldrovanda_vesiculosa_01	Nishimura et al. 2014
I	*cf-I*	CF-I	Cephalotus_follicularis_01	Nishimura et al. 2013
I	*da-I*	DA-I	Drosera_adelae_01	Okabe et al. 2005<br>Arai et al. 2015
I	*dm-I*	DM-I	Dionaea_muscipula_01	Nishimura et al. 2013
I	*GAR-RNase*	GAR-RNase	Hordeum_04	Rogers & Rogers 1999
I	*nb-I*	NB-I	Nepenthes_bicalcarata_01	Nishimura et al. 2014
I	*NGR1*	RNase NW	Nicotiana_03	Kariu et al. 1998
I	*NGR3*	RNase NT	Nicotiana_01	Hino, Kawano & Kimura 2002<br>Kurata et al. 2002<br>Kawano et al. 2006
I	*non-S-RNase*	non-S-RNase	Pyrus_03	Norioka et al. 1996
I	*OsRNS1*	OsRNS1	Oryza_02	MacIntosh et al. 2010
I	 *OsRNS3*	RNase OS<br>OsRNS3	Oryza_01	Iwama et al. 2001<br>MacIntosh et al. 2010
I	 *OsRRP*<br>*OsRNS4*	 OsRRP<br>OsRNS4	Oryza_07	Ohkama-Ohtsu et al. 2004<br>Wei et al. 2006<br>MacIntosh et al. 2010<br>Zheng et al. 2014
I	*OsRNS5*	OsRNS5	Oryza_08	MacIntosh et al. 2010
I	*OsRNS7*	OsRNS7	Oryza_05	MacIntosh et al. 2010
I	*OsRNS8*	OsRNS8	Oryza_06	MacIntosh et al. 2010
I	*PD1*	RNase PD1	Prunus_01	Van Nerum et al. 2000
I	*Phy1*	Phy1	Petunia_x_hybrida_03	Hillwig et al. 2010
I	*RNaseLE*	RNase LE	Solanum_05	Nürnberger et al. 1990<br>Jost et al. 1991<br>Lers et al. 1998<br>Tanaka et al. 2000<br>Groß, Wasternack & Köck 2004<br>Köck et al. 2004
I	*RNaseLX*	RNase LX	Solanum_06	Löffler et al. 1992<br>Lers et al. 1998<br>Köck, Stenzel & Zimmer 2006<br>Lers et al. 2006
I	*RNaseNE*	RNase NE	Nicotiana_04	Dodds, Clarke & Newbigin 1996<br>Hugot et al. 2002
I	*RNasePD2*	RNase PD2	Prunus_08	Ma & Oliveira 2000

Class	Gene	Protein	Tip Name	References
I	*RNS1*	RNS1	Arabidopsis_02	Bariola et al. 1994<br>Bariola, MacIntosh & Green 1999<br>LeBrasseur et al. 2002<br>Hillwig et al. 2008<br>Hillwig et al. 2011<br>Nishimura et al. 2014
I	*RNS3*	RNS3	Arabidopsis_04	Bariola et al. 1994<br>Hillwig et al. 2011
I	*rsh1*	RSH1	Hordeum_03	Gausing 2000
I	*sl-I*	SL-I	Sarracenia_leucophylla_01	Nishimura et al. 2014
I	*ZRNaseI*	ZRNaseI	Zinnia_violacea_01	Ye & Droste 1996
I	*ZRNaseII*	ZRNaseII	Zinnia_violacea_02	Ye & Droste 1996
II	*AhSL28*	AhSL28	Antirrhinum_hispanicum_05	Liang et al. 2002
II	*CalsepRRP*	CalsepRRP	Calystegia_sepium_01	Van Damme et al. 2000<br>Rabijns et al. 2002
II	*CgSL2*	CgSL2	Citrus_04	Chai et al. 2011
II	*NGR2*	NGR2	Nicotiana_02	Kurata et al. 2002
II	*OsRNS2*	OsRNS2	Oryza_03	MacIntosh et al. 2010
II	*OsRNS6*	OsRNS6	Oryza_04	MacIntosh et al. 2010
II	*RNaseLER*	RNase LER	Solanum_07	Köthke & Köck 2011
II	*RNS2*	RNS2	Arabidopsis_03	Taylor et al. 1993<br>Bariola, MacIntosh & Green 1999<br>Hillwig et al. 2011
III	*AhRNase29*	AhRNase29	Antirrhinum_hispanicum_06	Liang, Huang & Xue 2003
III	*GMP*	GMP	Panax_01	Kim et al. 2004
III	*non-S63-RNase*<br>*NnSR1*	non-S63-RNase<br>NnSR1	Nicotiana_alata_06	Roldán, Quiroga & Goldraij 2010<br>Rojas, Roldán & Goldraij 2013
III	*p43*	P43	Pisum_sativum_01	Gaikwad et al. 1999
III	*Phy3*	Phy3	Petunia_x_hybrida_02	Hillwig et al. 2010
III	*Phy4*	Phy4	Petunia_x_hybrida_01	Hillwig et al. 2010

References

Arai N, Nishimura E, Kikuchi Y, Ohyama T. **2015**. Functional analyses of carnivorous plant-specific amino acid residues in S-like ribonucleases. *Biochemical and Biophysical Research Communications* **465**:108–112. doi:10.1016/j.bbrc.2015.07.139.

Bariola PA, Howard CJ, Taylor CB, Verburg MT, Jaglan VD, Green PJ. **1994**. The *Arabidopsis* ribonuclease gene *RNS1* is tightly controlled in response to phosphate limitation. *The Plant Journal* **6**:673–685. doi:10.1046/j.1365-313X.1994.6050673.x.

Bariola PA, MacIntosh GC, Green PJ. **1999**. Regulation of S-like ribonuclease levels in *Arabidopsis*. Antisense inhibition of *RNS1* or *RNS2* elevates anthocyanin accumulation. *Plant Physiology* **119**:331–342. doi:10.1104/pp.119.1.331.

Chai L, Ge X, Xu Q, Deng X. **2011**. *CgSL2*, an S-like RNase gene in 'Zigui shatian' pummelo (*Citrus grandis* Osbeck), is involved in ovary senescence. *Molecular Biology Reports* **38**:1–8. doi:10.1007/s11033-010-0070-x.

Dodds PN, Clarke AE, Newbigin E. **1996**. Molecular characterisation of an S-like RNase of *Nicotiana alata* that is induced by phosphate starvation. *Plant Molecular Biology* **31**:227–238. doi:10.1007/BF00021786.

Gaikwad A, Tewari KK, Kumar D, Chen W, Mukherjee SK. **1999**. Isolation and characterisation of the cDNA encoding a glycosylated accessory protein of pea chloroplast DNA polymerase. *Nucleic Acids Research* **27**:3120–3129. doi:10.1093/nar/27.15.3120.

Gausing K. **2000**. A barley gene (*rsh1*) encoding a ribonuclease S-like homologue specifically expressed in young light-grown leaves. *Planta* **210**:574–579. doi:10.1007/s004250050046.

Groß N, Wasternack C, Köck M. **2004**. Wound-induced *RNaseLE* expression is jasmonate and systemin independent and occurs only locally in tomato (*Lycopersicon esculentum* cv. Lukullus). *Phytochemistry* **65**:1343–1350. doi:10.1016/j.phytochem.2004.04.036.

Hillwig MS, Contento AL, Meyer A, Ebany D, Bassham DC, Macintosh GC. **2011**. RNS2, a conserved member of the RNase T2 family, is necessary for ribosomal RNA decay in plants. *Proceedings of the National Academy of Sciences* **108**:1093–1098. doi:10.1073/pnas.1009809108.

Hillwig MS, LeBrasseur ND, Green PJ, MacIntosh GC. **2008**. Impact of transcriptional, ABA-dependent, and ABA-independent pathways on wounding regulation of *RNS1* expression. *Molecular Genetics and Genomics* **280**:249. doi:10.1007/s00438-008-0360-3.

Hillwig MS, Liu X, Liu G, Thornburg RW, MacIntosh GC. **2010**. *Petunia* nectar proteins have ribonuclease activity. *Journal of Experimental Botany* **61**:2951–2965. doi:10.1093/jxb/erq119.

Hino M, Kawano S, Kimura M. **2002**. Expression of *Nicotiana glutinosa* ribonucleases in *Escherichia coli*. *Bioscience, Biotechnology, and Biochemistry* **66**:910–912. doi:10.1271/bbb.66.910.

Hugot K, Ponchet M, Marais A, Ricci P, Galiana E. **2002**. A tobacco S-like RNase inhibits hyphal elongation of plant pathogens. *Molecular plant-microbe interactions* **15**:243–250. doi:10.1094/MPMI.2002.15.3.243.

Iwama M, Ogawa Y, Yamagishi M, Itagaki T, Inokuchi N, Koyama T, Imai R, Ohgi K, Tsuji T, Irie M. **2001**. Amino acid sequence and characterization of a rice bran ribonuclease. *Biological and Pharmaceutical Bulletin* **24**:760–766. doi:10.1248/bpb.24.760.

Jost W, Bak H, Glund K, Terpstra P, Beintema JJ. **1991**. Amino acid sequence of an extracellular, phosphate-starvation-induced ribonuclease from cultured tomato (*Lycopersicon esculentum*) cells. *European Journal of Biochemistry* **198**:1–6. doi:10.1111/j.1432-1033.1991.tb15978.x.

Kariu T, Sano K, Shimokawa H, Itoh R, Yamasaki N, Kimura M. **1998**. Isolation and characterization of a wound-inducible ribonuclease from *Nicotiana glutinosa* leaves. *Bioscience, Biotechnology, and Biochemistry* **62**:1144–1151. doi:10.1271/bbb.62.1144.

Kawano S, Kakuta Y, Nakashima T, Kimura M. **2006**. Crystal structures of the *Nicotiana glutinosa* ribonuclease NT in complex with nucleoside monophosphates. *The Journal of Biochemistry* **140**:375–381. doi:10.1093/jb/mvj164.

Kim SI, Kweon SM, Kim EA, Kim JY, Kim S, Yoo JS, Park YM. **2004**. Characterization of RNase-like major storage protein from the ginseng root by proteomic approach. *Journal of Plant Physiology* **161**:837–845. doi:10.1016/j.jplph.2004.01.001.

Köck M, Groß N, Stenzel I, Hause G. **2004**. Phloem-specific expression of the wound-inducible Ribonuclease LE from tomato (*Lycopersicon esculentum cv. Lukullus*). *Planta* **219**:233–242. doi:10.1007/s00425-004-1227-4.

Köck M, Stenzel I, Zimmer A. **2006**. Tissue-specific expression of tomato Ribonuclease LX during phosphate starvation-induced root growth. *Journal of Experimental Botany* **57**:3717. doi:10.1093/jxb/erl124.

Köthke S, Köck M. **2011**. The *Solanum lycopersicum* RNaseLER is a class II enzyme of the RNase T2 family and shows preferential expression in guard cells. *Journal of Plant Physiology* **168**:840–847. doi:10.1016/j.jplph.2010.11.012.

Kurata N, Kariu T, Kawano S, Kimura M. **2002**. Molecular cloning of cDNAs encoding ribonuclease-related proteins in *Nicotiana glutinosa* leaves, as induced in response to wounding or to TMV-infection. *Bioscience, Biotechnology, and Biochemistry* **66**:391–397. doi:10.1271/bbb.66.391.

LeBrasseur ND, MacIntosh GC, Pérez-Amador MA, Saitoh M, Green PJ. **2002**. Local and systemic wound-induction of RNase and nuclease activities in *Arabidopsis*: RNS1 as a marker for a JA-independent systemic signaling pathway. *The Plant Journal* **29**:393–403. doi:10.1046/j.1365-313x.2002.01223.x.

Lers A, Khalchitski A, Lomaniec E, Burd S, Green PJ. **1998**. Senescence-induced RNases in tomato. *Plant Molecular Biology* **36**:439–449. doi:10.1023/A:1005993024161.

Lers A, Sonego L, Green PJ, Burd S. **2006**. Suppression of LX ribonuclease in tomato results in a delay of leaf senescence and abscission. *Plant Physiology* **142**:710–721. doi:10.1104/pp.106.080135.

Liang L, Huang J, Xue Y. **2003**. Identification and evolutionary analysis of a relic *S-RNase* in *Antirrhinum*. *Sexual Plant Reproduction* **16**:17–22. doi:10.1007/s00497-003-0168-6.

Liang L, Lai Z, Ma W, Zhang Y, Xue Y. **2002**. *AhSL28*, a senescence-and phosphate starvation-induced S-like RNase gene in *Antirrhinum*. *Biochimica et Biophysica Acta (BBA) - Gene Structure and Expression* **1579**:64–71. doi:10.1016/S0167-4781(02)00507-9.

Löffler A, Abel S, Jost W, Beintema JJ, Glund K. **1992**. Phosphate-regulated induction of intracellular ribonucleases in cultured tomato (*Lycopersicon esculentum*) cells. *Plant Physiology* **98**:1472–1478. doi:10.1104/pp.98.4.1472.

Ma RC, Oliveira MM. **2000**. The *RNase PD2* gene of almond (*Prunus dulcis*) represents an evolutionarily distinct class of S-like RNase genes. *Molecular and General Genetics* **263**:925–933. doi:10.1007/s004380000258.

MacIntosh GC, Hillwig MS, Meyer A, Flagel L. **2010**. RNase T2 genes from rice and the evolution of secretory ribonucleases in plants. *Molecular Genetics and Genomics* **283**:381–396. doi:10.1007/s00438-010-0524-9.

Nishimura E, Jumyo S, Arai N, Kanna K, Kume M, Nishikawa Ji, Tanase Ji, Ohyama T. **2014**. Structural and functional characteristics of S-like ribonucleases from carnivorous plants. *Planta* **240**:147–159. doi:10.1007/s00425-014-2072-8.

Nishimura E, Kawahara M, Kodaira R, Kume M, Arai N, Nishikawa Ji, Ohyama T. **2013**. S-like ribonuclease gene expression in carnivorous plants. *Planta* **238**:955–967. doi:10.1007/s00425-013-1945-6.

Norioka N, Norioka S, Ohnishi Y, Ishimizu T, Oneyama C, Nakanishi T, Sakiyama F. **1996**. Molecular cloning and nucleotide sequences of cDNAs encoding S-allele specific stylar RNases in a self-incompatible cultivar and its self-compatible mutant of japanese pear, *Pyrus pyrifolia* Nakai. *The Journal of Biochemistry* **120**:335. doi:10.1093/oxfordjournals.jbchem.a021418.

Nürnberger T, Abel S, Jost W, Glund K. **1990**. Induction of an extracellular ribonuclease in cultured tomato cells upon phosphate starvation. *Plant Physiology* **92**:970–976. doi:10.1104/pp.92.4.970.

Ohkama-Ohtsu N, Kishimoto N, Yazaki J, Fujii F, Shinbo K, Shimatani Z, Nagata Y, Hashimoto A, Ohta T, Sato Y, Honda S, Yamamoto K, Sakata K, Sasaki T, Kikuchi S, Hayashi H, Yoneyama T, Fujiwara T. **2004**. Upregulation of the genes for ferritin, RNase, and DnaJ in leaves of rice plants in response to sulfur deficiency. *Soil Science and Plant Nutrition* **50**:771–775. doi:10.1080/00380768.2004.10408534.

Okabe T, Iwakiri Y, Mori H, Ogawa T, Ohyama T. **2005**. An S-like ribonuclease gene is used to generate a trap-leaf enzyme in the carnivorous plant *Drosera adelae*. *FEBS Letters* **579**:5729–5733. doi:10.1016/j.febslet.2005.09.043.

Rabijns A, Verboven C, Rougé P, Barre A, Van Damme EJM, Peumans WJ, De Ranter CJ. **2002**. Structure of an RNase-related protein from *Calystegia sepium*. *Acta Crystallographica Section D* **58**:627–633. doi:10.1107/S090744490200255X.

Rogers SW, Rogers JC. **1999**. Cloning and characterization of a gibberellin-induced RNase expressed in barley aleurone cells. *Plant Physiology* **119**:1457–1464. doi:10.1104/pp.119.4.1457.

Rojas HJ, Roldán JA, Goldraij A. **2013**. NnSR1, a class III non-S-RNase constitutively expressed in styles, is induced in roots and stems under phosphate deficiency in *Nicotiana alata*. *Annals of Botany* **112**:1351. doi:10.1093/aob/mct207.

Roldán JA, Quiroga R, Goldraij A. **2010**. Molecular and genetic characterization of novel S-RNases from a natural population of *Nicotiana alata*. *Plant Cell Reports* **29**:735–746. doi:10.1007/s00299-010-0860-6.

Tanaka N, Arai J, Inokuchi N, Koyama T, Ohgi K, Irie M, Nakamura KT. **2000**. Crystal structure of a plant ribonuclease, RNase LE. *Journal of Molecular Biology* **298**:859–873. doi:10.1006/jmbi.2000.3707.

Taylor CB, Bariola PA, DelCardayré SB, Raines RT, Green PJ. **1993**. RNS2: a senescence-associated RNase of *Arabidopsis* that diverged from the S-RNases before speciation. *Proceedings of the National Academy of Sciences* **90**:5118–5122. doi:10.1073/pnas.90.11.5118.

Van Damme EJM, Hao Q, Barre A, Rougé P, Van Leuven F, Peumans WJ. **2000**. Major protein of resting rhizomes of *Calystegia sepium* (hedge bindweed) closely resembles plant RNases but has no enzymatic activity. *Plant Physiology* **122**:433–446. doi:10.1104/pp.122.2.433.

Van Nerum I, Certal AC, Oliveira MM, Keulemans J, Broothaerts W. **2000**. PD1, an S-like RNase gene from a self-incompatible cultivar of almond. *Plant Cell Reports* **19**:1108–1114. doi:10.1007/s002990000235.

Wei JY, Li AM, Li Y, Wang J, Liu XB, Liu LS, Xu ZF. **2006**. Cloning and characterization of an RNase-related protein gene preferentially expressed in rice stems. *Bioscience, Biotechnology, and Biochemistry* **70**:1041–1045. doi:10.1271/bbb.70.1041.

Ye ZH, Droste DL. **1996**. Isolation and characterization of cDNAs encoding xylogenesis-associated and wounding-induced ribonucleases in *Zinnia elegans*. *Plant Molecular Biology* **30**:697–709. doi:10.1007/BF00019005.

Zheng J, Wang Y, He Y, Zhou J, Li Y, Liu Q, Xie X. **2014**. Overexpression of an S-like ribonuclease gene, *OsRNS4*, confers enhanced tolerance to high salinity and hyposensitivity to phytochrome-mediated light signals in rice. *Plant Science* **214**:99–105. doi:10.1016/j.plantsci.2013.10.003.
